# The postoperative analgesic efficacy of three peripheral nerve blocks in hip fracture surgery: a systematic review and meta-analysis of randomised trials

**DOI:** 10.1007/s00402-025-06139-6

**Published:** 2026-01-06

**Authors:** Kaja Vermazen, Anna Niblett, Chloe Thomson, Alexandra Tebbett

**Affiliations:** 1 Warwick Medical School, University of Warwick, Coventry, UK; 2 Oxford University Hospitals NHS Trust, Oxford, UK

**Keywords:** Regional anaesthesia, Peripheral nerve block, Orthopaedic surgery, Postoperative pain, Hip fracture

## Abstract

**Introduction:**

Anaesthesia for hip fracture surgery is often supplemented with a peripheral nerve block (PNB) to reduce postoperative pain. Common PNBs include fascia-iliaca compartment block (FICB) and femoral nerve block (FNB). Since the introduction of the pericapsular nerve group (PENG) block, debate continues as to which technique provides superior analgesia. This review aimed to compare the postoperative analgesic efficacy of three PNBs when administered perioperatively to adult hip fracture patients.

**Methods:**

CINAHL, Cochrane CENTRAL, Embase, Medline, Web of Science and Google Scholar were searched in April 2025. Statistical analysis was performed using a random-effects model.

**Results:**

19 randomised trials (1059 patients) were included. Pain scores between PENG and FICB at 6, 12 and 24 h were not significantly different. Compared to FNB, PENG significantly lowered pain scores at 6 h (*P* = 0.004). Opioid consumption in the 24 h postoperative period was significantly lower in PENG than FICB (*P* = 0.02), but not in FNB. No outcome reached the minimal clinically important difference. Evidence was graded very low to moderate.

**Conclusion:**

There is insufficient evidence to state superiority of PENG over FICB or FNB when used perioperatively in patients undergoing hip fracture repair. No included studies reported time to mobilisation, highlighting a significant evidence gap in existing primary research. Further high-quality, sufficiently powered randomised trials are still needed.

**Supplementary Information:**

The online version contains supplementary material available at 10.1007/s00402-025-06139-6.

## Introduction

Sing et al. [1] project the incidence of hip fractures is likely to increase 100% by the year 2050. Optimisation of perioperative care for hip fracture patients is, therefore, a prominent matter of public health interest. The Association of Anaesthetists recommend that patients undergoing hip fracture fixation in the UK receive a perioperative peripheral nerve block in addition to spinal or general anaesthesia, to better control postoperative pain [2].

Peripheral nerve blockade for hip fractures is commonly achieved by administering ultrasound-guided fascia-iliaca compartment block (FICB) or femoral nerve block (FNB) [2]. In 2018, the pericapsular nerve group (PENG) block was described by Girón-Arango et al. [3]. The introduction of the PENG block proposed not only improved analgesia but preserved motor function. This contrasts the quadriceps weakness patients may experience with the use of alternative PNBs, leading to delayed mobilisation [3].

Previous reviews of this topic have focused on blocks administered in the emergency department; in these, PENG was deemed to potentially be superior, but confidence was low [4, 5]. To our knowledge, there are no reviews that compare these three blocks when administered in the immediate perioperative period.

This review hypothesises that PENG may provide superior postoperative analgesia in hip fracture patients compared to FICB or FNB, when administered in the perioperative period using ultrasound guidance, as a single injection technique.

## Methods

A project protocol was originally registered with PROSPERO under the reference CRD42024597296 on the 9th of October 2024 [6]. This review was conducted and reported in accordance with the Preferred Reporting Items for Systematic Reviews and Meta-Analyses (PRISMA) guidelines [7].

### Search strategy & information sources

CINAHL (EbscoHost), Cochrane CENTRAL, Embase (Ovid), Medline (Ovid), Web of Science (Clarivate) and Google Scholar were searched for randomised trials comparing two or all of PENG, FICB and FNB. Search strategies were designed by researchers KV & AN and approved by an expert librarian (Supplementary Files 1). Searches were limited by date from and inclusive of January 2014 to 27th April 2025. This date range was selected to return recent studies relating to FNB and FICB that weren’t significantly older than studies including PENG block, first described in 2018. Searches were initially carried out on the 21 st October 2024 and repeated on 27th April 2025 using the same strategy to include all relevant studies.

### Selection process & eligibility

Returned searches were initially stored in Endnote [8], and duplicates were removed before uploading the remaining references into Rayyan [9]. Two reviewers (KV and AN) independently screened abstracts and full texts.

Records for inclusion met the following criteria: participants aged 16 or over, undergoing surgery for hip fracture, receiving PENG, FICB or FNB perioperatively, studies comparing two or three included blocks, studies measuring our primary or secondary outcome, randomised control trial study design, studies available in English, published between January 2014 and April 2025, full-length text articles in peer-reviewed journals.

Studies were excluded when any of the exclusion criteria were met: patients with acetabular/pelvic fractures, comparison made to no block, local anaesthesia infiltration by surgeon, elective hip replacements, blocks performed in the emergency department, dose comparison studies, blocks performed using landmark technique or nerve stimulator, blocks maintained via continuous catheter infusion and study design other than randomised trials. Studies that failed to report any of the selected outcomes were also excluded.

Where there was uncertainty in the record’s ability to meet the criteria, the full text was reviewed. Conflicts were tracked in Rayyan and discussed between the two principal researchers. Where a conflict could not be resolved, two further independent researchers contributed to the final decision.

### Primary and secondary outcomes

The primary outcome was postoperative pain scores measured as per the (0–10) scale, where 10 is the greatest pain and zero is pain-free. The secondary outcomes were opioid consumption as oral morphine equivalents (OME) in mg in the first 24 h postoperative period and time to mobilisation.

### Data collection process

To ensure the same characteristic data was extracted from each included paper, a spreadsheet with pre-determined fields was created. Study characteristics and the primary and secondary outcome data were extracted (Tables 1 and 2). Data extraction was carried out on a 1:1 allocation basis between researchers KV and AN, who then peer-reviewed to reduce risk of omissions or mistakes. Any disagreements on data extraction were solved by discussion or by seeking the opinion of a third researcher.

### Risk of bias assessment

The included studies were appraised for bias risk using Cochrane risk of bias 2 (RoB2) tool [10]. Judgements of the RoB2 analyses were peer reviewed and any conflicts were resolved after discussion.

### Synthesis methods

To assess the primary outcome of postoperative pain scores, a meta-analysis was performed using Stata [11]. Where continuous outcomes were reported in included studies only as a median with interquartile range, the mean and standard deviation were imputed using a method based on the approach described by Hozo et al. [12]. This approach was only utilised when necessary. To synthesise postoperative analgesia consumption, the mean analgesic consumption of either fentanyl, tramadol or parenteral morphine was converted to OMEs [13, 14].

### Meta-analysis

For outcomes yielding data from three or more studies, a meta-analysis was performed using a random-effects model [15] as per Cochrane recommendation to incorporate the assumption that different studies are estimating different, yet related, treatment effects [16]. For each pairwise comparison, a standardised mean difference (SMD) (Hedges’s g) and 95% confidence interval were calculated. These were selected due to discrepancies between the discrete numeric rating scale (NRS) and the continuous visual analogue scale (VAS). The SMD was converted to the raw mean difference (RMD) by multiplying the pooled standard deviation by Hedges’s g [17, 18]. Data for opioid consumption was pooled and a mean difference (MD) with 95% confidence interval calculated. 95% prediction intervals were calculated for each outcome.

RMD values were interpreted considering the minimal clinically important difference (MCID) [19]. For postoperative pain scores at any time point, a difference of 1.5 (at rest) and 1.8 (dynamic) on a 0–10 scale were deemed the MCID, and 25 mg of oral morphine was the MCID for postoperative analgesic consumption in 24 h postoperatively [20]. The MCIDs from the Laigaard et al. [20] review were employed as they reflected the population included in this review, adults (>16 years old) undergoing hip fracture surgery.

Bonferroni correction was considered post-hoc to account for multiple comparisons between timepoints.

To evaluate inconsistency, I^2^ >50% was considered substantial heterogeneity [16]. To explore inconsistency and robustness of effect sizes, post-hoc analyses were carried out by analysing subgroups: (1) timing of administration of the block (pre- or post-anaesthesia) (2) low risk of bias (3) supra-inguinal approach of FICB vs. infra-inguinal or unknown (4) different volumes/doses of anaesthetic used in each group (5) spinal or general anaesthetic (6) block adjunct use (7) patient-controlled analgesia (PCA) pump use, request or unknown (for the consumption of OMEs in 24 h postoperative period outcome).

Influence analysis by means of leave-one-out analysis, and outlier analysis by excluding studies whose 95% CI was outside Hedges’s g 95% CI, were performed. Where an outcome included 10 or more studies, an assessment of small study effects was done using a funnel plot in combination with Egger’s test [21].

Strength of evidence for each outcome was determined using the Grading of Recommendations, Assessment, Development, and Evaluations (GRADE) framework and classified as very low, low, moderate or high [22, 23].

## Findings

### Study selection (results of search)

**Fig. 1:**
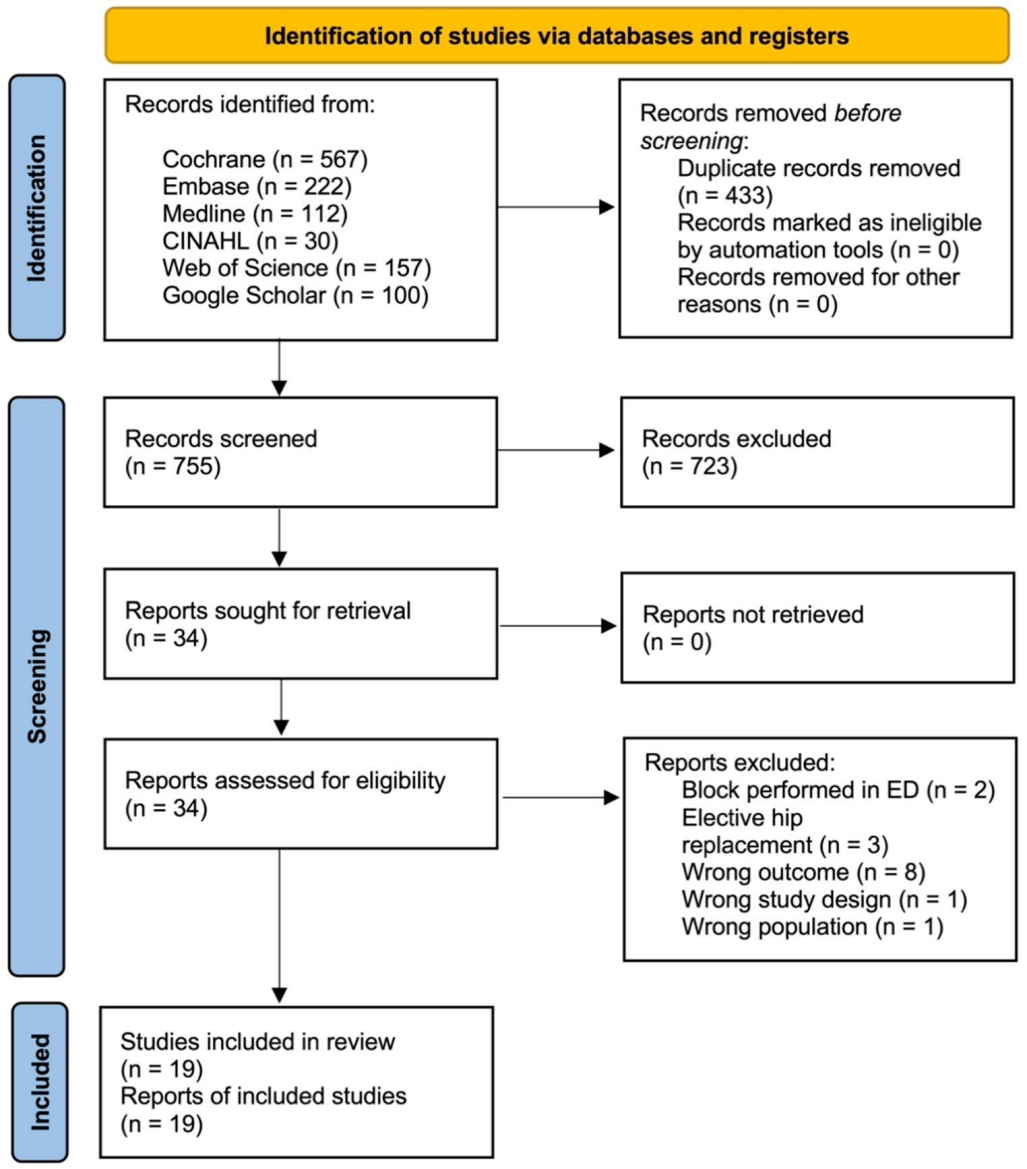
PRISMA 2020 flow diagram depicting the study selection

The search retrieved 1188 records, 755 records were screened, and 34 reports reviewed. 15 further reports were excluded, resulting in 18 studies [10–24, 26–25] initially included in this review, with one further study included from the update in April 2025 [26] (Fig.1).

### Study characteristics

A total of 19 studies (1059 patients) were included (Table 1)*.* Baseline characteristics such as age, American Society of Anaesthetists status (ASA), and sex were similar between intervention and control groups across all studies (*P* > 0.05). 16 studies compared PENG to FICB, of which eight specified using the supra-inguinal approach FICB (SIFICB). Two studies compared PENG to FNB. One study compared FICB to FNB.

16 of the included studies used the same dose of anaesthetic, either bupivacaine (six studies), ropivacaine (12 studies) or levobupivacaine (one study), in both groups (Table 1). Two studies, however, used different volumes of ropivacaine in PENG and FICB groups. Two studies reported the maximum volume of anaesthetic used in the FICB group but not in the PENG group. Yadav et al. [25] reported using 3 ml/kg of 0.5% ropivacaine. Given the maximum volume of 40 ml in their FICB group, this significantly exceeds the maximum dose. On balance, this is likely to be a reporting error.

All interventions were performed in an operating department by anaesthetists. In 15 studies, the intervention was performed before anaesthesia, and in the remaining four studies the intervention was administered after surgery. Patients received spinal anaesthesia (SA) or general anaesthesia (GA) (Table 1).

Seven studies reported that analgesia was administered via patient-controlled analgesia (PCA). The remaining studies relied on patient requests for analgesia administered by a nurse. All included studies reported data relating to our primary outcome of postoperative pain scores and 16 of these reported total postoperative analgesia consumption in 24 h. No papers reported time to mobilisation.

### Risk of bias

**Fig. 2:**
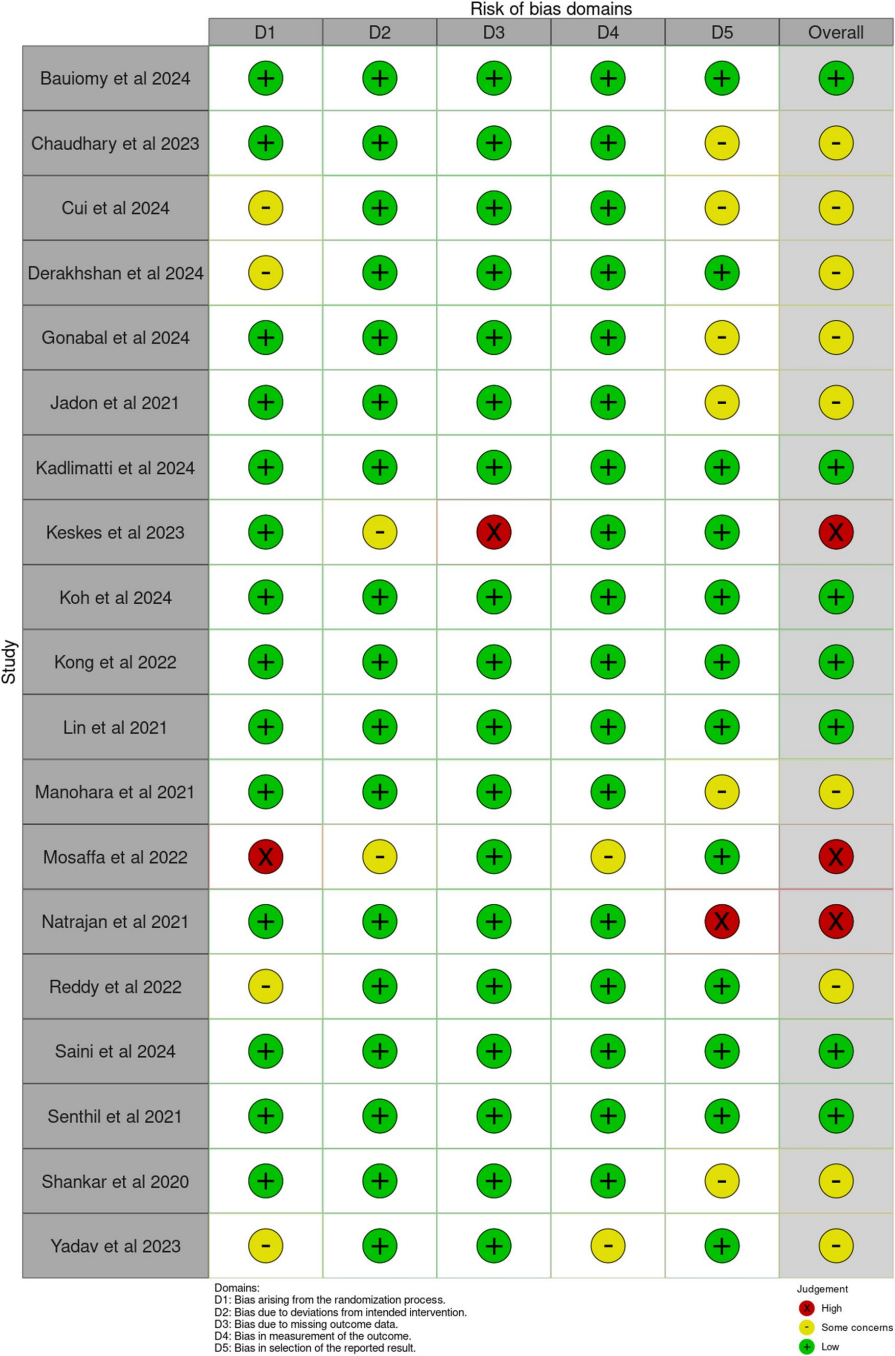
RoB2 tool outcome matrix

The focus in the RoB2 scoring was the primary outcome of pain scores (Fig. 2)*.* Seven studies [27, 28, 18–29, 26, 30] were deemed to be low risk of bias. There were some concerns for risk of bias in nine studies [11–31, 32, 24, 33, 25]. The concerns primarily arose as part of the ‘randomisation process’, ‘measurement of the outcome’ or ‘selection of the reported result’ domains. The study by Natjaran et al. [34] scored a high risk of bias overall due to concerns in the ‘selection of the reported result’ domain. This decision was reached due to published data being contradicted in the discussion and conclusion of the study. As it was not possible to blind the anaesthetist performing the nerve block, five included studies [35, 36, 24, 30, 25] detailed that the intervention was performed by an anaesthetist external to the study with a view to reduce risk of bias. It should be noted that two studies [35, 37] introduced bias in their methodology, not covered by RoB2, as a higher dose of ropivacaine was used in the FICB group than the PENG group.

**Table 1:** Study characteristics

	Study Characteristics				Intervention 1			Intervention 2		
Study (Author, Year)	Study Location	Study funder/sponsor	Fracture type (as described by study)	Timing of block placement	Group name	No. randomised to group	Drug/Dose (given to achieve intervention)	Group name	No. randomised to group	Drug/Dose (given to achieve intervention)
Bauiomy et al., 2024 [27]	Egypt	nil	hip	before SA	PENG	30	23 ml of ropivacaine 0.25% + 8 mg dexamethasone	SIFICB*	30	23 ml of ropivacaine 0.25% + 8 mg dexamethasone
Chaudhary et al., 2023 [38]	India	nil	neck of femur, intertrochanteric, & subtrochanteric fractures	before SA	PENG	32	20 mL of 0.25% bupivacaine + 4 mg dexamethasone	FNB	30	20 mL of 0.25% bupivacaine + 4 mg dexamethasone
Cui et al., 2024 [35]	China	Medical Technology Support Fund of the First People ’s Hospital of Lianyungang (XJ202208) and Lianyungang First People ’s Hospital Youth Excellent Fund (QN202108)	hip	before GA	PENG	36	20 mL of 0.3% ropivacaine	SIFICB*	36	40 mL 0.3% ropivacaine
Derakshan et al., 2024 [39]	Iran	nil	intertrochanteric	after surgery	PENG	25	20 mL of 0.25% ropivacaine	FICB*	25	20 mL of 0.25% ropivacaine
Gonobal et al., 2024 [36]	India	nil	hip	after surgery	PENG	33	30 mL of 0.375% ropivacaine	SIFICB*	33	30 mL of 0.375% ropivacaine
Jadon et al., 2021[31]	India	nil	Intracapsular, Intertrochanteric, Subtrochanteric	before SA	PENG	33	23 ml of 0.25% bupivacaine + 8 mg dexamethasone	SIFICB*	33	23 ml of 0.25% bupivacaine + 8 mg dexamethasone
Kadlimatti et al., 2024 [28]	India	nil	hip	before SA	PENG	20	25 ml of 0.25% bupivacaine	FICB*	20	25 ml of 0.25% bupivacaine
Keskes et al., 2023 [40]	Tunisia	nil	hip	before SA	PENG	46	10 ml of 0.5% bupivacaine + 10 ml of 2% lidocaine	SIFICB*	48	10 ml of 0.5% bupivacaine + 10 ml of 2% lidocaine
Koh et al., 2024 [37]	Korea	Institutional and departmental sources	hip	before SA	PENG	40	20 mL of 0.3% ropivacaine	SIFICB*	40	30 mL of ropivacaine
Kong et al., 2022 [41]	China	nil	intertrochanteric	before GA	PENG	25	30 mL of 0.375% ropivacaine	FICB*	25	30 mL of 0.375% ropivacaine
Lin et al., 2024 [29]	Australia	nil	Intracapsular Extracapsular	before anaesthesia	PENG	30	20 ml of 0.75% ropivacaine	FNB	30	20 ml of 0.75% ropivacaine
Manohara et al., 2021[32]	Singapore	Funding received from Changi General Hospital, Singapore; research grant was used for employment of a research assistant.	hip	before SA	FICB	15	0.5% ropivacaine 30 mls	FNB	15	0.5% ropivacaine 30 mls
Mosaffa et al., 2022 [42]	Iran	nil	intertrochanteric, femoral neck	before SA	PENG	30	3 mg/kg of 0.5% ropivacaine	FICB*	22	3 mg/kg of 0.5% ropivacaine 40 ml (max)
Natjaran et al., 2021 [34]	India	nil	hip	before SA	PENG	12	20 ml of 0.5% ropivacaine	FICB*	12	20 ml of 0.5% ropivacaine
Reddy et al., 2022 [24]	India	Not Reported	hip	after surgery	PENG	20	30 ml 0.25% bupivacaine + 4 mg dexamethasone	FICB*	20	30 ml 0.25% bupivacaine + 4 mg dexamethasone
Saini et al., 2024 [26]	India	nil	hip	after SA, before surgery	PENG	30	30 ml 0.2% ropivacaine with 4 mg dexamethasone	SIFICB*	30	30 ml 0.2% ropivacaine with 4 mg dexamethasone
Senthil et al., 2021 [30]	India	nil	neck of femur, intertrochanteric, subtrochanteric	after surgery	PENG	22	30 mL of 0.25% levobupivacaine + 4 mg dexamethasone	FICB*	21	30 mL of 0.25% levobupivacaine + 4 mg dexamethasone
Shankar et al., 2020 [33]	India	nil	hip	before SA	PENG	30	Ropivacaine 0.25% 25 ml	FICB*	30	Ropivacaine 0.25% 25 ml
Yadav et al., 2023 [25]	India	Not Reported	intertrochanteric, femoral neck	before SA	PENG	25	3 ml/kg 0.5% ropivacaine	FICB*	25	3 ml/kg 0.5% ropivacaine (up to 40 ml)

SA = spinal anaesthesia; GA = general anaesthesia * This review considers SIFICB and FICB to be the same intervention, SIFICB (supra-inguinal fascia iliaca compartment block) is noted where the study specified the approach, FICB (fascia-iliaca compartment block) is noted where the approach was not specified

### Primary outcomes

#### Postoperative pain scores at 6, 12 and 24 h – at rest: PENG vs. FICB

**Fig. 3:**
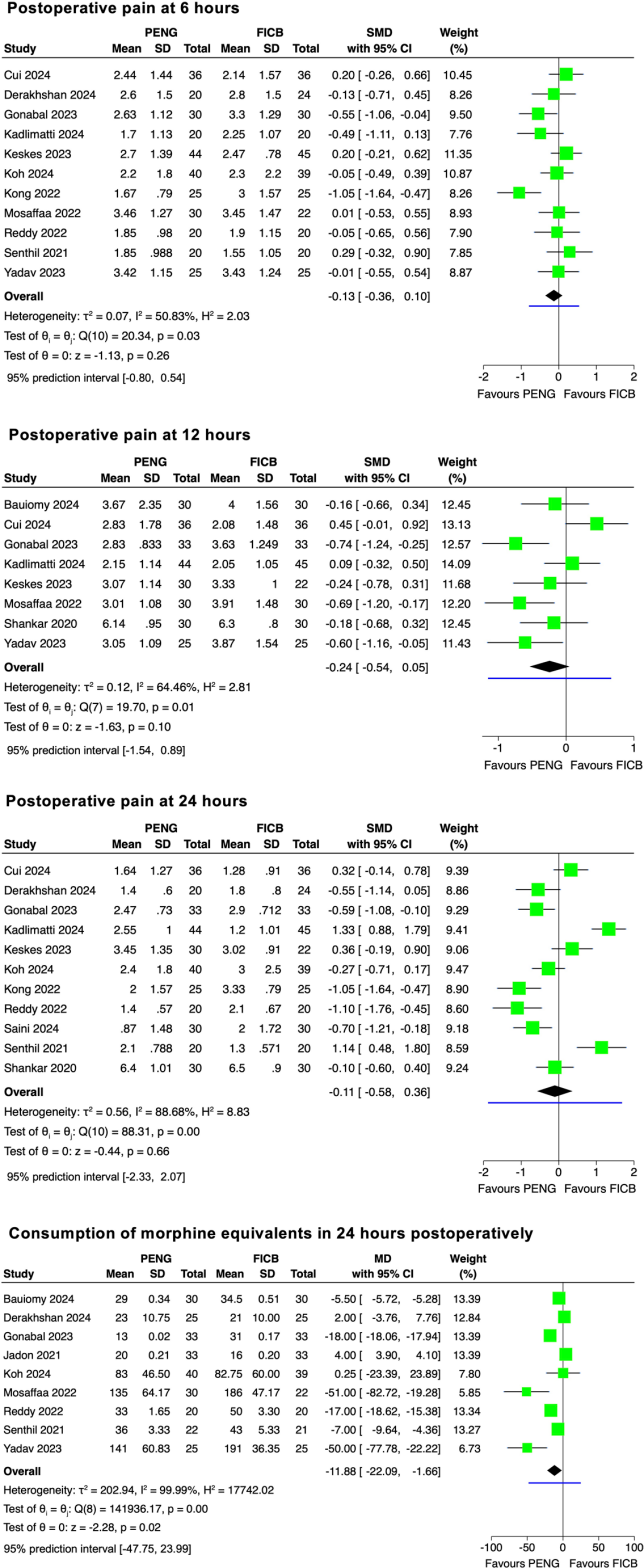
Four forest plots comparing PENG to FICB for postoperative pain scores at rest at 6, 12 and 24 h and postoperative opioid consumption in 24 h. Standardised mean difference (Hedges’s g) for postoperative pain at 6, 12 and 24 h and mean difference for opioid consumption, with 95% CI were used to calculate effect size. The blue line below the overall effect represents the 95% prediction interval

A synthesis of pain scores at rest was performed for 6, 12 and 24 h postoperatively, as these were the most reported data points across the studies (Fig. 3)*.*

11 studies, including 616 patients, reported on pain scores at 6 h postoperatively, eight studies (509 patients) reported on pain scores at 12 h, and 11 studies (652 patients) reported on pain scores at 24 h. At 6, 12 and 24 h, differences between PENG and FICB were not statistically significant. At 6 h a SMD (Hedges’s g) of −0.13 (95% CI: [−0.36, 0.10]) and RMD of −0.18 (95% CI: [−0.49, 0.14]) was found (Fig. 3). At 12 h a SMD (Hedges’s g) of −0.24 (95% CI: [−0.54, 0.05]) and RMD of −0.32 (95% CI: [−0.72, 0.07]), (Fig. 3)*.* And at 24 h and SMD (Hedges’s g) of −0.11 (95% CI: [−0.58, 0.36]), RMD of −0.14 (95% CI: [−0.72, 0.45]), (Fig. 3)*.*

None of these differences met the clinically meaningful threshold of 1.5 (on a 0–10 scale). The converted 95% prediction intervals (from Standardised to Raw MD) were − 1.10 to 0.74 for 6 h, −1.54 to 0.89 for 12 h and − 2.33 to 2.07 for 24 h postoperatively.

Due to inconsistency (I^2^ of 50.83%, 64.46%, 88.68% for 6, 12, and 24 h, respectively (Fig. 3), subgroup and sensitivity analyses were performed (see Supplementary Files 2)*.* The test of no group differences showed there was no significant difference between any of the groups at 6 and 24 h postoperatively (*P* >0.05). For the pain scores at the 12-hour outcome, there was a significant difference between groups, in different volumes of anaesthetic used (*P* = 0.00), and general vs. spinal anaesthetic use (*P* = 0.00), but only driven by one study [35].

Influence (leave-one-out) analysis for 6 and 24 h, confirmed robustness of the result upon exclusion of each study from the pooled result. Outlier analysis for 24 h [28, 30], showed a statistically significant but not clinically meaningful effect (SMD of −0.39 (95% CI: [−0.72, −0.05]), RMD of −0.48 (95% CI: [−0.89, −0.06]), *P* < 0.05) (Table 2).

**Table 2:** Table summarising the primary and secondary outcomes for each study

Study (Author, Year, Reference)	Intervention 1	Intervention 2	Time points reported	Postoperative pain score unit of measurement	Postoperative analgesia consumption reported unit of measurement
Bauiomy et al., 2024 [27]	PENG	SIFICB*	PACU, 2 h, 4 h,12, and 24 h postoperatively	NRS score	Number of tramadol doses (1 = 50 mg Tramadol)
Chaudhary et al., 2023 [38]	PENG	FNB	4, 6, 8, and 12 h postoperatively	VAS score	Unable to compare, reported duration reported rather than consumption
Cui et al., 2024 [35]	PENG	SIFICB*	1, 6, 12, 24 h postoperatively	VAS score	Amount of postoperative analgesia doses (in Parecoxib sodium 40 mg/dose and sufentanil 1.5 µg/L)
Derakshan et al., 2024 [39]	PENG	FICB*	After surgery, immediately after the block, 2,6,18,24 h post op	VAS score	Mean total morphine consumption (mg) within the first 24 h post-surgery
Gonobal et al., 2024 [36]	PENG	SIFICB*	0 (baseline score), 1, 2, 4, 6, 12, 24 h in the postoperative period	VAS score	Total opioid consumption in oral morphine equivalents (in mg)
Jadon et al., 2021 [31]	PENG	SIFICB*	postoperatively at 4, 6, 8, 12 and 24 h	NRS score	Number of doses of rescue analgesia post operatively (1 = 50 mg Tramadol)
Kadlimatti et al., 2024 [28]	PENG	FICB*	0,2,4,6,8,10,12,16,20,24 h postoperatively	VAS score	Total doses of consumption of tramadol in mg/kg IV
Keskes et al.,2023 [40]	PENG	SIFICB*	3, 6, 12, and 24 h postoperatively	VAS score	Only mean morphine consumption (mg) in 2 h post op
Koh et al., 2024 [37]	PENG	SIFICB*	Baseline score, after block, during positioning, at 6, 24 and 48 h	NRS score	Given as morphine equivalents of IV morphine in 24 h
Kong et al., 2022 [41]	PENG	FICB*	6 h, 24 h postoperatively	VAS score	Total dose of fentanyl consumed in mcg in 24 h
Lin et al., 2024 [29]	PENG	FNB	maximum score within 24 h, max score 24–48 h	NRS score	Total amount of opiate consumed post operatively day 0 (in morphine equivalents in mg)
Manohara et al., 2021 [32]	FICB	FNB	before block, 30 min post block, 24 h postoperatively	NRS score	Oxynorm consumption in the first 24 h
Mosaffa et al., 2022 [42]	PENG	FICB*	15 min, 6 h, 12 h post block	VAS score	Total dose of morphine consumed within 24 h (in mg)
Natjaran et al., 2021 [34]	PENG	FICB*	30 min, 1 h, 4 h, 6 h, 12 h, 24 h	NRS score	Total number of rescue analgesia doses *(*IV paracetamol)
Reddy et al., 2022 [24]	PENG	FICB*	2,6,10,14,18,24 h postoperatively	VAS score	Total fentanyl consumption in 24 h (in mcg)
Saini et al., 2024 [26]	PENG	SIFICB*	24 h postoperatively	VAS score	Unit not given; opioids named (multiple) but only one measure
Senthil et al., 2021 [30]	PENG	FICB*	2,6,10,14,18,24 h postoperatively	VAS score	Total fentanyl consumption in 24 h (in mcg)
Shankar et al., 2020 [33]	PENG	FICB*	0, 30 min, 1, 4, 12, 24 h postoperatively	VAS score	Total consumption of tramadol in 24 h (1 mg/kg)
Yadav et al., 2023 [25]	PENG	FICB*	15 min, 6 h, 12 h post block	VAS score	Total dose of parenteral morphine consumed within 24 h (in mg)

*This review considers SIFICB and FICB to be the same intervention, SIFICB (supra-inguinal fascia iliaca compartment block) is noted where the study specified the approach, FICB (fascia-iliaca compartment block) is noted where the approach was not specified

Influence analysis for 12 h showed that omission of one study [35] resulted in a statistically significant, yet still not yielding a clinically meaningful, result (SMD of −0.34 (95% CI: [−0.59, −0.09], RMD of −0.45 (95% CI: [−0.79, −0.12]), *P* = 0.007), favouring PENG over FICB.

The funnel plots for 6 and 24 h (Supplementary Files 2) showed slight asymmetry; however, Egger’s test was not statistically significant (*P* = 0.25, *P* = 0.50, respectively), hence there was no significant evidence of risk of small study effects.

Strength of evidence for postoperative pain scores at 6 and 24 h was low and for 12 h very low (Table 3)*.*

#### Postoperative pain scores at 6, 12 and 24 h – at rest: PENG vs. FNB

Only Chaudhary et al. [38] compared PENG to FNB at 6 and 12 h. They reported that PENG lowered postoperative pain scores at 6 h with a significant difference compared to FNB (*P* = 0.004). At 12 h, there was no significant difference (*P* = 0.05).

#### Postoperative pain scores at 6, 12 and 24 h – at rest: FICB vs. FNB

Manohara et al. [32] found no significant difference between FICB and FNB (*P* = 0.334) at 24 h. No other studies compared these two blocks.

#### Postoperative pain scores at 6 and 24 h - dynamic

Four studies (235 patients) compared dynamic pain scores between PENG and FICB at 6 and 24 h. No study reported dynamic pain scores at 12 h postoperatively. A non-significant result of SMD of −0.30 (95% CI [−1.00, 0.39]), RMD of −0.91 (95% CI [−3.02, 1.18]) and SMD of [−0.28 (95% CI [−0.83, 0.27]), RMD of −0.52 (95% CI [−1.55, 0.50]) was found for dynamic pain scores at 6 and 24 h postoperatively, respectively. Subgroup analysis for dynamic pain scores at 6 and 24 h showed a significant difference between the general and spinal anaesthetic groups (*P* = 0.00), due to one study [41]. Subgroup analysis for block adjunct use showed a significant difference (*P* = 0.01), due to Senthil et al. only [30] for the 6 h outcome. Influence analysis confirmed robustness for 6 h, but for 24 h, exclusion of Senthil et al. [30] drove a significant yet imprecise result (SMD: −0.51 (95% CI [−0.96, −0.06]), RMD: −0.95 (95% CI [−1.80, −0.11]), *p* = 0.027). GRADE scores for 6 and 24 h were therefore very low.

No studies compared PENG and FNB postoperative pain scores on movement.

Manohara et al. [32] found no significant difference between FICB and FNB at 24 h on movement (*P* = 0.937).

**Table 3:** Evidence profile for adult patients with a fractured proximal femur, undergoing surgery, receiving PENG or FICB

Outcome	Risk of bias	Inconsistency	Indirectness	Imprecision	Publication bias	Number of patients (studies)	Overall effect (95% CI)	Quality of evidence (GRADE)
**Postoperative pain at 6 h**	serious^a^	serious^b^	not serious	not serious	not detected	616 (11)	RMD **0.18 SD lower** (0.49 lower to 0.14 higher)	⨁⨁◯◯ Low^a, b^
**Postoperative pain at 12 h**	not serious^c^	serious^d^	serious^e^	serious^f^	not detected	509 (8)	RMD **0.32 SD lower** (0.72 lower to 0.07 higher)	⨁◯◯◯ Very low^c, d,e, f^
**Postoperative pain at 24 h**	not serious^g^	very serious^h^	not serious	not serious	not detected	652 (11)	RMD **0.14 SD lower** (0.72 lower to 0.45 higher)	⨁⨁◯◯ Low^g, h^
**Dynamic postoperative pain at 6 h**	not serious^i^	very serious^j^	not serious	serious^k^	not detected	235 (4)	RMD **0.91 lower** (3.02 lower to 1.18 higher)	⨁◯◯◯ Very low^i, j,k^
**Dynamic postoperative pain at 24 h**	not serious^l^	very serious^m^	not serious	serious^n^	not detected	235 (4)	RMD **0.52 lower** (1.55 lower to 0.50 higher)	⨁◯◯◯ Very low^l, m,n^
**Consumption of oral morphine equivalents at 24 h**	not serious^o^	very serious^p^	not serious	serious^q^	not detected	506 (9)	MD **11.88 SD lower** (22.09 lower to 1.66 lower)	⨁◯◯◯ Very low^o, p,q^
**Time to mobilisation** ^r^	**N/A**	**N/A**	**N/A**	**N/A**	**N/A**	**N/A**	**N/A**	**N/A**
a. The test of group differences was not statistically significant (*P* > 0.05); however, the majority of studies contributing to the overall effect size (7 out of 11 studies) were rated moderate to high risk of bias. b. Test for inconsistency (I^2^ of 50.83%). c. The test of group differences was not statistically significant (*P* > 0.05); however, the majority of studies contributing to the overall effect size (2 out of 8 studies) were rated moderate to high risk of bias. The subgroup analysis for low RoB showed I^2^ = 0.00%; however, these were only 2 studies. d. Test for inconsistency (I^2^ of 64.46%). e. Difference in interventions arose due to Cui et al. using a greater dose of ropivacaine in the FICB group, with the test of group differences *P* < 0.05. f. Upon exclusion of one study, the overall effect did not remain robust, resulting in a statistically significant effect size. g. 5 out of 11 studies were low risk of bias, and the test of group differences was not statistically significant. h. Very high heterogeneity (I^2^ of 88.68%) and wide prediction interval. i. Majority of studies (3 out of 4) were rated low risk of bias. j. Very high heterogeneity (I^2^ of 85.57%) and wide prediction intervals. k. Lower bound of the 95% CI (−3.02 to 1.18) exceeds the minimal clinically important difference of 1.8 on the 0–10 pain scale. A wide confidence interval suggests appreciable benefit and appreciable harm. l. Majority of studies (3 out of 4) were rated low risk of bias. m. High heterogeneity (I^2^ of 77.50%) and wide prediction interval. n. Upon exclusion of one study, the overall effect did not remain robust, resulting in a statistically significant effect size. o. The test of group differences was not statistically significant (*P* > 0.05); however, the majority of studies contributing to the overall effect size (3 out of 9 studies) were rated moderate to high risk of bias. The subgroup analysis for low RoB showed I^2^ = 0.00%; however, these were only 3 studies. p. Very high heterogeneity (I^2^ of 99.99%) and very wide prediction interval. q. Prediction interval crosses null and confidence interval. r. No studies included in this study reported data for this outcome.

**Table 4:** Summary of findings of pooled data for adult patients with a fractured proximal femur, undergoing surgery, receiving PENG or FICB

Outcome	PENG Pooled Mean (SD)	FICB Pooled Mean (SD)	Number of patients (studies)	RMD (or MD) (95% CI)	Quality of evidence (GRADE)	Comments
**Postoperative pain at 6 h**	2.47 (1.41)	2.60 (1.52)	616 (11)	−0.18 (−0.49 to 0.14)	Low	Moderate inconsistency (I^2^ of 50.83%)
**Postoperative pain at 12 h**	3.27 (1.76)	3.52 (1.81)	509 (8)	−0.32 (−0.72 to 0.07)	Very low	High inconsistency (I^2^ of 64.46%). Difference in interventions arose due to one study using a greater dose of ropivacaine in the FICB group.
**Postoperative pain at 24 h**	2.50 (1.85)	2.56 (1.92)	652 (11)	−0.14 (−0.72 to 0.45)	Low	High inconsistency (I^2^ of 88.68%)
**Dynamic postoperative pain at 6 h**	2.92 (1.70)	3.56 (4.18)	235 (4)	− 0.91 (−3.02 to 1.18)	Very low	Test for inconsistency (I^2^ of 85.57%). Lower and upper bound of the 95% CI both exceed the minimal clinically important difference.
**Dynamic postoperative pain at 24 h**	3.26 (2.06)	3.89 (2.50)	235 (4)	−0.52 (−1.55 to 0.50)	Very low	Test for inconsistency (I^2^ of 77.50%) and wide prediction interval. Upon exclusion of one study the overall effect did not remain robust, resulting in a statistically significant effect size.
**Consumption of oral morphine equivalents at 24 h**	57.68 (57.99)	69.02 (68.36)	506 (9)	−16.33 (−22.15 to −10.51)	Very low	High inconsistency (I^2^ of 99.99%). Prediction interval crosses null and confidence interval.
**Time to mobilisation**	**N/A**	**N/A**	**N/A**	**N/A**	**N/A**	No studies included in this study reported data for this outcome

RMD: Raw mean difference; MD: Mean difference

### Secondary outcomes

#### Consumption of oral morphine equivalents in 24 h postoperatively – PENG vs. FICB

Nine studies (506 patients) were included in the meta-analysis. Total opioid consumption was lower in the PENG than in the FICB group (MD of − 11.88 mg (95% CI: [−22.09, −1.66])) (Fig. 3). The 95% prediction interval was − 47.75 to 23.99 (Fig. 3). Although a statistically significant result, the MD for oral morphine fell short of the MCID (25 mg). The test of group differences was statistically significant for PCA use, patient request or unknown (*P* = 0.00) (see Supplementary Files 2)*.* Influence (leave-one-out) analysis showed that upon exclusion of two studies in turn [42, 25], the overall effect size became statistically insignificant (*P* > 0.05). Outlier analysis confirmed robustness of the overall result (see Supplementary Files 2). GRADE certainty in evidence was very low (Table 3)*.*

#### Consumption of oral morphine equivalents in 24 h postoperatively – PENG vs. FNB

Lin et al. [29] was the only study comparing total opioid consumption between PENG and FNB and found no significant difference between the groups (*P* = 0.85) .

#### Consumption of oral morphine equivalents in 24 h postoperatively – FICB vs. FNB

Manohara et al. [32] found no significant difference between FICB and FNB (*P* = 0.87).

#### Time to mobilisation following surgery

No studies reported time to mobilisation.

## Discussion

### Overall findings

Although a significant reduction of postoperative pain scores was found at rest at 6 h in PENG compared to FNB, and analgesic consumption was significantly lowered in PENG compared to FICB, neither of these results were clinically meaningful. Time to mobilisation was not reported in any other reviews, highlighting an evidence gap that could be especially meaningful within this field. The GRADE strength of evidence rating was very low to low. Overall, this review indicates that the three peripheral blocks provide minimally variable pain relief in patients with hip fractures undergoing surgical repair.

### Implications for research

A review comparing PENG to FICB [43] found the former may improve patient analgesia, however, the scope of this review was inclusive of all hip operations. Another review comparing PENG to other regional blocks found PENG was superior to FICB and FNB regarding postoperative motor weakness; they did not evaluate postoperative pain scores [44]. Two previous reviews, including all three blocks in the emergency department, found PENG may be superior to FNB and FICB for pain relief [5], but their certainty was low [4]. The current lack of evidence relating to time to mobilisation could be addressed by primary researchers going forward. Addressing this evidence deficit using standardised milestones of mobilisation such as time to first stand or walk may provide greater insight into analgesic superiority.

### Implications for practice

Considering the outcomes, availability of evidence and strength as per GRADE, PENG may be superior in improving postoperative analgesia in hip fracture patients in some domains reported on in trials, but no clinically important differences were found overall. In clinical practice, this should be carefully considered with the MCID, as none of the results met this threshold.

### Strengths and limitations

This review has some notable strengths. The comprehensive, repeated search allowed inclusion of 19 studies, with 15 included in meta-analyses, resulting in an adequate pooled sample size. Due to the number of studies included, analysis of pain scores at several postoperative timepoints was possible.

This review also has some limitations. As smaller studies were included, this could have led to reduced accuracy when determining the estimated treatment effect. Difference between postoperative pain scales used in the studies could have contributed to risk of bias. VAS and NRS scales are subjective, and understanding may vary between patients. To account for this, SMD was used and converted to RMD for interpretation in the clinical setting. To explore heterogeneity, subgroup and sensitivity analyses were performed; however, these only partially resolved heterogeneity in some groups. The 95% prediction intervals for all outcomes suggest future studies will unlikely find a statistically significant result. Given the sample sizes of the studies included in the review, we recognise the potential for Type II error, and future studies should consider including larger sample sizes to investigate these between timepoints.

The decision to include only peer-reviewed, full-length published RTs sought to ensure the included data was of suitable quality and subject to academic rigour; however, this may have resulted in an element of publication bias. This review was limited by the sole use of aggregate data, which prevented any individual-level characteristic analyses, further exploration of heterogeneity and robustness of treatment effects, as well as identifying confounders and publication bias.

Limiting the inclusion date to 2014–2025 could have potentially excluded relevant studies that included FNB. This time limit was selected to review contemporary papers to align with current practice standards. In a future review, this time limit could be modified to allow a possible increase in the number of studies comparing FNB to FICB.

Lin et al. [29] presented data as a maximum postoperative pain score (NRS), rather than a mean value, at four hours on day zero and day one after surgery. It was not specified at which hour on day one the pain scores were assessed. As only two studies [38, 29] compared PENG to FNB, meta-analyses of postoperative pain scores and analgesic consumption in patients receiving a PENG block compared to FNB could not be performed.

Finally, this review did not include a control arm, which limits findings to patients undergoing hip fracture repair who are able to receive PNB. Results do not generalise to those patients receiving systemic multimodal analgesia, or neuraxial opioid analgesia, which are all alternative pain management strategies for this surgical population.

## Conclusion

In conclusion, this review yielded clinically non-meaningful postoperative analgesic benefits in PENG compared to FICB or FNB. Also, although a statistically significant result for reduction in opioid consumption in patients receiving PENG compared to FICB, this outcome too was not clinically meaningful. As such, we suggest anaesthetists would be justified in continuing to administer the regional block in which they feel most competent, given the limitations of the review and the low quality of the current evidence available.

## Supplementary Information

Below is the link to the electronic supplementary material.

Supplementary Material 1

Supplementary Material 2

## Data Availability

Data available from the authors upon reasonable request.
