# Supplementary material for: The postoperative analgesic efficacy of three peripheral nerve blocks in hip fracture surgery: a systematic review and meta-analysis of randomised trials
Source: Arch Orthop Trauma Surg. 2026 Jan 6;146(1):24. doi: 10.1007/s00402-025-06139-6 (PMC12775034; doi:10.1007/s00402-025-06139-6)
Supplement: Supplementary file 1 — Supplementary Material 1 [file 402_2025_6139_MOESM1_ESM.pdf]

Search strategy examples

MEDLINE

#	Searches	Results
1	Hip Fractures/ or hip fracture*.mp.	*31071*
2	exp Femoral Fractures/ or femoral fracture*.mp. or femur fracture*.mp.	*53444*
3	exp Femoral Neck Fractures/ or neck of femur fracture*.mp.	*10792*
4	femoral neck fracture*.mp.	*12889*
5	intracapsular.mp.	*3888*
6	extracapsular.mp	*7614*
7	trochanteric.mp.	*5122*
8	intertrochanteric.mp.	*4699*
9	5 or 6 or 7 or 8	*19757*
10	fracture*.mp.	393380
11	9 and 10	*7764*
12	1 or 2 or 3 or 4 or 11	*63575*
13	exp Arthroplasty, Replacement, Hip/ or hip surger*.mp.	*41288*
14	hemi arthroplasty.mp. or exp Hemiarthroplasty/	*1828*
15	hip replacement*.mp.	*15310*
16	hip arthroplasty.mp.	*33988*
17	13 or 14 or 15 or 16	*60860*
18	12 or 17	*116508*
19	pericapsular nerve group block*.mp.	*191*
20	PENG*.mp.	*4757*
21	PENG block*.mp.	*272*
22	19 or 20 or 21	*4799*
23	fascia iliaca block*.mp.	*261*
24	fascia iliaca compartment block*.mp.	*322*
25	FICB*.mp.	*195*
26	23 or 24 or 25	*548*
27	infra-inguinal.mp.	*320*
28	supra-inguinal.mp.	*91*
29	27 or 28	*390*
30	26 and 29	*53*
31	SIFICB*.mp.	*41*
32	IIFICB*.mp.	*0*
33	26 or 30 or 31 or 32	*550*
34	femoral block*.mp.	*193*
35	femoral nerve block*.mp.	*1304*
36	FNB*.mp.	*2290*
37	34 or 35 or 36	*3364*
38	22 and 33 and 37	*11*
39	22 and 33	*67*
40	22 and 37	*26*
41	33 and 37	*77*

42	38 or 39 or 40 or 41	*148*
43	18 and 42	*122*
44	limit 43 to yr="2014 -Current"	*112*

CINAHL

#	Query	Limiters/Expanders	Last Run Via	Results
S43	S18 AND S42	Limiters - Publication Date: 20140101-20241231<br>Expanders - Apply equivalent subjects<br>Search modes - Find all my search terms	Interface - EBSCOhost Research Databases<br>Search Screen - Advanced Search<br>Database - CINAHL	30
S42	S38 OR S39 OR S40 OR S41	Expanders - Apply equivalent subjects<br>Search modes - Find all my search terms	Interface - EBSCOhost Research Databases<br>Search Screen - Advanced Search<br>Database - CINAHL	46
S41	S33 AND S37	Expanders - Apply equivalent subjects<br>Search modes - Find all my search terms	Interface - EBSCOhost Research Databases<br>Search Screen - Advanced Search<br>Database - CINAHL	34
S40	S22 AND S37	Expanders - Apply equivalent subjects<br>Search modes - Find all my search terms	Interface - EBSCOhost Research Databases<br>Search Screen - Advanced Search<br>Database - CINAHL	8
S39	S22 AND S33	Expanders - Apply equivalent subjects<br>Search modes - Find all my search terms	Interface - EBSCOhost Research Databases<br>Search Screen - Advanced Search<br>Database - CINAHL	10
S38	S22 AND S33 AND S37	Expanders - Apply equivalent subjects<br>Search modes - Find all my search terms	Interface - EBSCOhost Research Databases<br>Search Screen - Advanced Search<br>Database - CINAHL	3
S37	S34 OR S35 OR S36	Expanders - Apply equivalent subjects<br>Search modes - Find all my search terms	Interface - EBSCOhost Research Databases<br>Search Screen - Advanced	884

			Search<br>Database - CINAHL	
S36	"FNB*"	Expanders - Apply equivalent subjects<br>Search modes - Find all my search terms	Interface - EBSCOhost Research Databases<br>Search Screen - Advanced Search<br>Database - CINAHL	386
S35	"femoral nerve block*"	Expanders - Apply equivalent subjects<br>Search modes - Find all my search terms	Interface - EBSCOhost Research Databases<br>Search Screen - Advanced Search<br>Database - CINAHL	592
S34	"femoral block*"	Expanders - Apply equivalent subjects<br>Search modes - Find all my search terms	Interface - EBSCOhost Research Databases<br>Search Screen - Advanced Search<br>Database - CINAHL	71
S33	S26 OR S30 OR S31 OR S32	Expanders - Apply equivalent subjects<br>Search modes - Find all my search terms	Interface - EBSCOhost Research Databases<br>Search Screen - Advanced Search<br>Database - CINAHL	263
S32	"IIFICB*"	Expanders - Apply equivalent subjects<br>Search modes - Find all my search terms	Interface - EBSCOhost Research Databases<br>Search Screen - Advanced Search<br>Database - CINAHL	0
S31	"SIFICB*"	Expanders - Apply equivalent subjects<br>Search modes - Find all my search terms	Interface - EBSCOhost Research Databases<br>Search Screen - Advanced Search<br>Database - CINAHL	0
S30	S26 AND S29	Expanders - Apply equivalent subjects<br>Search modes - Find all my search terms	Interface - EBSCOhost Research Databases<br>Search Screen - Advanced Search<br>Database - CINAHL	23
S29	S27 OR S28	Expanders - Apply equivalent subjects<br>Search modes - Find all my search terms	Interface - EBSCOhost Research Databases<br>Search Screen - Advanced Search<br>Database - CINAHL	76

S28	"supra-inguinal*"	Expanders - Apply equivalent subjects<br>Search modes - Find all my search terms	Interface - EBSCOhost Research Databases<br>Search Screen - Advanced Search<br>Database - CINAHL	26
S27	"infra-inguinal*"	Expanders - Apply equivalent subjects<br>Search modes - Find all my search terms	Interface - EBSCOhost Research Databases<br>Search Screen - Advanced Search<br>Database - CINAHL	55
S26	S23 OR S24 OR S25	Expanders - Apply equivalent subjects<br>Search modes - Find all my search terms	Interface - EBSCOhost Research Databases<br>Search Screen - Advanced Search<br>Database - CINAHL	263
S25	"FICB*"	Expanders - Apply equivalent subjects<br>Search modes - Find all my search terms	Interface - EBSCOhost Research Databases<br>Search Screen - Advanced Search<br>Database - CINAHL	74
S24	"fascia iliaca compartment block*"	Expanders - Apply equivalent subjects<br>Search modes - Find all my search terms	Interface - EBSCOhost Research Databases<br>Search Screen - Advanced Search<br>Database - CINAHL	142
S23	"fascia iliaca block*"	Expanders - Apply equivalent subjects<br>Search modes - Find all my search terms	Interface - EBSCOhost Research Databases<br>Search Screen - Advanced Search<br>Database - CINAHL	129
S22	S19 OR S20 OR S21	Expanders - Apply equivalent subjects<br>Search modes - Find all my search terms	Interface - EBSCOhost Research Databases<br>Search Screen - Advanced Search<br>Database - CINAHL	808
S21	"PENG block*"	Expanders - Apply equivalent subjects<br>Search modes - Find all my search terms	Interface - EBSCOhost Research Databases<br>Search Screen - Advanced Search<br>Database - CINAHL	98
S20	"PENG*"	Expanders - Apply equivalent subjects	Interface - EBSCOhost Research Databases	790

		Search modes - Find all my search terms	Search Screen - Advanced Search<br>Database - CINAHL	
S19	"pericapsular nerve group block*"	Expanders - Apply equivalent subjects<br>Search modes - Find all my search terms	Interface - EBSCOhost Research Databases<br>Search Screen - Advanced Search<br>Database - CINAHL	41
S18	S12 OR S17	Expanders - Apply equivalent subjects<br>Search modes - Find all my search terms	Interface - EBSCOhost Research Databases<br>Search Screen - Advanced Search<br>Database - CINAHL	56,109
S17	S13 OR S14 OR S15 OR S16	Expanders - Apply equivalent subjects<br>Search modes - Find all my search terms	Interface - EBSCOhost Research Databases<br>Search Screen - Advanced Search<br>Database - CINAHL	42,078
S16	"hip arthroplasty"	Expanders - Apply equivalent subjects<br>Search modes - Find all my search terms	Interface - EBSCOhost Research Databases<br>Search Screen - Advanced Search<br>Database - CINAHL	22,312
S15	"hip replacement*"	Expanders - Apply equivalent subjects<br>Search modes - Find all my search terms	Interface - EBSCOhost Research Databases<br>Search Screen - Advanced Search<br>Database - CINAHL	20,082
S14	(MH "Hemiarthroplasty") OR "hemiarthroplasty"	Expanders - Apply equivalent subjects<br>Search modes - Find all my search terms	Interface - EBSCOhost Research Databases<br>Search Screen - Advanced Search<br>Database - CINAHL	1,834
S13	(MH "Arthroplasty, Replacement, Hip") OR "arthroplasty, replacement, hip" OR hip surger*	Expanders - Apply equivalent subjects<br>Search modes - Find all my search terms	Interface - EBSCOhost Research Databases<br>Search Screen - Advanced Search<br>Database - CINAHL	38,704
S12	S1 OR S2 OR S3 OR S4 OR S11	Expanders - Apply equivalent subjects<br>Search modes - Find all my search terms	Interface - EBSCOhost Research Databases<br>Search Screen - Advanced	23,535

			Search<br>Database - CINAHL	
S11	S9 AND S10	Expanders - Apply equivalent subjects<br>Search modes - Find all my search terms	Interface - EBSCOhost Research Databases<br>Search Screen - Advanced Search<br>Database - CINAHL	2,499
S10	"fracture*"	Expanders - Apply equivalent subjects<br>Search modes - Find all my search terms	Interface - EBSCOhost Research Databases<br>Search Screen - Advanced Search<br>Database - CINAHL	107,083
S9	S5 OR S6 OR S7 OR S8	Expanders - Apply equivalent subjects<br>Search modes - Find all my search terms	Interface - EBSCOhost Research Databases<br>Search Screen - Advanced Search<br>Database - CINAHL	4,333
S8	"intertrochanteric*"	Expanders - Apply equivalent subjects<br>Search modes - Find all my search terms	Interface - EBSCOhost Research Databases<br>Search Screen - Advanced Search<br>Database - CINAHL	1,401
S7	"trochanteric*"	Expanders - Apply equivalent subjects<br>Search modes - Find all my search terms	Interface - EBSCOhost Research Databases<br>Search Screen - Advanced Search<br>Database - CINAHL	1,609
S6	"extracapsular*"	Expanders - Apply equivalent subjects<br>Search modes - Find all my search terms	Interface - EBSCOhost Research Databases<br>Search Screen - Advanced Search<br>Database - CINAHL	978
S5	"intracapsular*"	Expanders - Apply equivalent subjects<br>Search modes - Find all my search terms	Interface - EBSCOhost Research Databases<br>Search Screen - Advanced Search<br>Database - CINAHL	642
S4	"femoral neck fracture*"	Expanders - Apply equivalent subjects<br>Search modes - Find all my search terms	Interface - EBSCOhost Research Databases<br>Search Screen - Advanced Search<br>Database - CINAHL	2,450
S3	(MH "Femoral Neck Fractures+") OR "neck of femur fracture*"	Expanders - Apply equivalent subjects<br>Search modes - Find all my search terms	Interface - EBSCOhost Research Databases<br>Search Screen - Advanced Search<br>Database - CINAHL	574
S2	(MH "Femoral Fractures+") OR "femoral fracture*" OR "femur fracture*"	Expanders - Apply equivalent subjects<br>Search modes - Find all my search terms	Interface - EBSCOhost Research Databases<br>Search Screen - Advanced Search<br>Database - CINAHL	19,707
S1	(MH "Hip Fractures") OR "hip fracture*"	Expanders - Apply equivalent subjects<br>Search modes - Find all my search terms	Interface - EBSCOhost Research Databases<br>Search Screen - Advanced Search<br>Database - CINAHL	15,961

Embase

#	Searches	Results
1	hip fracture/ or hip fracture*.mp.	47061
2	femur fracture/ or exp proximal femur fracture/	50341
3	femoral fracture*.mp.	11219
4	exp femoral neck fracture/ or femoral neck fracture*.mp. or exp femur neck fracture/	18085
5	neck of femur fracture*.mp.	822
6	intracapsular.mp.	5605
7	extracapsular.mp.	11807
8	exp femur trochanteric fracture/ or trochanteric.mp.	7719
9	exp femur intertrochanteric fracture/ or intertrochanteric.mp.	7635
10	6 or 7 or 8 or 9	30182
11	fracture*.mp.	561459
12	10 and 11	11905
13	1 or 2 or 3 or 4 or 5 or 12	92921
14	hip arthroplasty.mp. or exp hip arthroplasty/	61088
15	hip surgery/ or hip surger*.mp.	10836
16	exp hip replacement/ or hip replacement*.mp.	38206
17	exp hemiarthroplasty/ or exp replacement arthroplasty/ or exp hip hemiarthroplasty/	76411
18	hemi arthroplasty.mp.	284
19	14 or 15 or 16 or 17 or 18	128916
20	13 or 19	207058
21	pericapsular nerve group block*.mp.	314
22	PENG*.mp.	6399
23	PENG block*.mp.	373
24	21 or 22 or 23	6464
25	fascia iliaca block*.mp.	513
26	fascia iliaca compartment block*.mp.	530
27	FICB*.mp.	360
28	25 or 26 or 27	986
29	infra-inguinal.mp.	510
30	supra-inguinal.mp.	169
31	29 or 30	642
32	28 and 31	86
33	SIFICB*.mp.	4
34	IIFICB*.mp.	1
35	28 or 32 or 33 or 34	988
36	femoral block*.mp.	427
37	femoral nerve block*.mp.	2340
38	FNB*.mp.	4016
39	36 or 37 or 38	6042
40	24 and 35 and 39	31
41	24 and 35	113
42	24 and 39	62

43	35 and 39	182
44	40 or 41 or 42 or 43	295
45	20 and 44	250
46	limit 45 to yr="2014 -Current"	222

Web of Science

#	Searches	Results
1	TS=("hip fracture*" OR "femoral fracture*" OR "femoral neck fracture*" OR "neck of femur fracture*" OR "femur fracture*")	53790
2	TS=((intracapsular OR extracapsular OR trochanteric OR intertrochanteric) and fracture*)	7486
3	TS=("hip surger*" OR "hip arthroplasty" OR "hip replacement*" OR "hemi arthroplasty" OR "hemi-arthroplasty")	56625
4	#1 OR #2 OR #3	107572
5	TS=("pericapsular nerve group block*" OR PENG* OR "PENG block*")	22272
6	TS=("fascia iliaca block*" OR "fascia iliaca compartment block*" OR "FICB*")	624
7	TS=("infra-inguinal" OR "supra-inguinal")	379
8	#6 AND #7	54
9	TS=(SIFICB*)	4
10	TS=(IIFICB*)	0
11	#10 OR #9 OR #8 OR #6	626
12	TS=("femoral block*" OR "femoral nerve block*" OR "FNB*")	4104
13	#5 AND #11	84
14	#5 AND #12	41
15	#11 AND #12	120
16	#5 AND #11 AND #12	17
17	#16 OR #15 OR #14 OR #13	211
18	#17 AND #4	171
19	19: #18	171
20	#18 and 2025 or 2024 or 2023 or 2022 or 2021 or 2025 or 2024 or 2023 or 2022 or 2021 or 2020 or 2019 or 2018 or 2017 or 2016 or 2015 or 2014 (Publication Years)	157

Cochrane

#	Search	Results
#1	MeSH descriptor: [Hip Fractures] explode all trees	2520
#2	(hip fracture*):ti,ab,kw	8025
#3	MeSH descriptor: [Femoral Fractures] explode all trees	2927
#4	(femoral fracture*):ti,ab,kw	5047
#5	MeSH descriptor: [Femoral Neck Fractures] explode all trees	620
#6	(femur fracture*):ti,ab,kw	3310
#7	(neck of femur fracture*):ti,ab,kw	1313
#8	(intracapsular):ti,ab,kw	418

#9	(extracapsular):ti,ab,kw	808
#10	(trochanteric):ti,ab,kw	624
#11	(intertrochanteric):ti,ab,kw	828
#12	#8 or #9 or # 9 or #11	545216
#13	(fracture*):ti,ab,kw	31408
#14	#12 and #13	8947
#15	#1 or #2 or #3 or #4 or #5 or #7 or #14	15488
#16	MeSH descriptor: [Arthroplasty, Replacement, Hip] explode all trees	2780
#17	(hip surger*):ti,ab,kw	10322
#18	MeSH descriptor: [Hemiarthroplasty] explode all trees	124
#19	(hemiarthroplasty or "hemi arthroplasty"):ti,ab,kw	608
#20	(hip replacement*):ti,ab,kw	6911
#21	(hip arthroplasty):ti,ab,kw	6962
#22	#16 or #17 or #18 or #19 or #20 or #21	14378
#23	#15 or #22	25712
#24	(pericapsular nerve group block*):ti,ab,kw	496
#25	(PENG*):ti,ab,kw	647
#26	(PENG block*):ti,ab,kw	495
#27	#24 or #25 or #26	739
#28	(fascia iliaca block*):ti,ab,kw	816
#29	(fascia iliaca compartment block*):ti,ab,kw	501
#30	(FICB*):ti,ab,kw	319
#31	#28 or #29 or #30	843
#32	(infra-inguinal):ti,ab,kw	63
#33	(supra-inguinal):ti,ab,kw	106
#34	#32 or #33	162
#35	#31 and #34	103
#36	(SIFICB*):ti,ab,kw	9
#37	(IIFICB*):ti,ab,kw	1
#38	#31 or #35 or #36 or #37	844
#39	(femoral block*):ti,ab,kw	3214
#40	(femoral nerve block*):ti,ab,kw	2537
#41	(FNB*):ti,ab,kw	773
#42	#39 or #40 or #41	3582
#43	#27 and #38	178
#44	#27 and #42	270
#45	#38 and #42	371
#46	#27 and #38 and #42	90
#47	#43 or #44 or #45 or #46	639
#48	#47 and #23	576
#49	#48 with Cochrane Library publication date Between Jan 2014 and May 2025	567

Google Scholar Search:
PENG, FICB, SIFICB, SFIB, FNB, "fascia-iliaca", fracture
100 results were scanned on April 2025
