# Supplementary material for: The postoperative analgesic efficacy of three peripheral nerve blocks in hip fracture surgery: a systematic review and meta-analysis of randomised trials
Source: Arch Orthop Trauma Surg. 2026 Jan 6;146(1):24. doi: 10.1007/s00402-025-06139-6 (PMC12775034; doi:10.1007/s00402-025-06139-6)
Supplement: Supplementary file 2 — Supplementary Material 2 [file 402_2025_6139_MOESM2_ESM.pdf]

The Postoperative Analgesic Efficacy of Three Peripheral Nerve Blocks in Hip Fracture Surgery: A Systematic Review and Meta-Analysis of Randomised Trials

Analysis report

Table of contents

Pain scores at 6 hours postoperatively 2
Subgroup analysis 2
Sensitivity (Leave-one-out) 8
Funnel plot 8
Pain scores at 12 hours postoperatively 9
Subgroup analysis 9
Sensitivity (Leave-one-out) 15
Pain scores at 24 hours postoperatively 16
Subgroup analysis 16
Sensitivity (Leave-one-out / outlier) 22
Funnel plot 23
Dynamic pain scores at 6 hours postoperatively 24
Forest plot 24
Subgroup analysis 24
Sensitivity (Leave-one-out) 29
Dynamic pain scores at 24 hours postoperatively 30
Forest plot 30
Subgroup analysis 30
Sensitivity (Leave-one-out) 35
Consumption of OMEs in 24 hours postoperatively 36
Subgroup analysis 36
Sensitivity (Leave-one-out / outlier) 43
Table of Drug Conversions to Oral Morphine Equivalents (OMEs) 44

Pain scores at 6 hours postoperatively – subgroup analyses

Pain at 6 hours postoperatively - timing of administration of the block (pre- or post-anaesthesia)

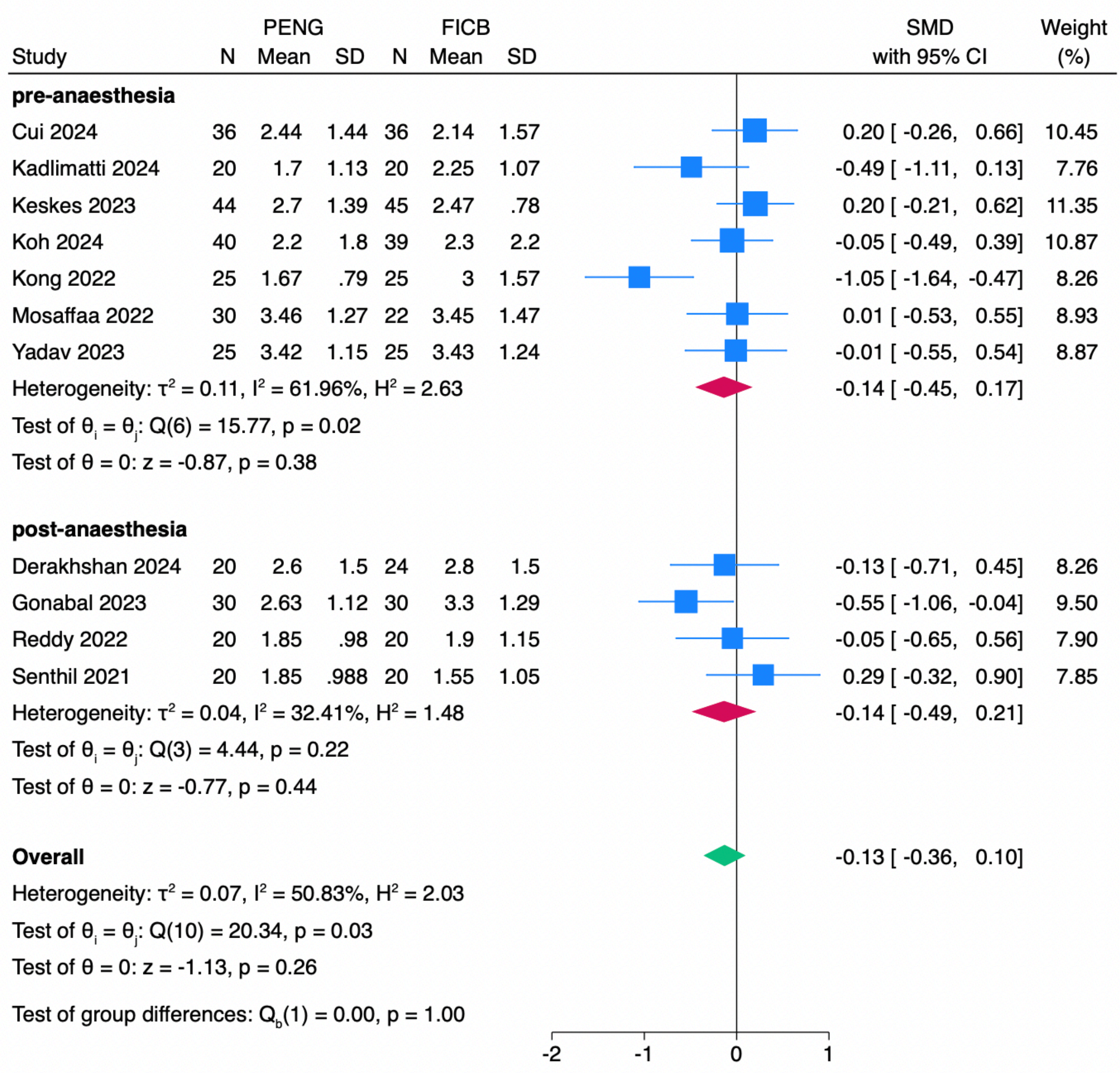

Random-effects DerSimonian–Laird model

Pain at 6 hours postoperatively - low risk of bias

Study	PENG N	PENG Mean	PENG SD	FICB N	FICB Mean	FICB SD	SMD with 95% CI	Weight (%)
**Low RoB**								
Kadlimatti 2024	20	1.7	1.13	20	2.25	1.07	-0.49 [-1.11, 0.13]	7.76
Koh 2024	40	2.2	1.8	39	2.3	2.2	-0.05 [-0.49, 0.39]	10.87
Kong 2022	25	1.67	.79	25	3	1.57	-1.05 [-1.64, -0.47]	8.26
Senthil 2021	20	1.85	.988	20	1.55	1.05	0.29 [-0.32, 0.90]	7.85
Heterogeneity: $\tau^2 = 0.23$, $I^2 = 74.12\%$, $H^2 = 3.86$							-0.32 [-0.87, 0.23]	
Test of $\theta_i = \theta_j$: Q(3) = 11.59, p = 0.01								
Test of $\theta = 0$: z = -1.14, p = 0.25								
**Moderate or high**								
Cui 2024	36	2.44	1.44	36	2.14	1.57	0.20 [-0.26, 0.66]	10.45
Derakhshan 2024	20	2.6	1.5	24	2.8	1.5	-0.13 [-0.71, 0.45]	8.26
Gonabal 2023	30	2.63	1.12	30	3.3	1.29	-0.55 [-1.06, -0.04]	9.50
Keskes 2023	44	2.7	1.39	45	2.47	.78	0.20 [-0.21, 0.62]	11.35
Mosaffaa 2022	30	3.46	1.27	22	3.45	1.47	0.01 [-0.53, 0.55]	8.93
Reddy 2022	20	1.85	.98	20	1.9	1.15	-0.05 [-0.65, 0.56]	7.90
Yadav 2023	25	3.42	1.15	25	3.43	1.24	-0.01 [-0.55, 0.54]	8.87
Heterogeneity: $\tau^2 = 0.00$, $I^2 = 4.20\%$, $H^2 = 1.04$							-0.02 [-0.22, 0.18]	
Test of $\theta_i = \theta_j$: Q(6) = 6.26, p = 0.39								
Test of $\theta = 0$: z = -0.20, p = 0.84								
**Overall**							-0.13 [-0.36, 0.10]	
Heterogeneity: $\tau^2 = 0.07$, $I^2 = 50.83\%$, $H^2 = 2.03$								
Test of $\theta_i = \theta_j$: Q(10) = 20.34, p = 0.03								
Test of $\theta = 0$: z = -1.13, p = 0.26								
Test of group differences: $Q_b(1) = 1.02$, p = 0.31								

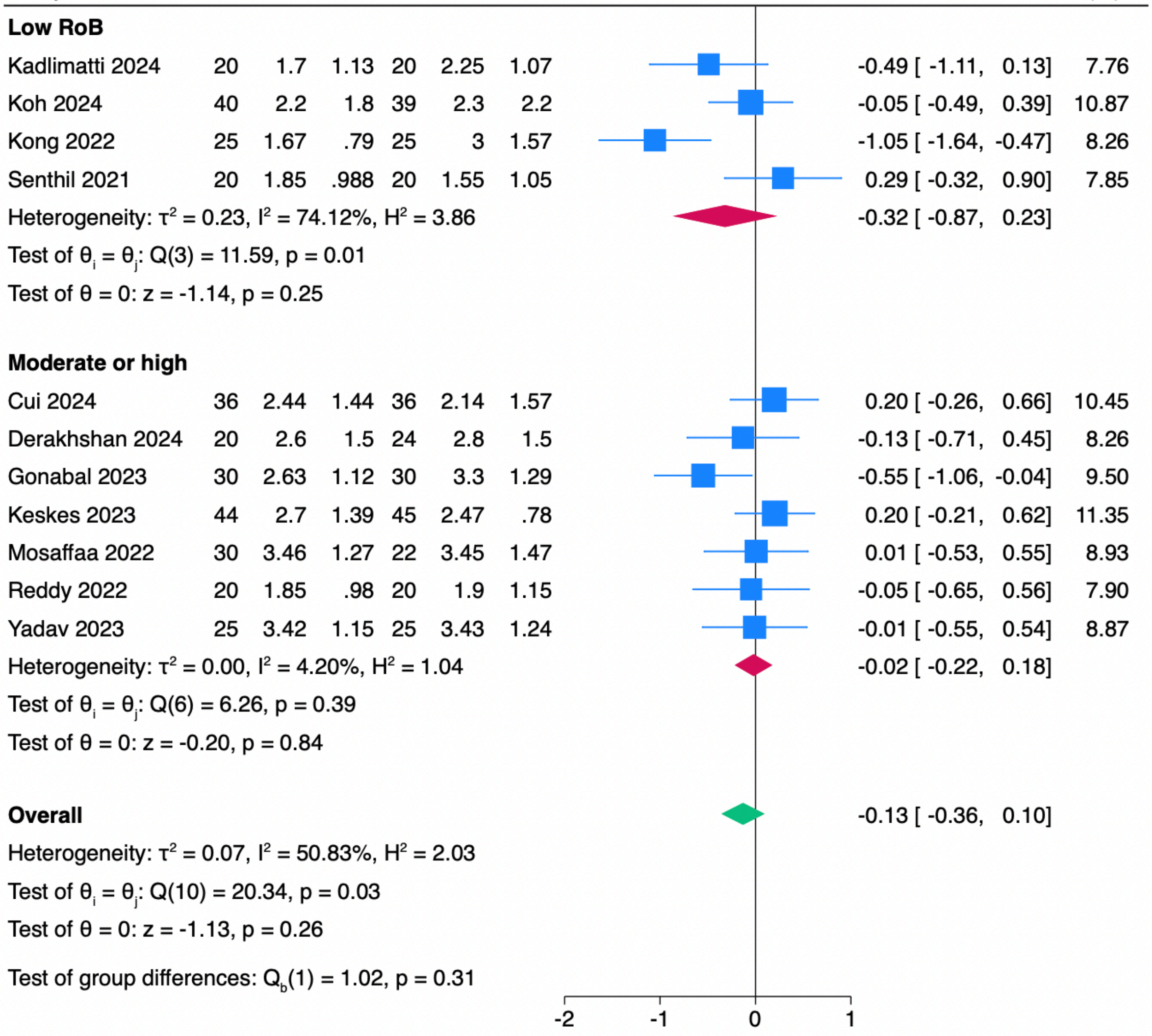

Random-effects DerSimonian–Laird model

Pain at 6 hours postoperatively - supra-inguinal approach of FICB vs infra-inguinal or unknown

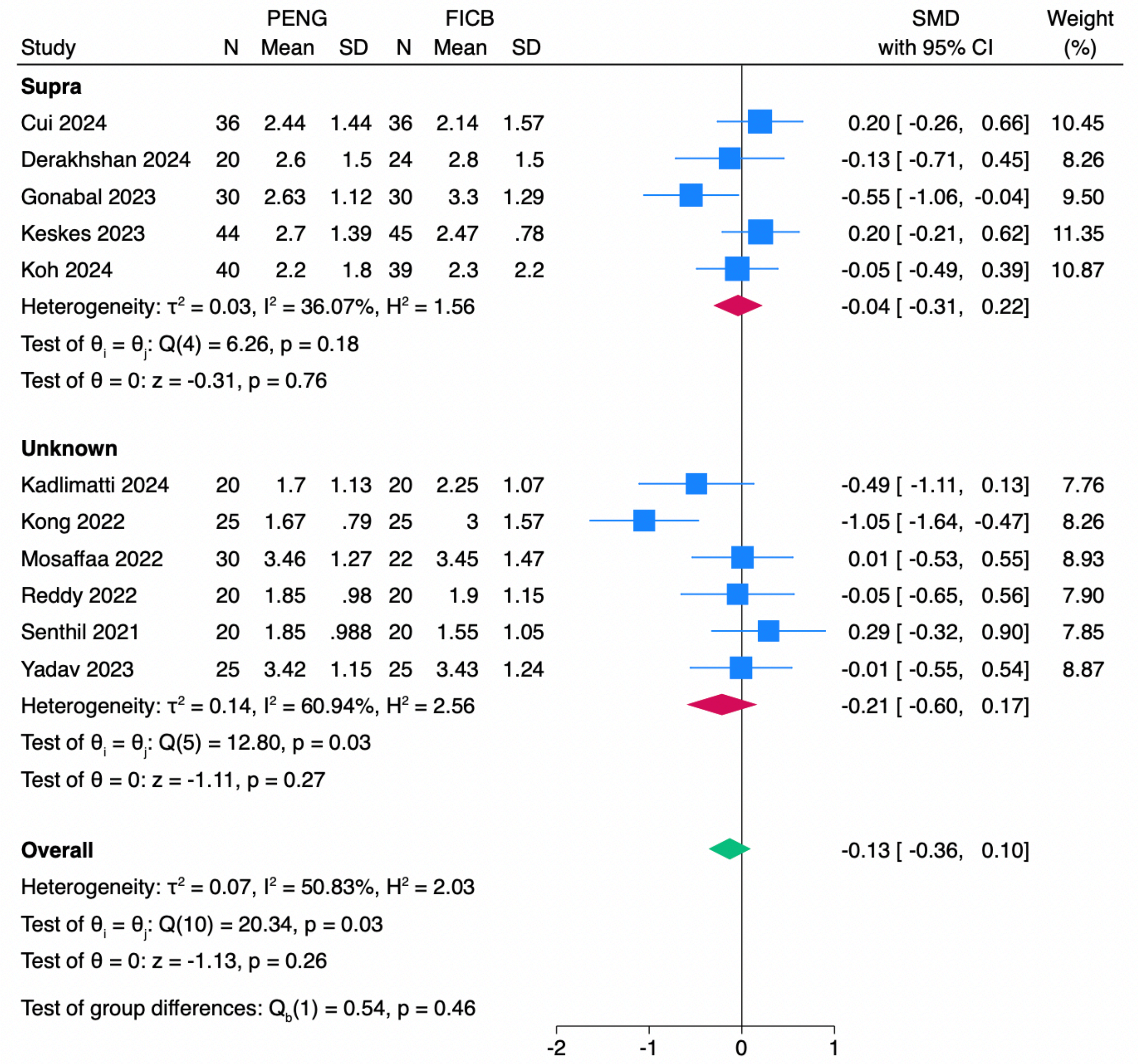

Random-effects DerSimonian–Laird model

Pain at 6 hours postoperatively - different volumes/doses of anaesthetic used in each group

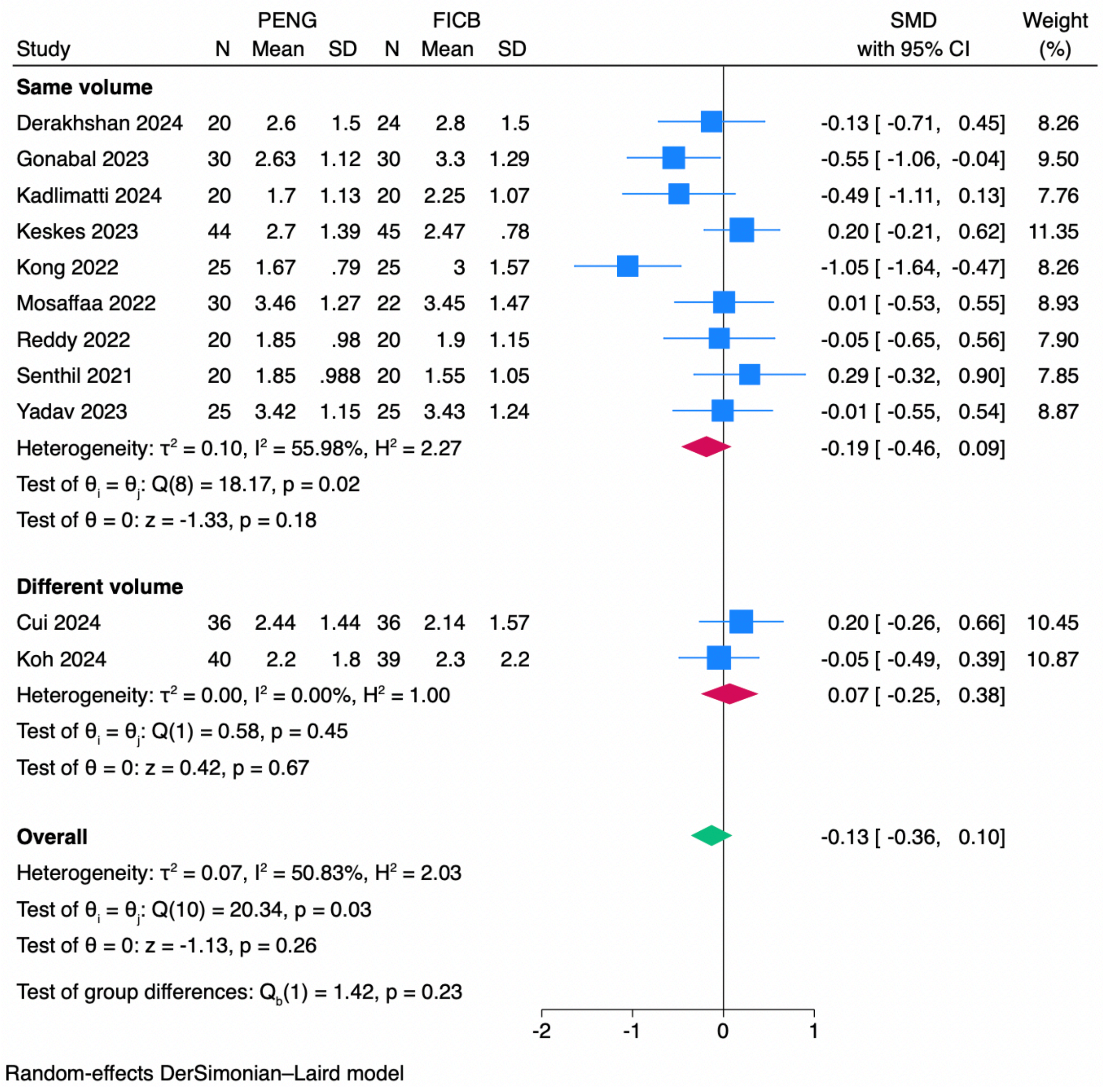

Pain at 6 hours postoperatively - spinal or general anaesthetic

Study	PENG N	PENG Mean	PENG SD	FICB N	FICB Mean	FICB SD	SMD with 95% CI	Weight (%)
**SA**								
Derakhshan 2024	20	2.6	1.5	24	2.8	1.5	-0.13 [-0.71, 0.45]	8.26
Gonabal 2023	30	2.63	1.12	30	3.3	1.29	-0.55 [-1.06, -0.04]	9.50
Kadlimatti 2024	20	1.7	1.13	20	2.25	1.07	-0.49 [-1.11, 0.13]	7.76
Keskes 2023	44	2.7	1.39	45	2.47	.78	0.20 [-0.21, 0.62]	11.35
Koh 2024	40	2.2	1.8	39	2.3	2.2	-0.05 [-0.49, 0.39]	10.87
Mosaffaa 2022	30	3.46	1.27	22	3.45	1.47	0.01 [-0.53, 0.55]	8.93
Reddy 2022	20	1.85	.98	20	1.9	1.15	-0.05 [-0.65, 0.56]	7.90
Senthil 2021	20	1.85	.988	20	1.55	1.05	0.29 [-0.32, 0.90]	7.85
Yadav 2023	25	3.42	1.15	25	3.43	1.24	-0.01 [-0.55, 0.54]	8.87
Heterogeneity: $\tau^2 = 0.00$, $I^2 = 4.11\%$, $H^2 = 1.04$							-0.07 [-0.25, 0.11]	
Test of $\theta_i = \theta_j$: $Q(8) = 8.34$, $p = 0.40$								
Test of $\theta = 0$: $z = -0.75$, $p = 0.45$								
**GA**								
Cui 2024	36	2.44	1.44	36	2.14	1.57	0.20 [-0.26, 0.66]	10.45
Kong 2022	25	1.67	.79	25	3	1.57	-1.05 [-1.64, -0.47]	8.26
Heterogeneity: $\tau^2 = 0.71$, $I^2 = 90.84\%$, $H^2 = 10.91$							-0.41 [-1.64, 0.81]	
Test of $\theta_i = \theta_j$: $Q(1) = 10.91$, $p = 0.00$								
Test of $\theta = 0$: $z = -0.66$, $p = 0.51$								
**Overall**							-0.13 [-0.36, 0.10]	
Heterogeneity: $\tau^2 = 0.07$, $I^2 = 50.83\%$, $H^2 = 2.03$								
Test of $\theta_i = \theta_j$: $Q(10) = 20.34$, $p = 0.03$								
Test of $\theta = 0$: $z = -1.13$, $p = 0.26$								
Test of group differences: $Q_b(1) = 0.30$, $p = 0.58$								

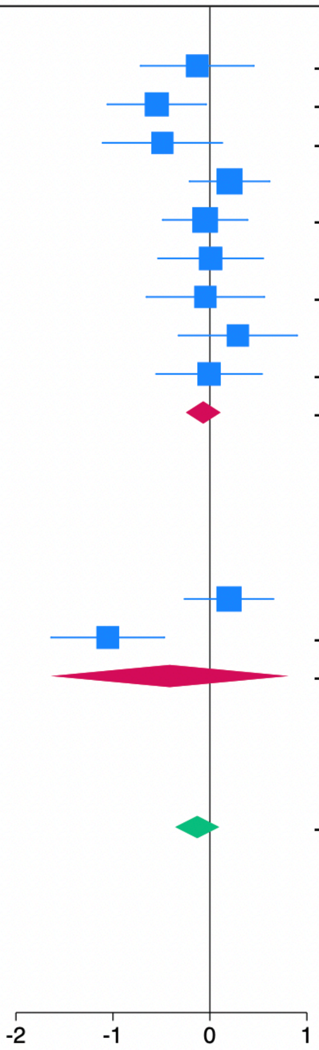

Random-effects DerSimonian–Laird model

Pain at 6 hours postoperatively - block adjunct use

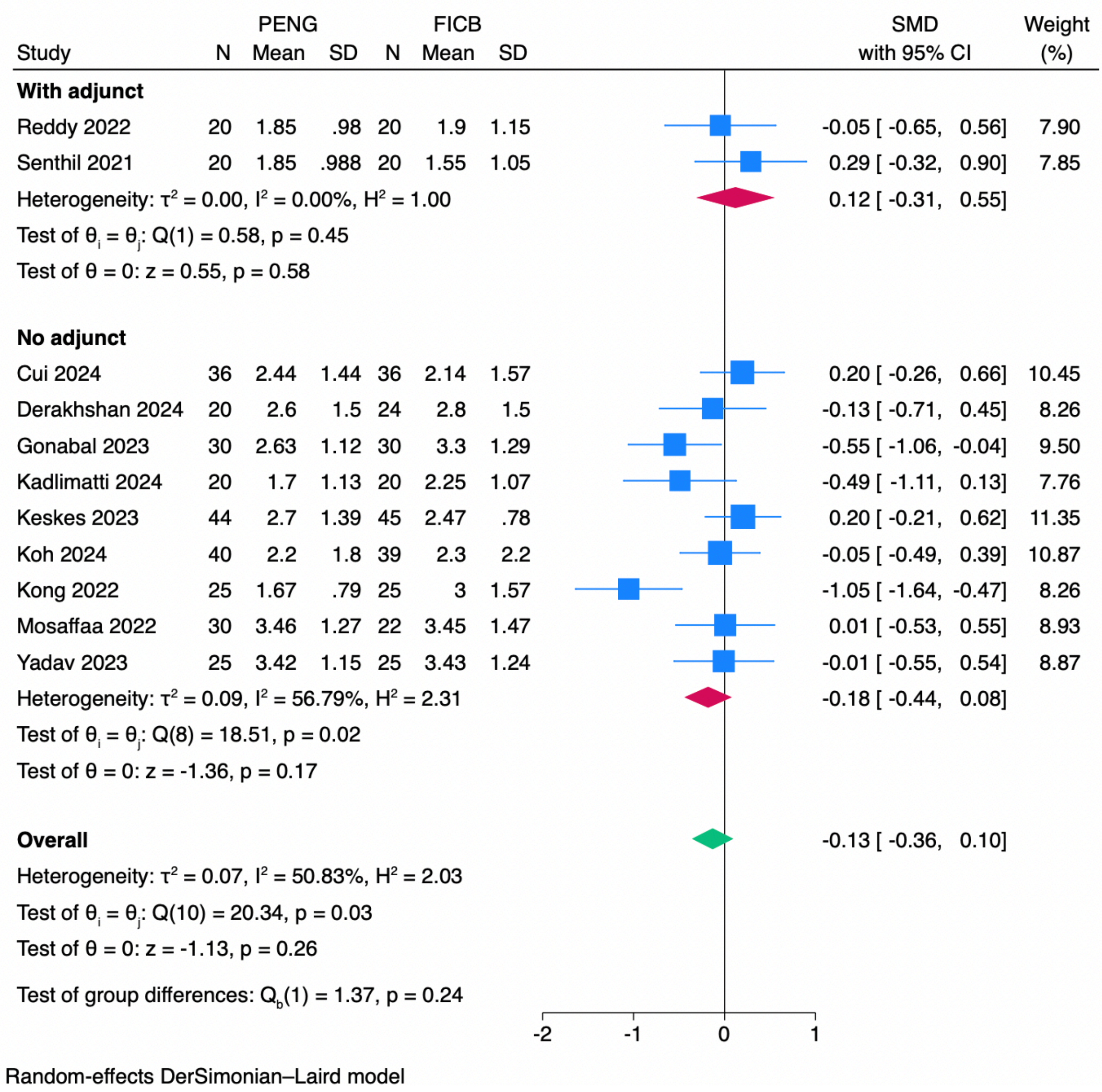

Pain at 6 hours postoperatively - leave-one-out analysis

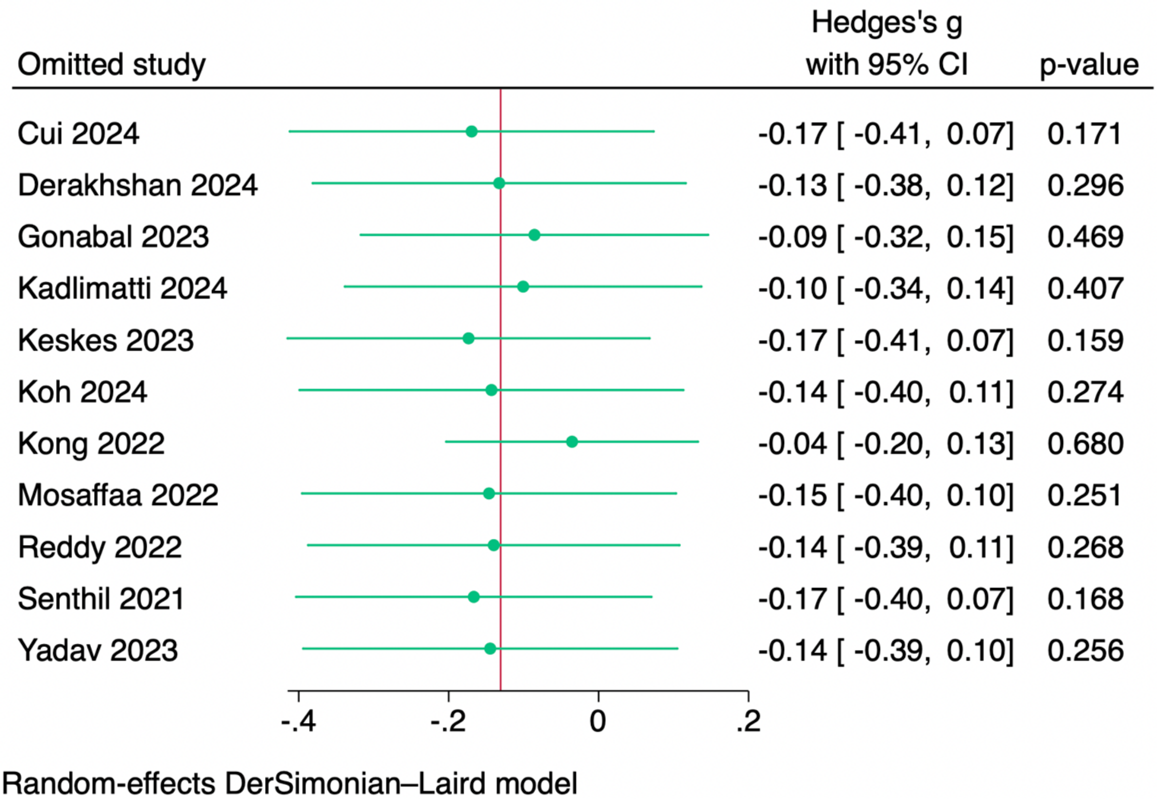

Pain at 6 hours postoperatively – funnel plot

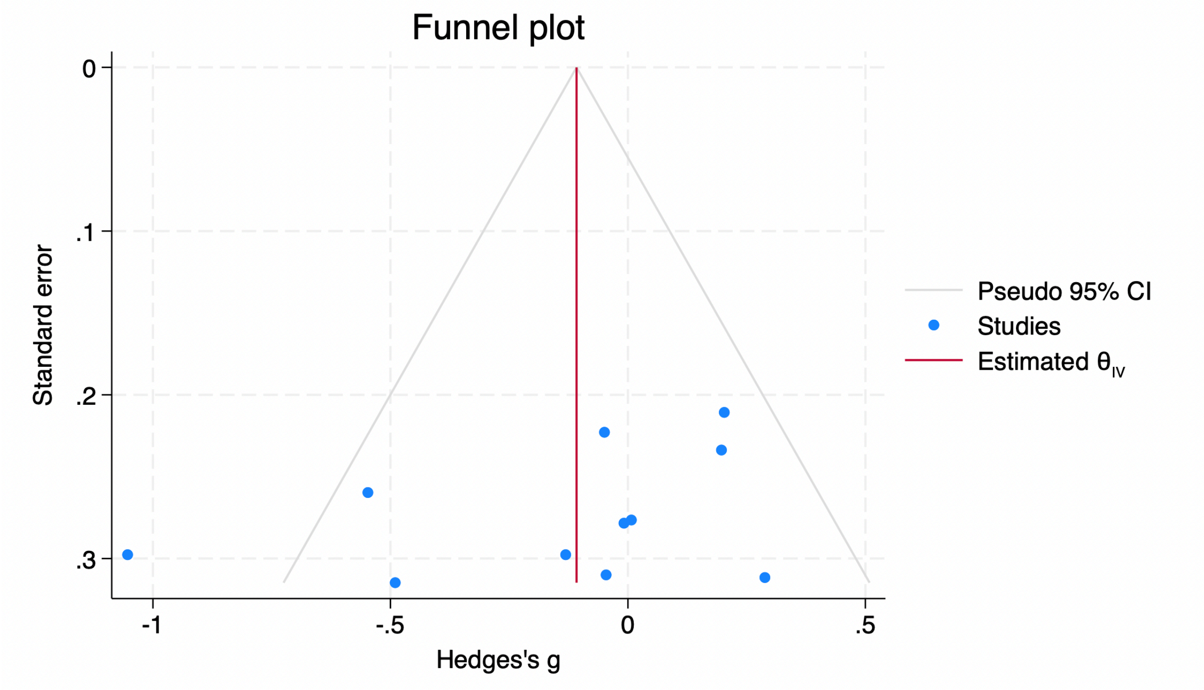

Pain scores at 12 hours postoperatively – subgroup analyses

Pain at 12 hours postoperatively - timing of administration of the block (pre- or post-anaesthesia)

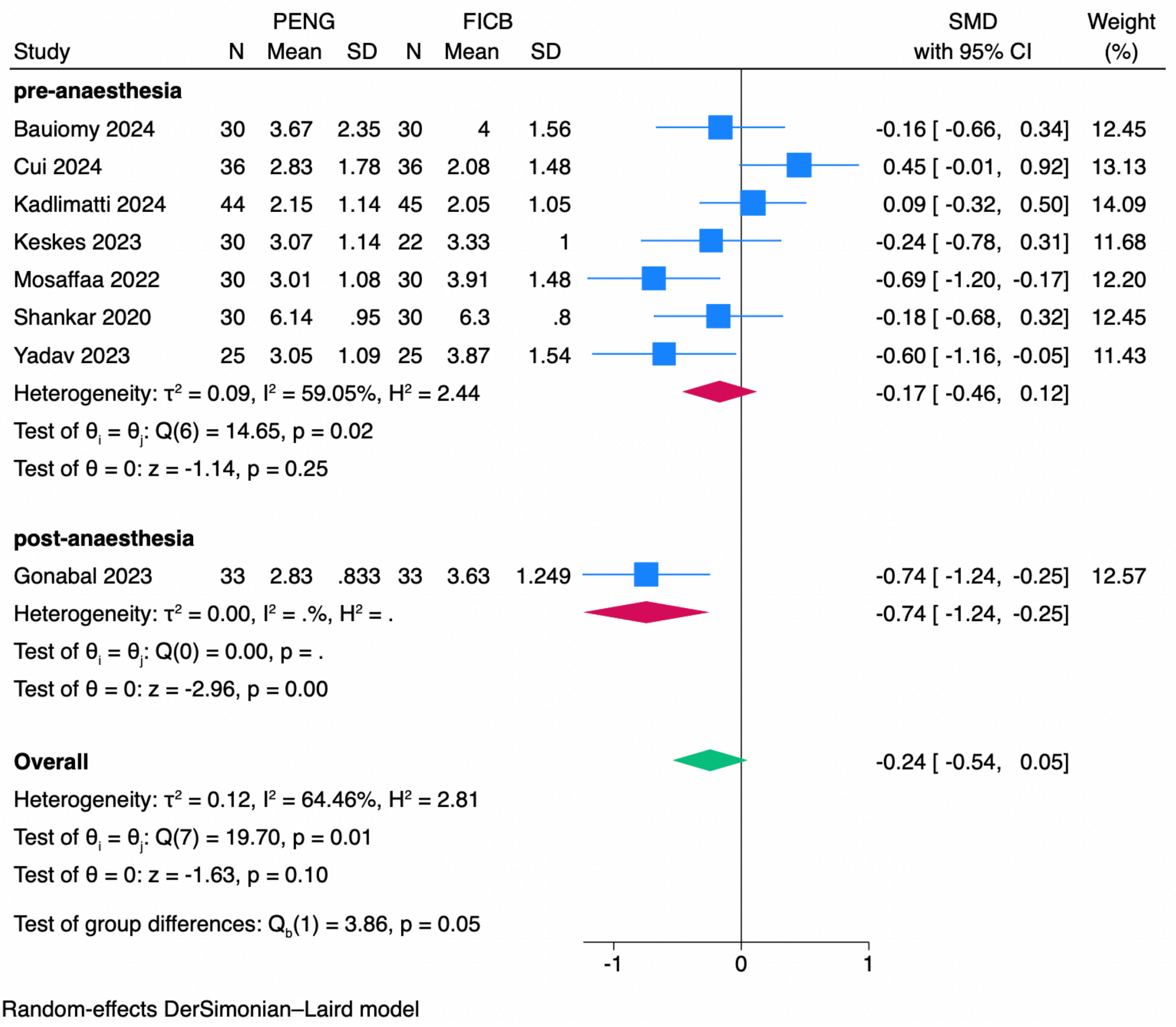

Pain at 12 hours postoperatively - low risk of bias

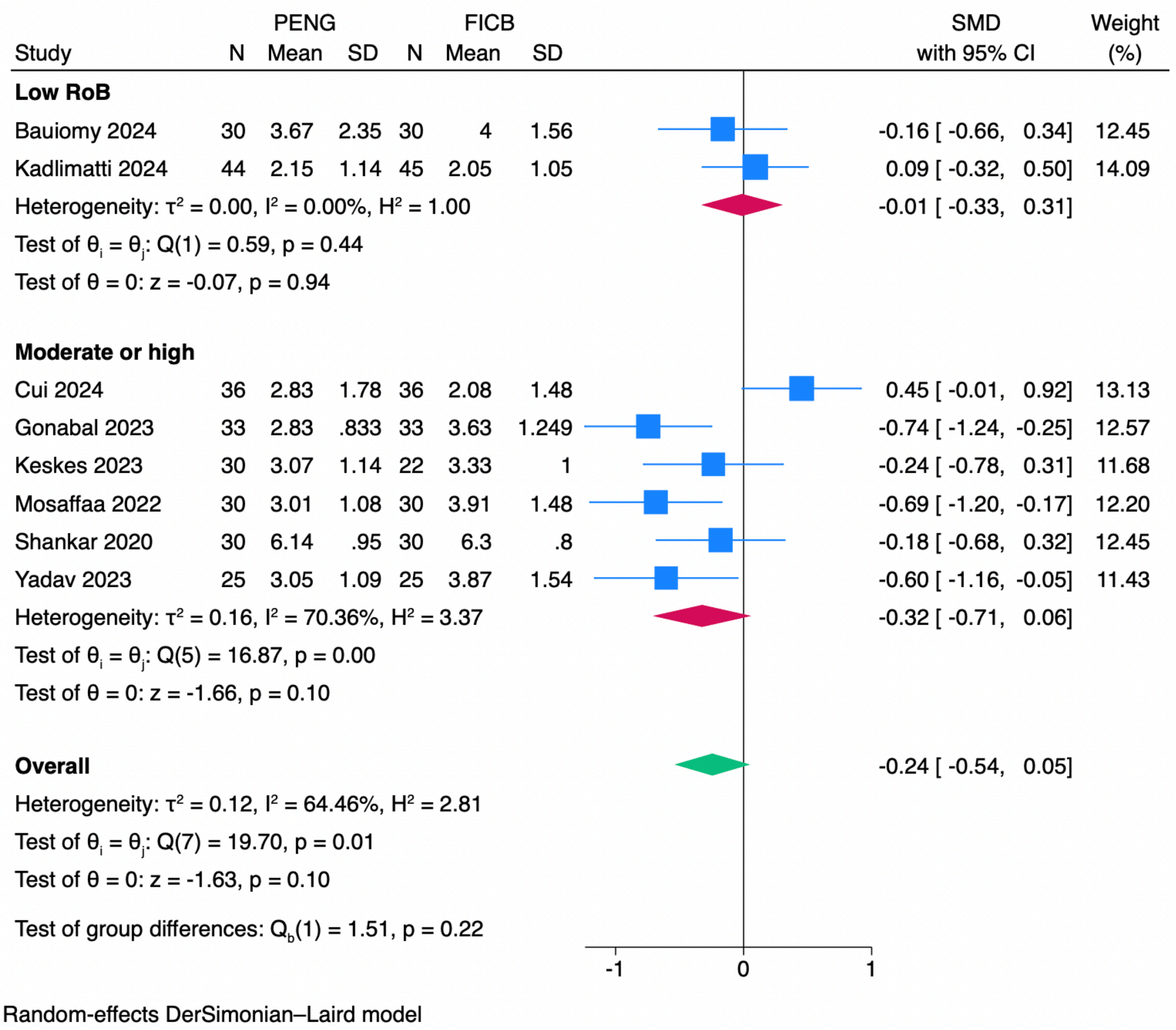

Pain at 12 hours postoperatively - supra-inguinal approach of FICB vs infra-inguinal or unknown

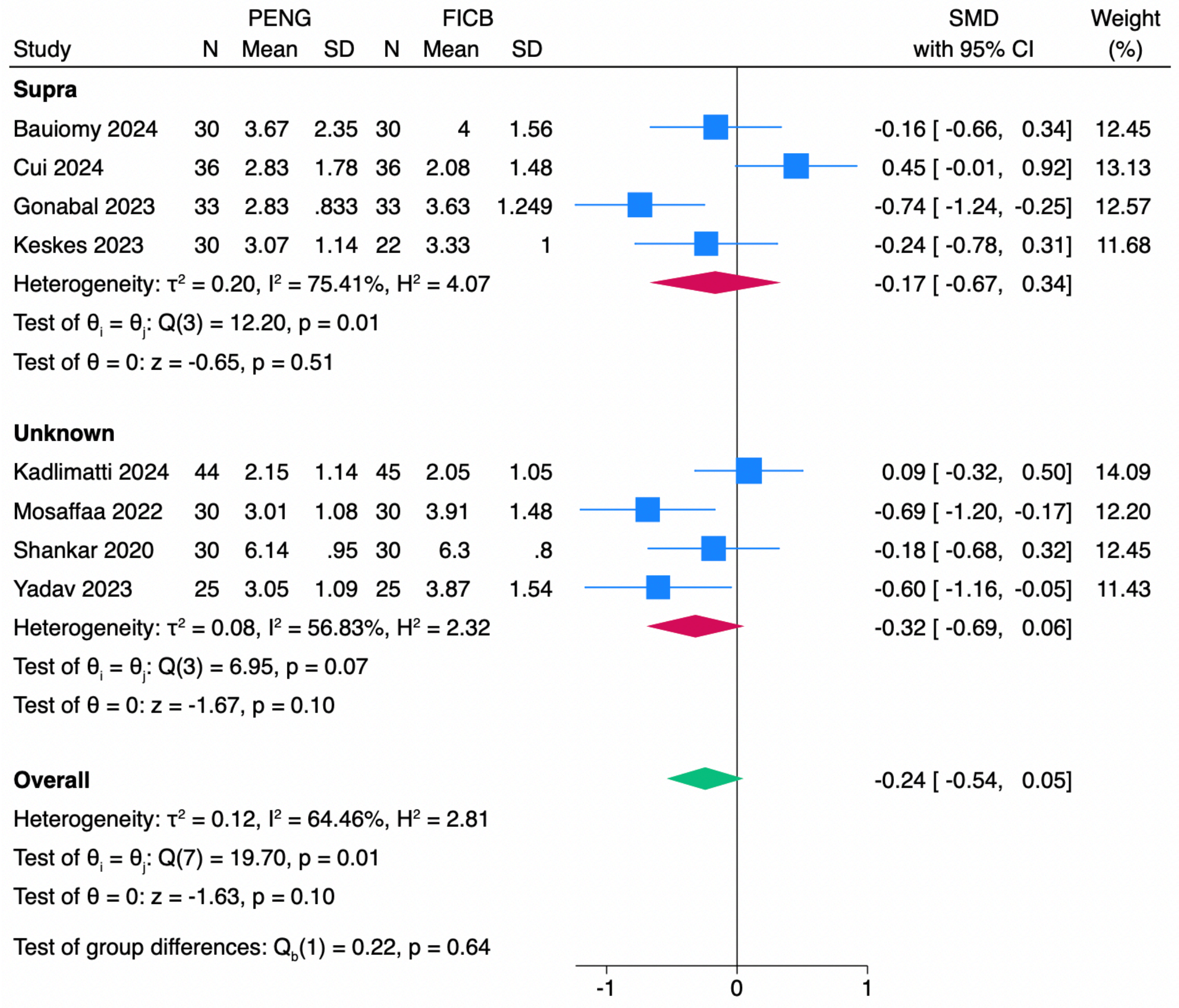

Random-effects DerSimonian–Laird model

Pain at 12 hours postoperatively - different volumes/doses of anaesthetic used in each group

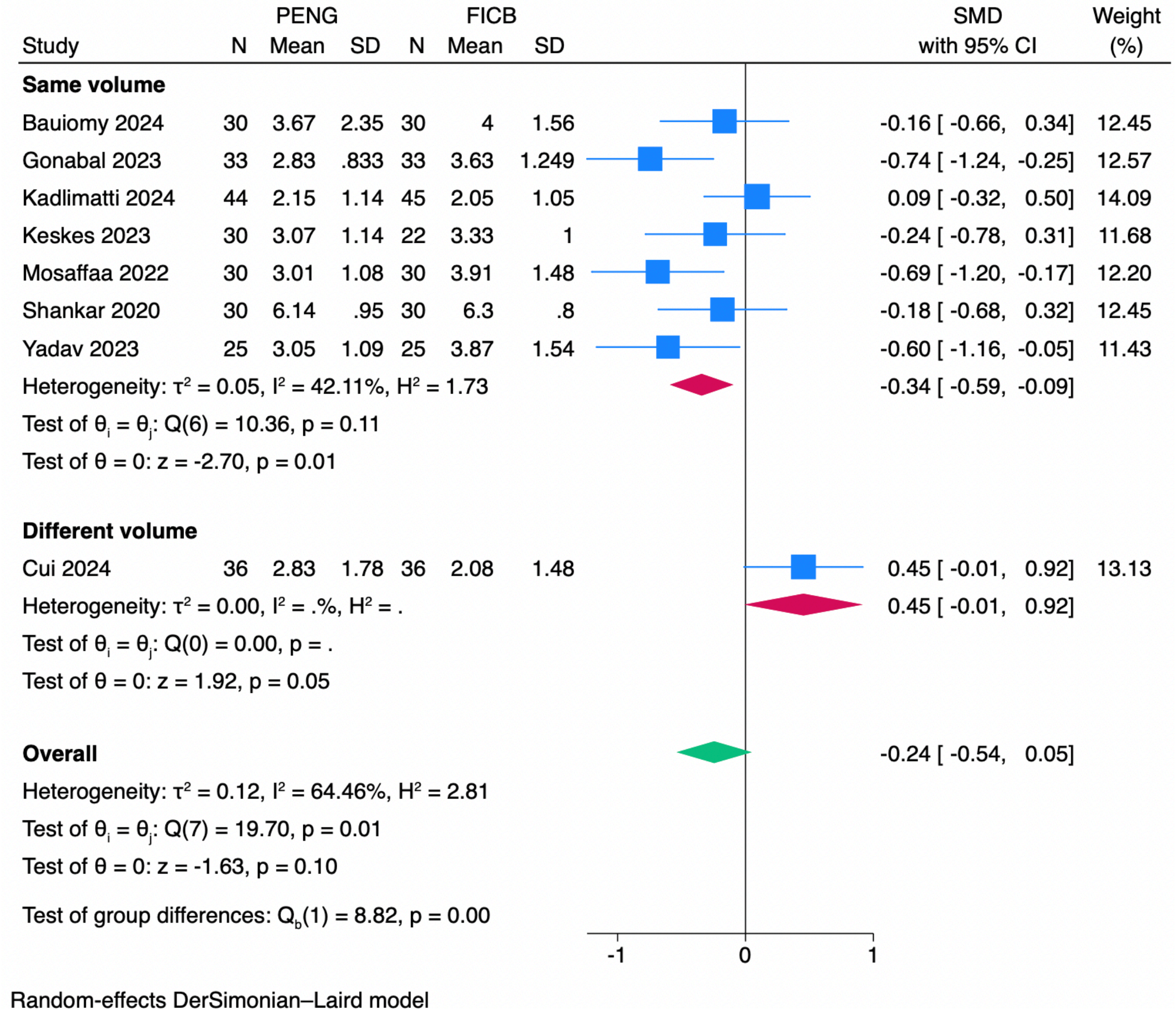

Pain at 12 hours postoperatively - spinal or general anaesthetic

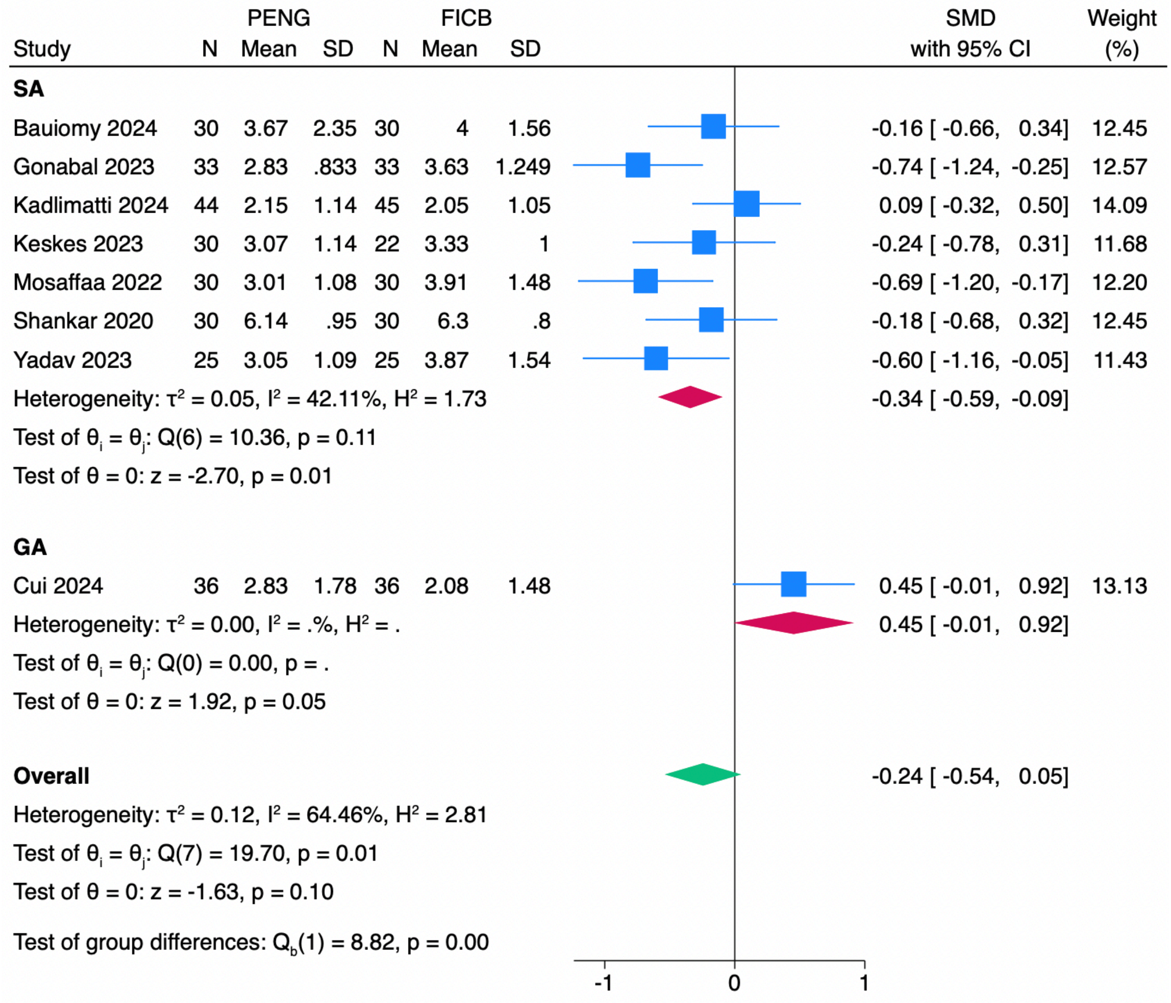

Random-effects DerSimonian–Laird model

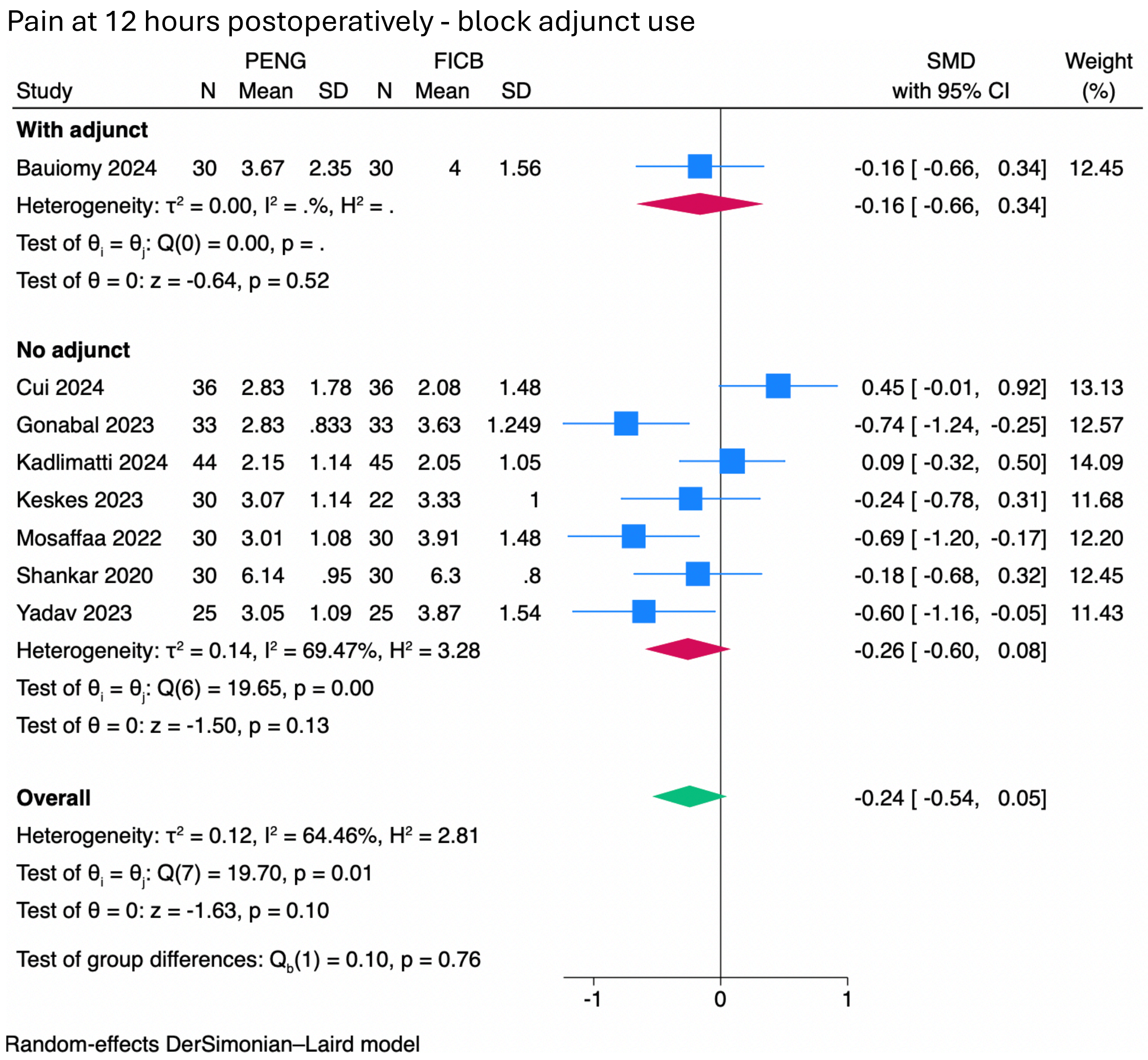
Pain at 12 hours postoperatively - block adjunct use
PENG
FICB
Study N Mean SD N Mean SD
SMD with 95% CI
Weight (%)
With adjunct
Bauiomy 2024 30 3.67 2.35 30 4 1.56 -0.16 [-0.66, 0.34] 12.45
Heterogeneity: τ² = 0.00, I² = .%, H² = . -0.16 [-0.66, 0.34]
Test of θi = θj: Q(0) = 0.00, p = .
Test of θ = 0: z = -0.64, p = 0.52
No adjunct
Cui 2024 36 2.83 1.78 36 2.08 1.48 0.45 [-0.01, 0.92] 13.13
Gonabal 2023 33 2.83 .833 33 3.63 1.249 -0.74 [-1.24, -0.25] 12.57
Kadlimatti 2024 44 2.15 1.14 45 2.05 1.05 0.09 [-0.32, 0.50] 14.09
Keskes 2023 30 3.07 1.14 22 3.33 1 -0.24 [-0.78, 0.31] 11.68
Mosaffaa 2022 30 3.01 1.08 30 3.91 1.48 -0.69 [-1.20, -0.17] 12.20
Shankar 2020 30 6.14 .95 30 6.3 .8 -0.18 [-0.68, 0.32] 12.45
Yadav 2023 25 3.05 1.09 25 3.87 1.54 -0.60 [-1.16, -0.05] 11.43
Heterogeneity: τ² = 0.14, I² = 69.47%, H² = 3.28 -0.26 [-0.60, 0.08]
Test of θi = θj: Q(6) = 19.65, p = 0.00
Test of θ = 0: z = -1.50, p = 0.13
Overall -0.24 [-0.54, 0.05]
Heterogeneity: τ² = 0.12, I² = 64.46%, H² = 2.81
Test of θi = θj: Q(7) = 19.70, p = 0.01
Test of θ = 0: z = -1.63, p = 0.10
Test of group differences: Qb(1) = 0.10, p = 0.76
-1
0
1
Random-effects DerSimonian–Laird model

Pain at 12 hours postoperatively - leave-one-out analysis

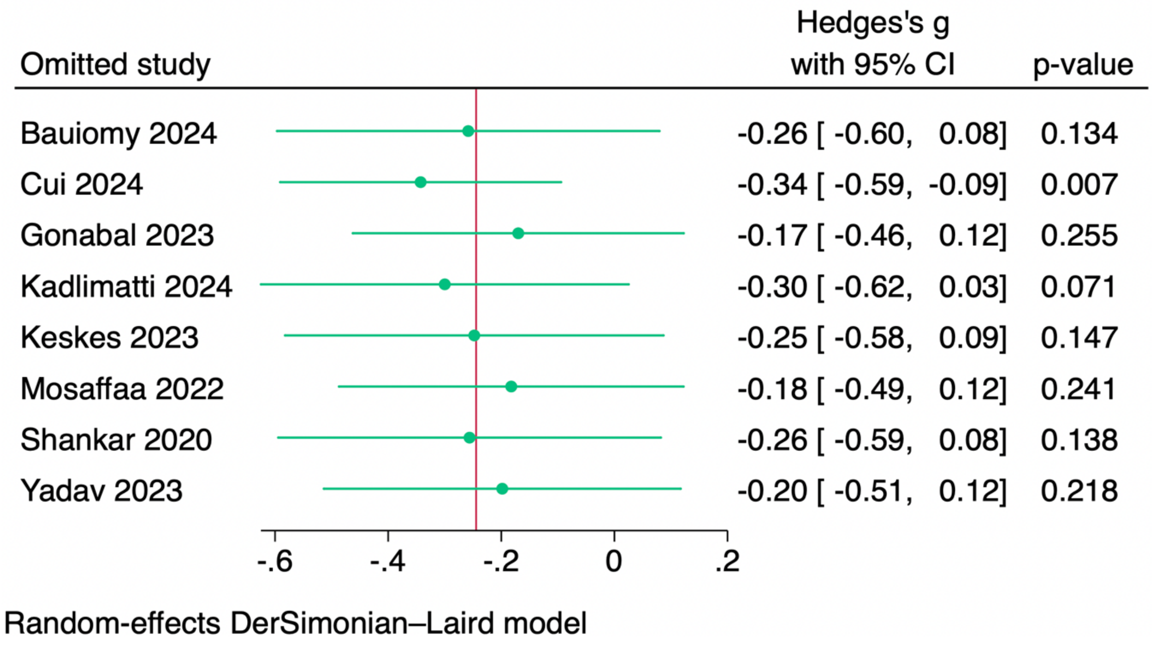

Pain scores at 24 hours postoperatively – subgroup analyses

Pain at 24 hours postoperatively - timing of administration of the block (pre- or post-anaesthesia)

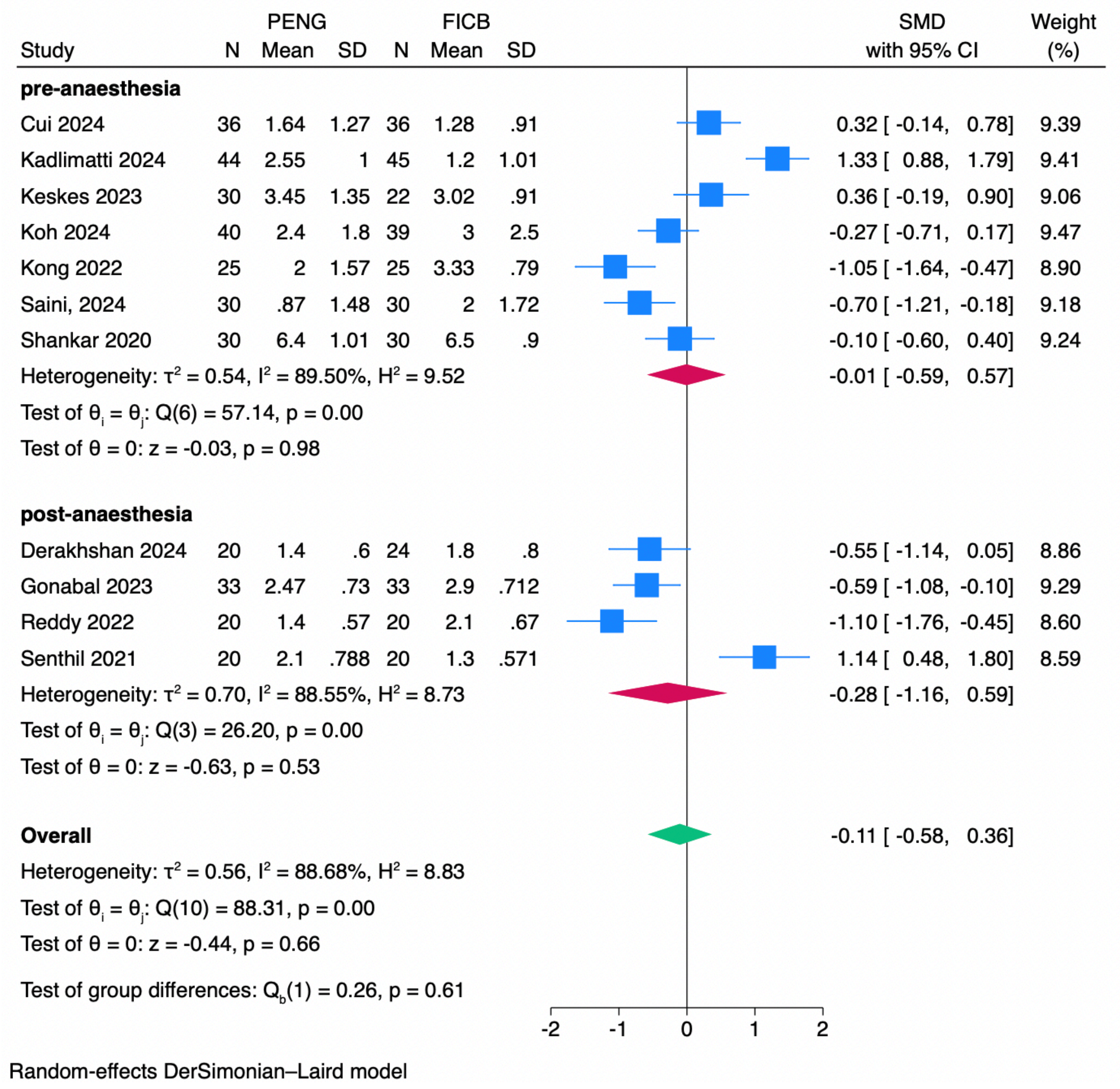

Pain at 24 hours postoperatively - low risk of bias

Study	PENG N	PENG Mean	PENG SD	FICB N	FICB Mean	FICB SD	SMD with 95% CI	Weight (%)
**Low RoB**								
Kadlimatti 2024	44	2.55	1	45	1.2	1.01	1.33 [0.88, 1.79]	9.41
Koh 2024	40	2.4	1.8	39	3	2.5	-0.27 [-0.71, 0.17]	9.47
Kong 2022	25	2	1.57	25	3.33	.79	-1.05 [-1.64, -0.47]	8.90
Saini, 2024	30	.87	1.48	30	2	1.72	-0.70 [-1.21, -0.18]	9.18
Senthil 2021	20	2.1	.788	20	1.3	.571	1.14 [0.48, 1.80]	8.59
Heterogeneity: $\tau^2 = 1.06$, $I^2 = 93.81\%$, $H^2 = 16.15$							0.09 [-0.84, 1.02]	
Test of $\theta_i = \theta_j$: Q(4) = 64.60, p = 0.00								
Test of $\theta = 0$: z = 0.19, p = 0.85								
**Moderate or high**								
Cui 2024	36	1.64	1.27	36	1.28	.91	0.32 [-0.14, 0.78]	9.39
Derakhshan 2024	20	1.4	.6	24	1.8	.8	-0.55 [-1.14, 0.05]	8.86
Gonabal 2023	33	2.47	.73	33	2.9	.712	-0.59 [-1.08, -0.10]	9.29
Keskes 2023	30	3.45	1.35	22	3.02	.91	0.36 [-0.19, 0.90]	9.06
Reddy 2022	20	1.4	.57	20	2.1	.67	-1.10 [-1.76, -0.45]	8.60
Shankar 2020	30	6.4	1.01	30	6.5	.9	-0.10 [-0.60, 0.40]	9.24
Heterogeneity: $\tau^2 = 0.22$, $I^2 = 75.24\%$, $H^2 = 4.04$							-0.26 [-0.69, 0.18]	
Test of $\theta_i = \theta_j$: Q(5) = 20.20, p = 0.00								
Test of $\theta = 0$: z = -1.14, p = 0.25								
**Overall**							-0.11 [-0.58, 0.36]	
Heterogeneity: $\tau^2 = 0.56$, $I^2 = 88.68\%$, $H^2 = 8.83$								
Test of $\theta_i = \theta_j$: Q(10) = 88.31, p = 0.00								
Test of $\theta = 0$: z = -0.44, p = 0.66								
Test of group differences: $Q_b(1) = 0.43$, p = 0.51								

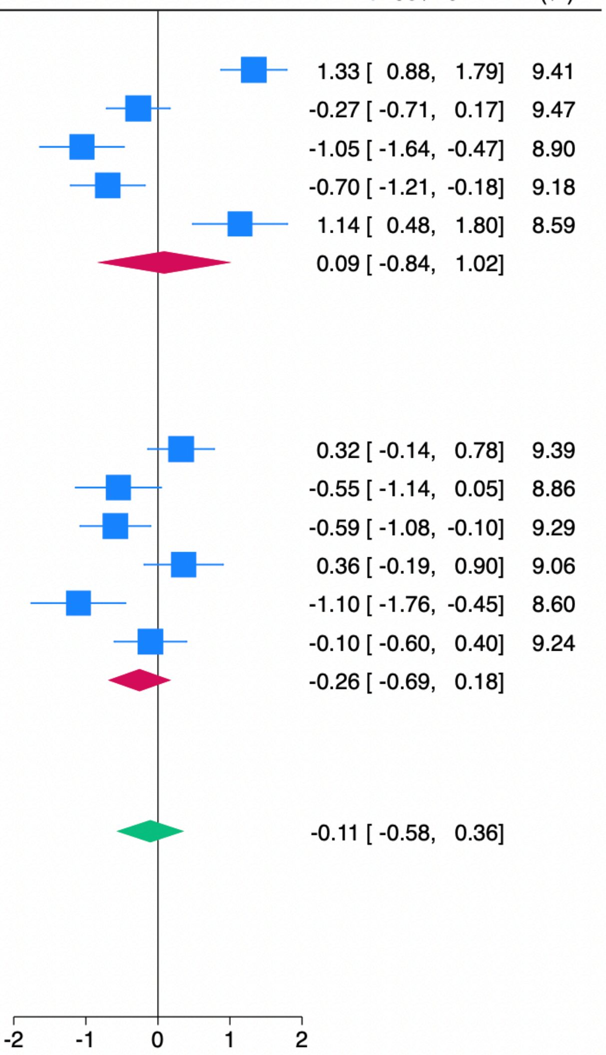

Random-effects DerSimonian–Laird model

Pain at 24 hours postoperatively - supra-inguinal approach of FICB vs infra-inguinal or unknown

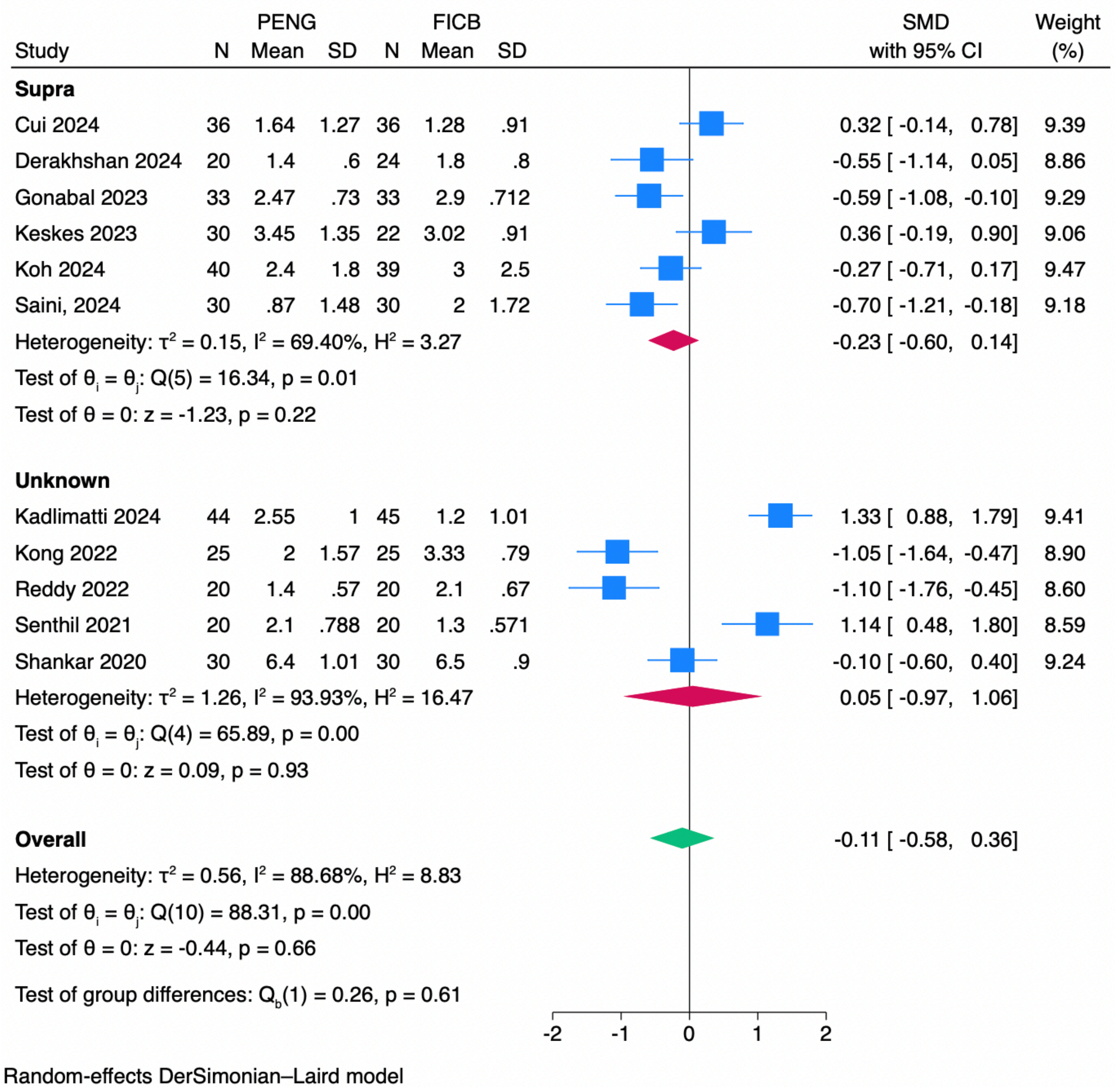

Pain at 24 hours postoperatively - different volumes/doses of anaesthetic used in each group

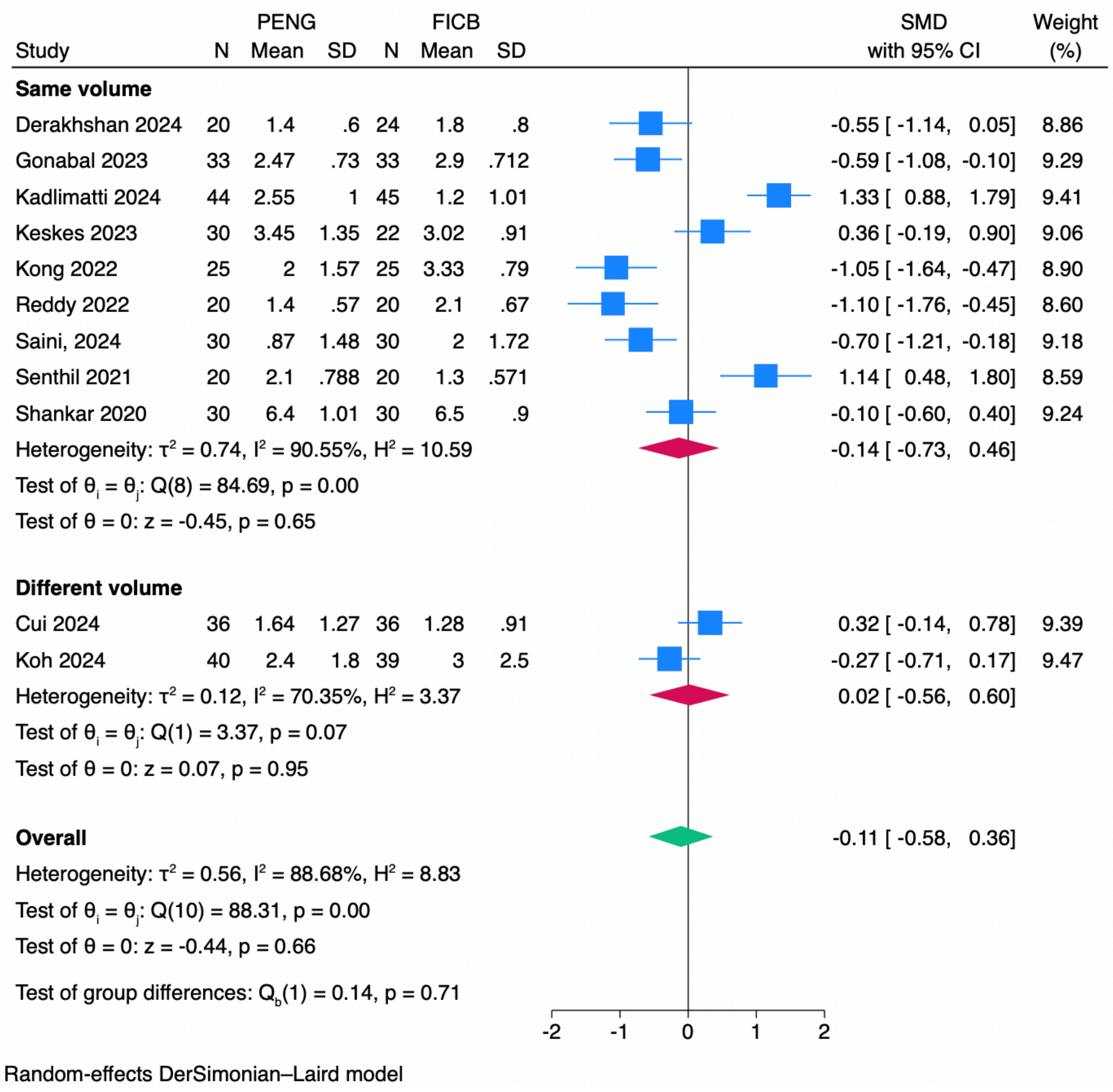

Pain at 24 hours postoperatively - spinal or general anaesthetic

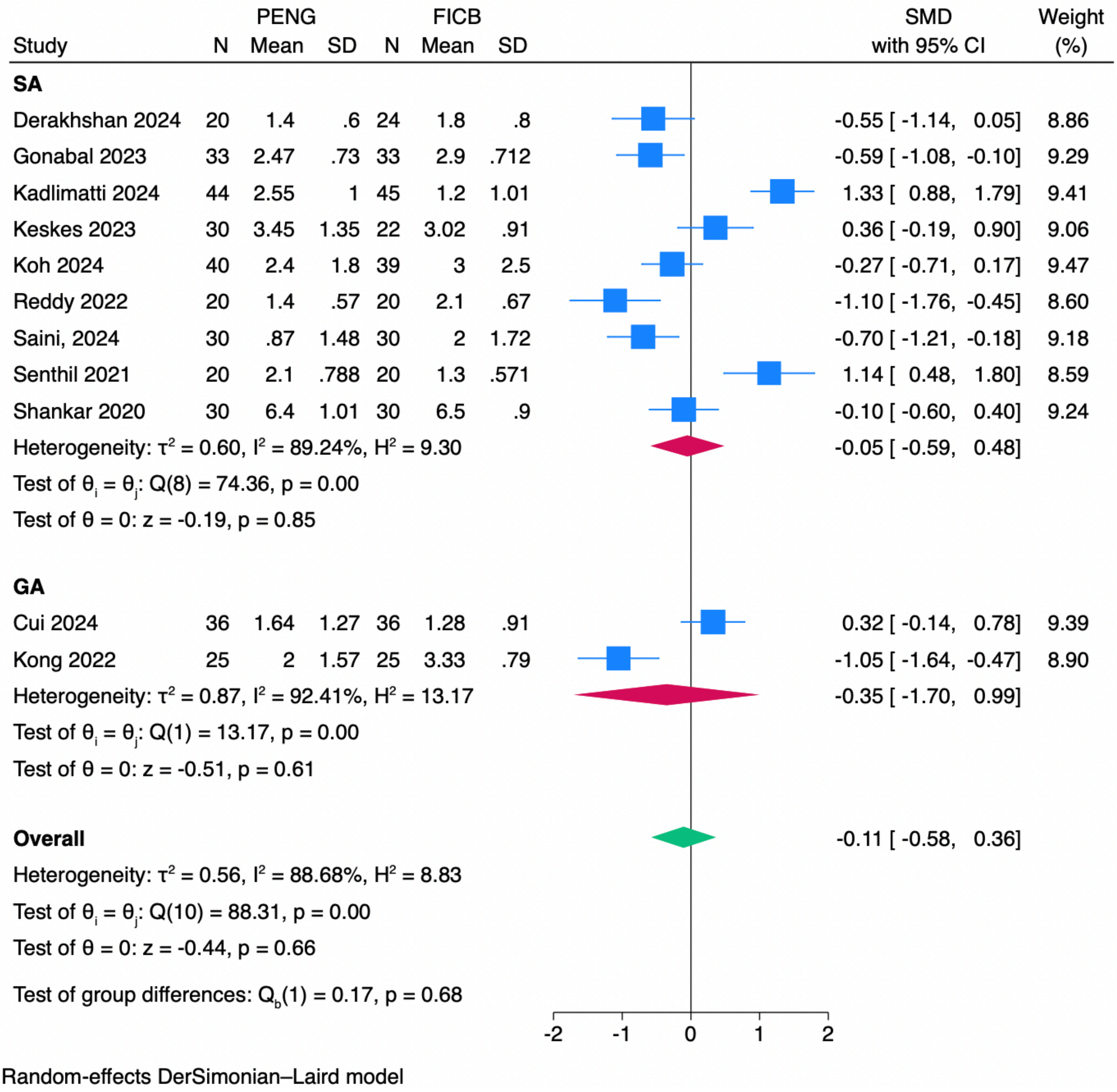

Pain at 24 hours postoperatively - block adjunct use

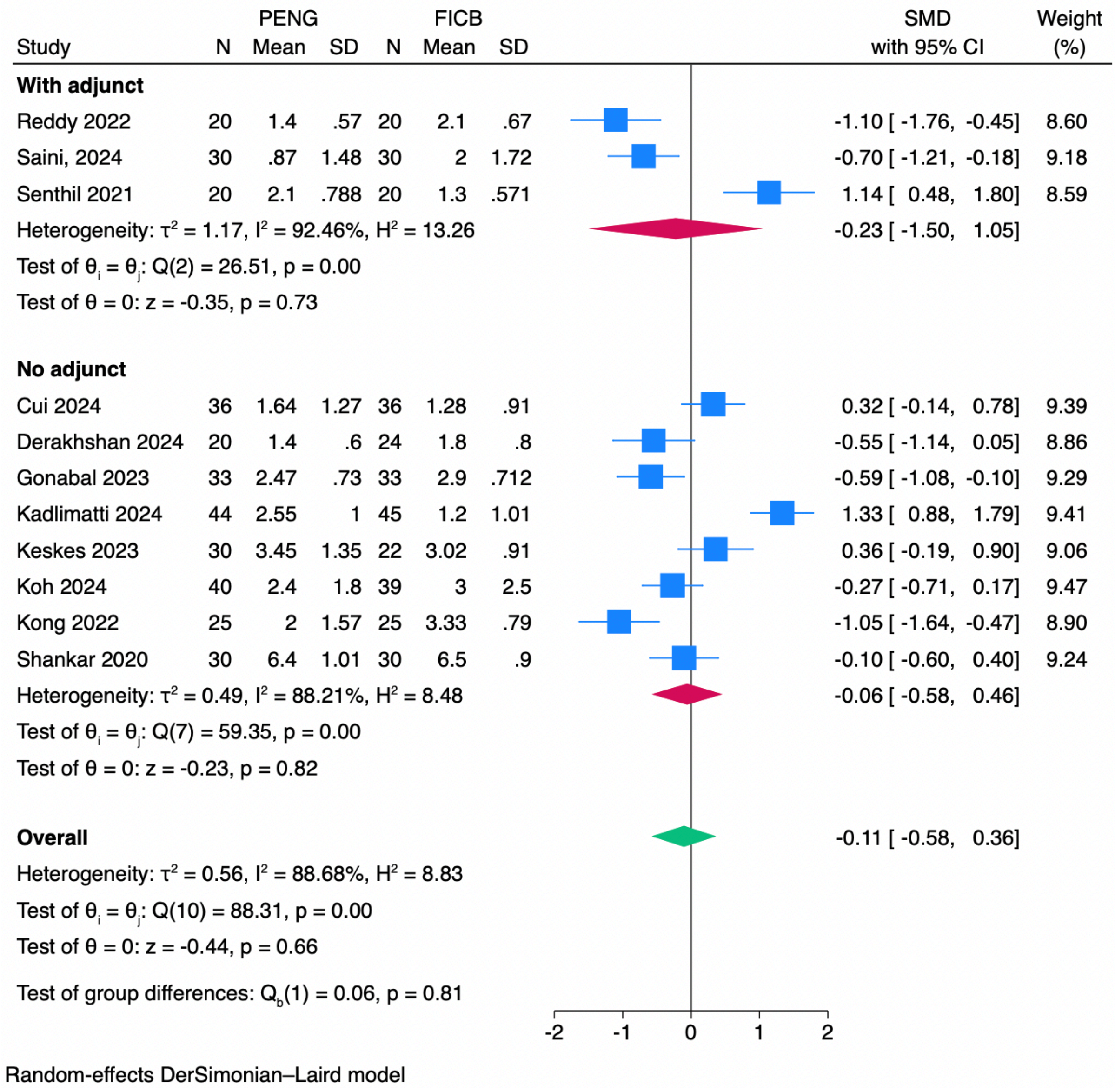

Pain at 24 hours postoperatively - leave-one-out analysis

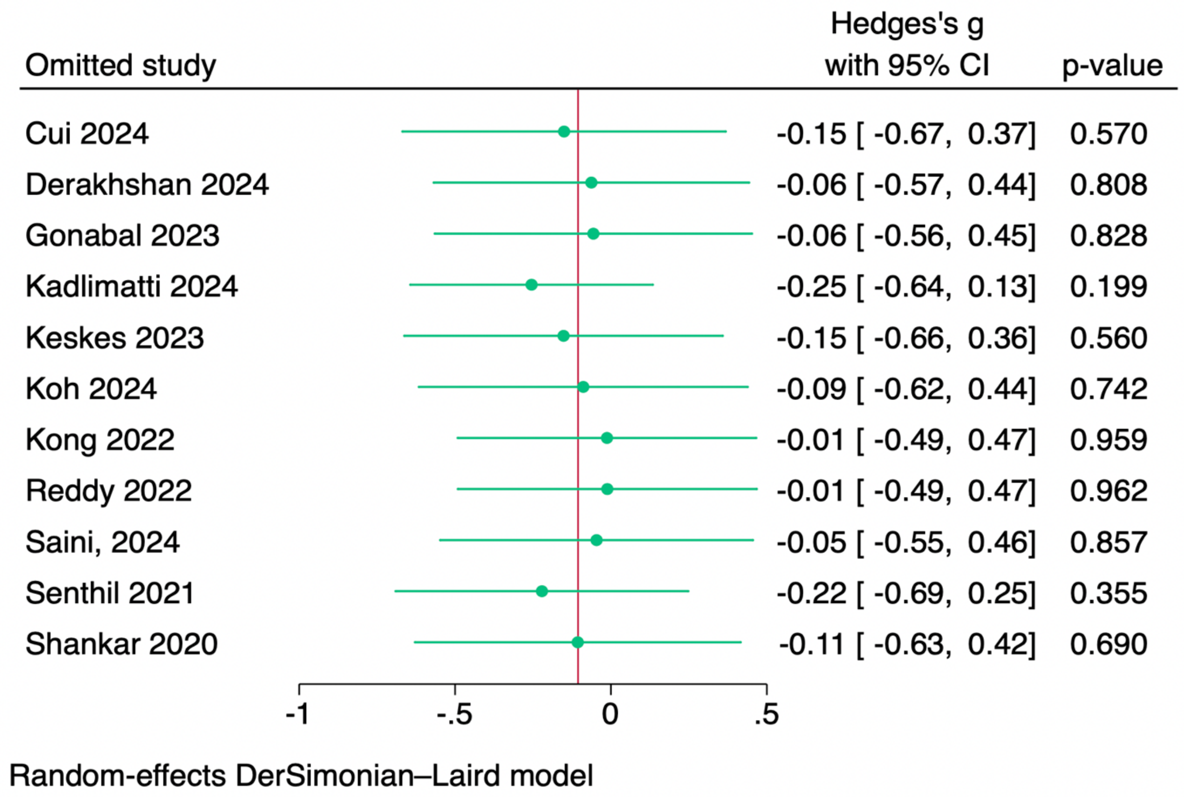

Pain at 24 hours postoperatively - outlier analysis

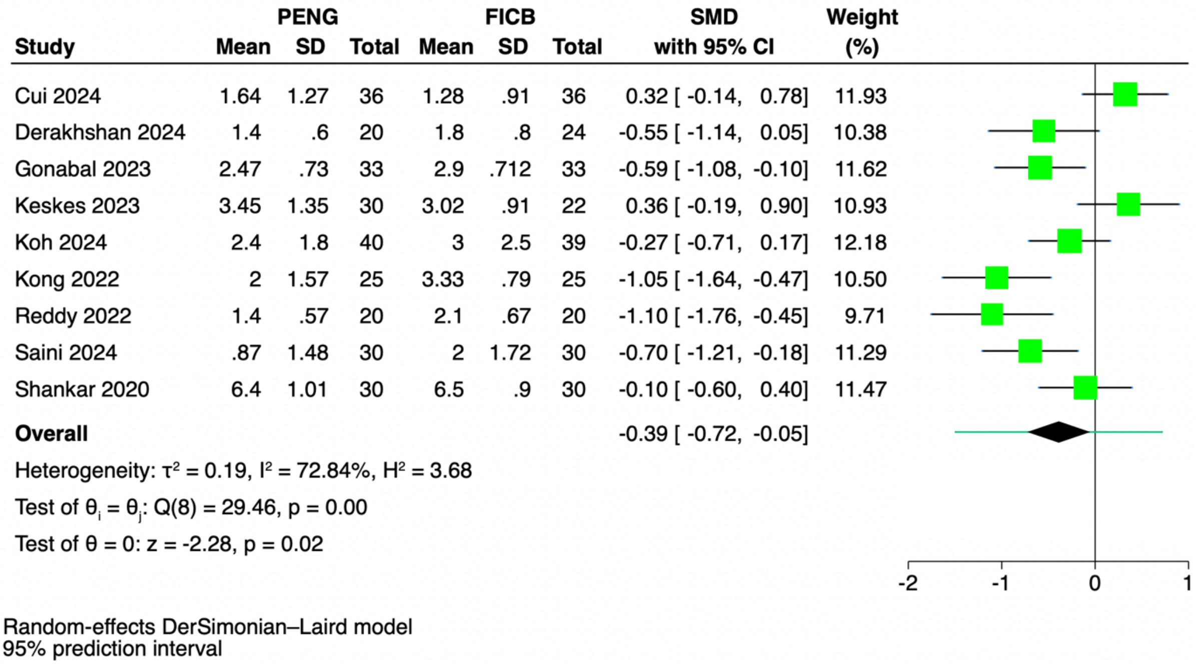

Pain at 24 hours postoperatively – funnel plot

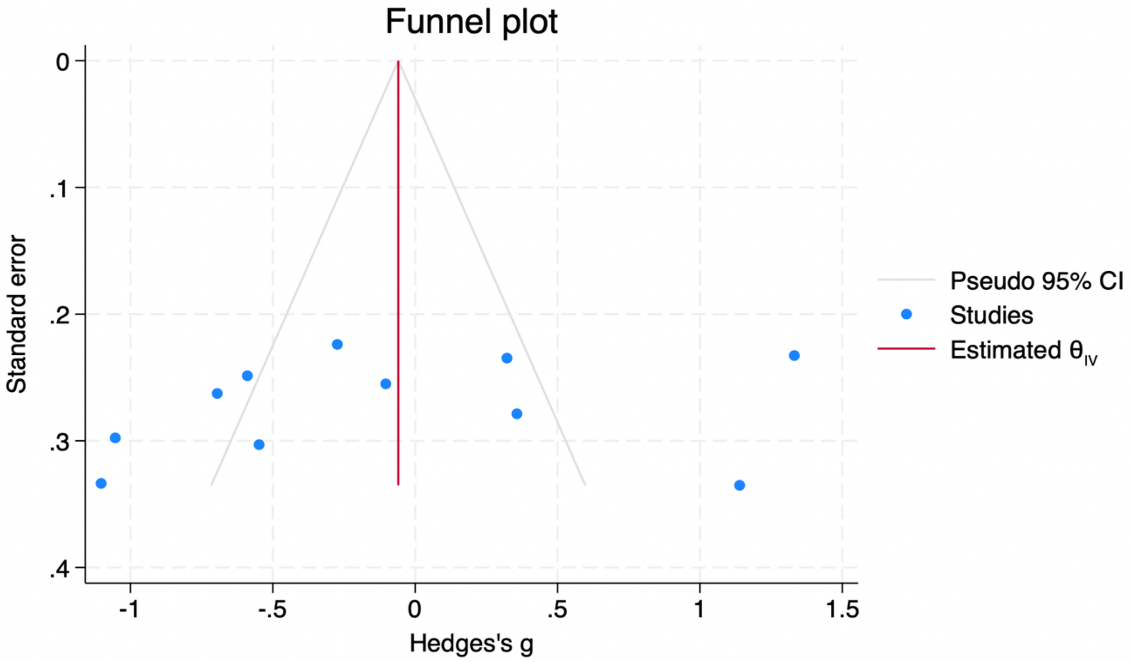

Dynamic pain scores at 6 hours postoperatively

Dynamic pain scores at 6 hours postoperatively – forest plot

Study	PENG Mean	PENG SD	PENG Total	FICB Mean	FICB SD	FICB Total	SMD with 95% CI	Weight (%)
Gonabal 2023	2.97	.928	33	2.7	1.055	33	0.27 [-0.21, 0.75]	25.80
Koh 2024	3.8	2	40	3.9	2.7	39	-0.04 [-0.48, 0.40]	26.34
Kong 2022	2.67	1.57	25	5.67	2.36	25	-1.47 [-2.09, -0.86]	23.86
Senthil 2022	1.4	.981	20	1.65	8.489	20	-0.04 [-0.65, 0.57]	24.00
**Overall**							-0.30 [-1.00, 0.39]	

Heterogeneity: $\tau^2 = 0.43$, $I^2 = 85.57\%$, $H^2 = 6.93$

Test of $\theta_i = \theta_j$: Q(3) = 20.78, p = 0.00

Test of $\theta = 0$: z = -0.86, p = 0.39

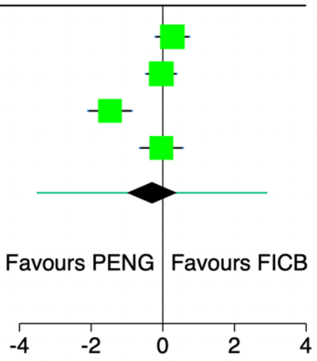

Random-effects DerSimonian–Laird model
95% prediction interval

Dynamic pain scores at 6 hours postoperatively – subgroup analyses

Dynamic pain at 6 hours postoperatively - timing of administration of the block (pre- or post-anaesthesia)

Study	PENG N	PENG Mean	PENG SD	FICB N	FICB Mean	FICB SD	SMD with 95% CI	Weight (%)
**pre-anaesthesia**								
Koh 2024	40	4.7	2.4	39	5.1	2.7	-0.16 [-0.59, 0.28]	26.99
Kong 2022	25	3.17	1.97	25	5.33	2.36	-0.98 [-1.56, -0.40]	23.95
Heterogeneity: $\tau^2 = 0.27$, $I^2 = 79.79\%$, $H^2 = 4.95$							-0.54 [-1.35, 0.26]	
Test of $\theta_i = \theta_j$: Q(1) = 4.95, p = 0.03								
Test of $\theta = 0$: z = -1.32, p = 0.19								
**post-anaesthesia**								
Gonabal 2023	33	2.55	.682	33	2.97	.974	-0.49 [-0.98, -0.01]	25.99
Senthil 2022	20	1.65	.812	20	1.25	.639	0.54 [-0.08, 1.16]	23.07
Heterogeneity: $\tau^2 = 0.45$, $I^2 = 84.86\%$, $H^2 = 6.60$							0.00 [-1.01, 1.01]	
Test of $\theta_i = \theta_j$: Q(1) = 6.60, p = 0.01								
Test of $\theta = 0$: z = 0.01, p = 1.00								
**Overall**							-0.28 [-0.83, 0.27]	
Heterogeneity: $\tau^2 = 0.24$, $I^2 = 77.50\%$, $H^2 = 4.45$								
Test of $\theta_i = \theta_j$: Q(3) = 13.34, p = 0.00								
Test of $\theta = 0$: z = -1.00, p = 0.32								
Test of group differences: $Q_b(1) = 0.69$, p = 0.41								

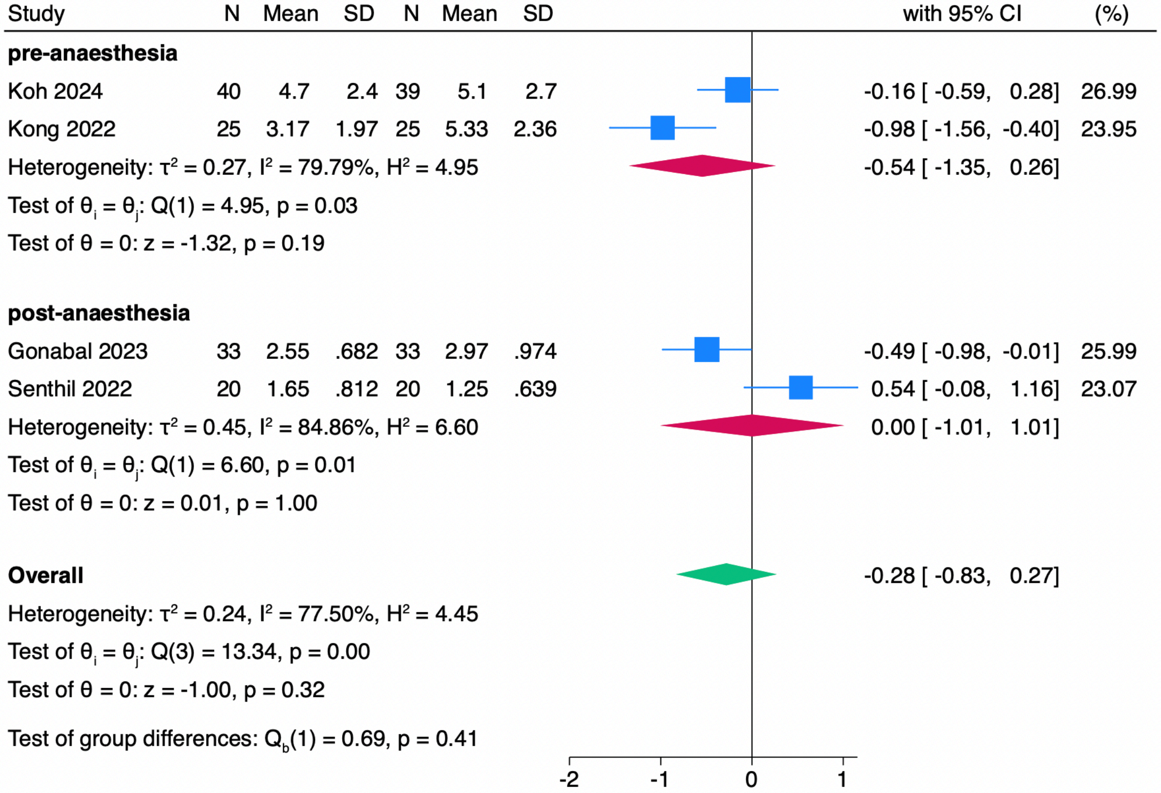

Random-effects DerSimonian–Laird model

Dynamic pain at 6 hours postoperatively - low risk of bias

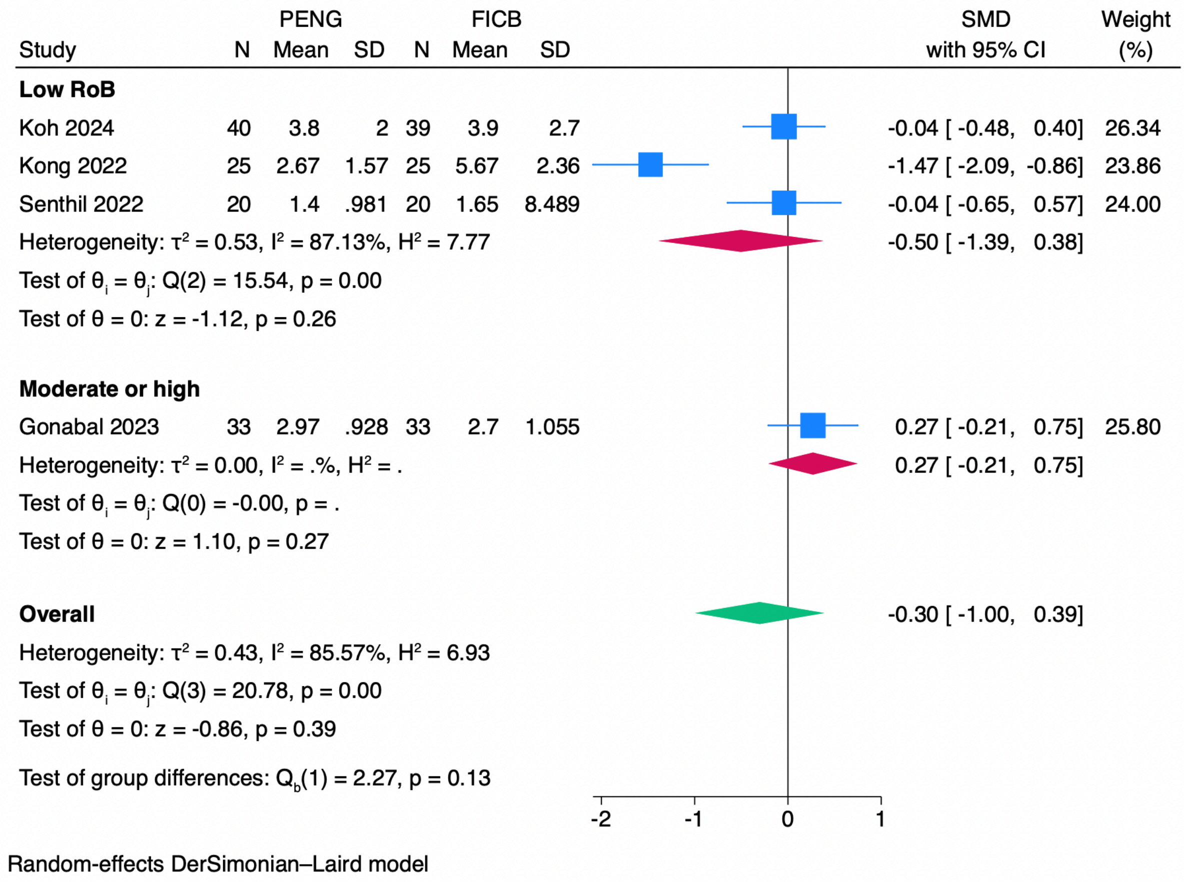

Dynamic pain at 6 hours postoperatively - supra-inguinal approach of FICB vs infra-inguinal or unknown

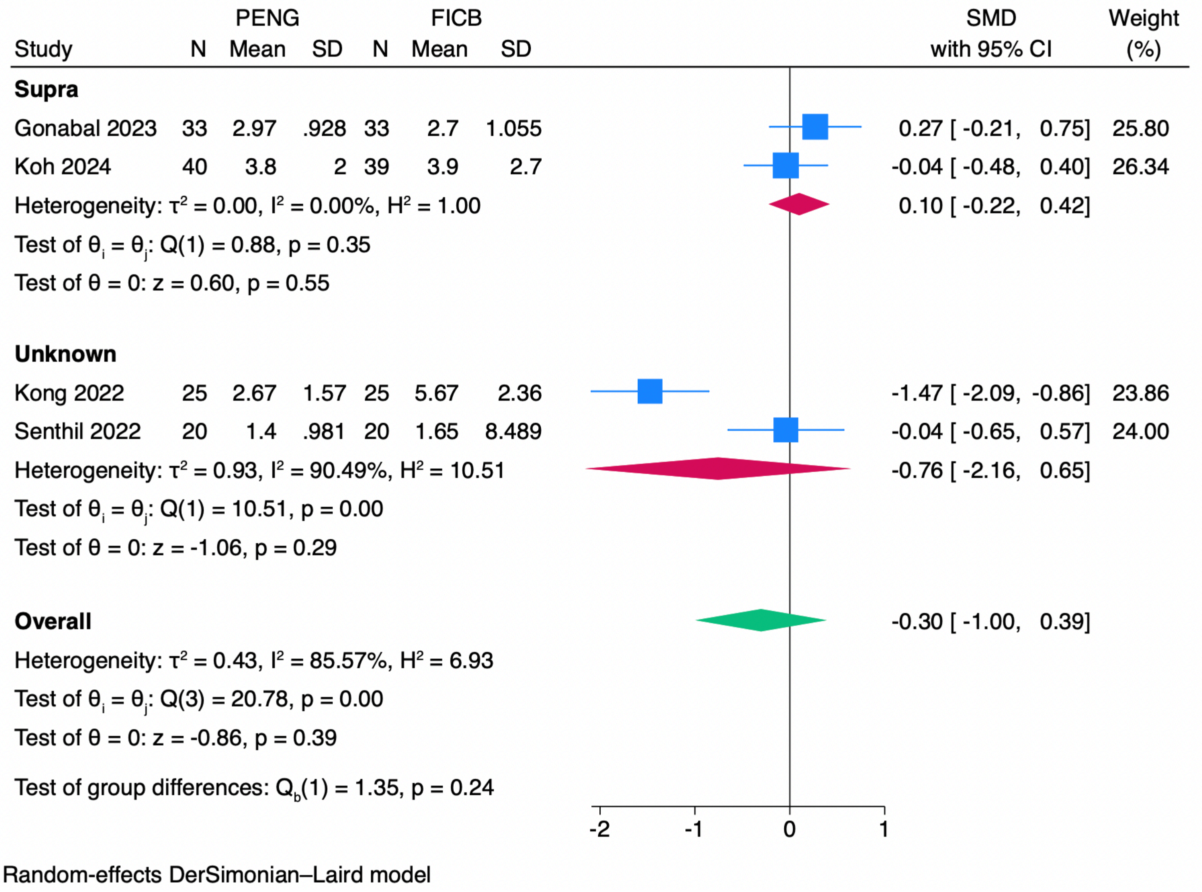

Dynamic pain at 6 hours postoperatively - different volumes/doses of anaesthetic used in each group

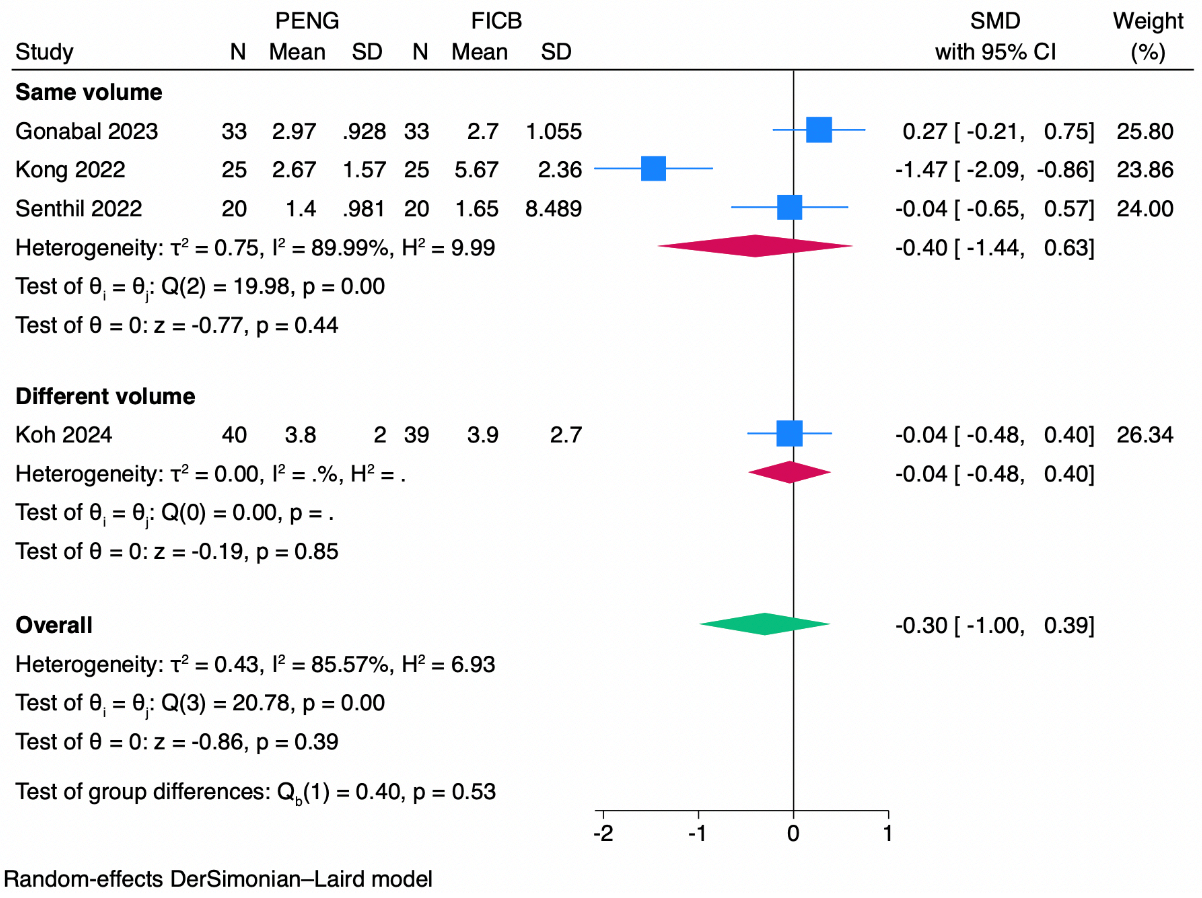

Dynamic pain at 6 hours postoperatively - spinal or general anaesthetic

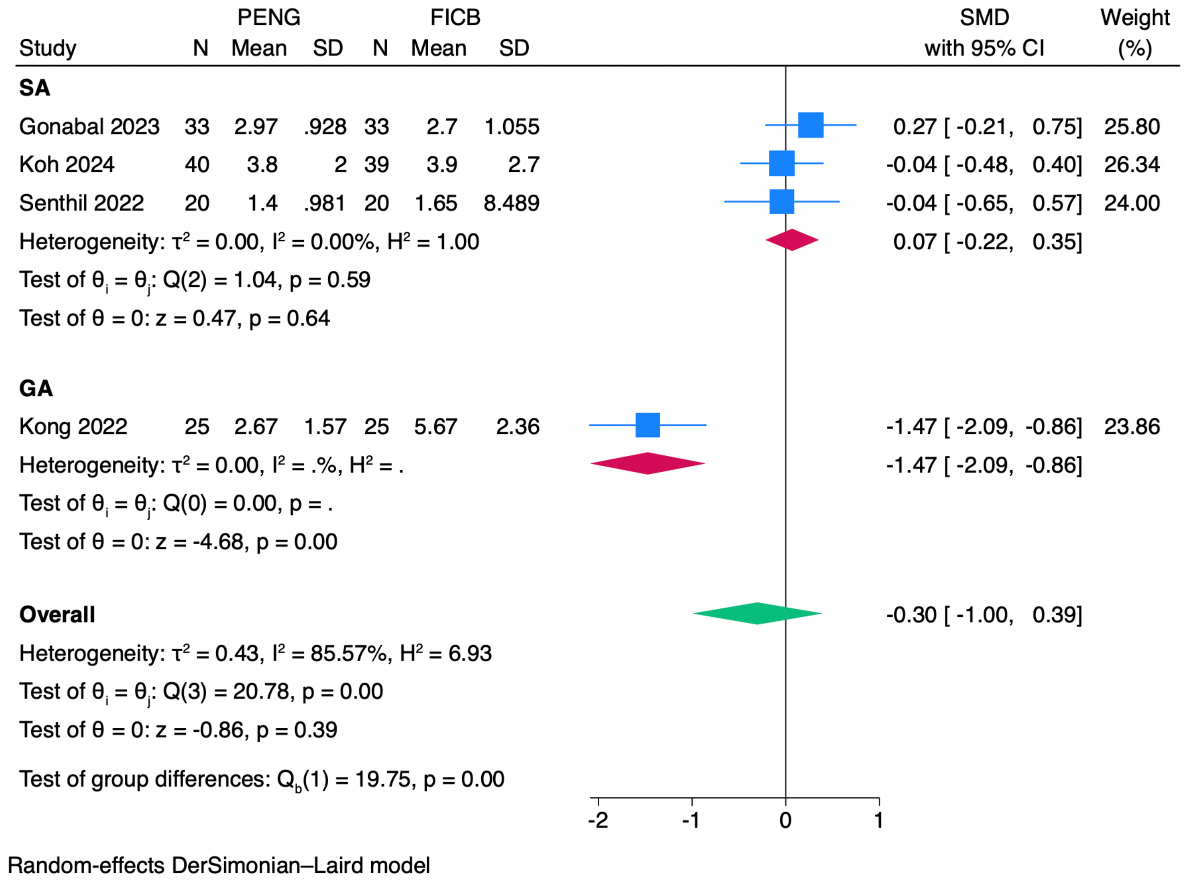

Dynamic pain at 6 hours postoperatively - block adjunct use

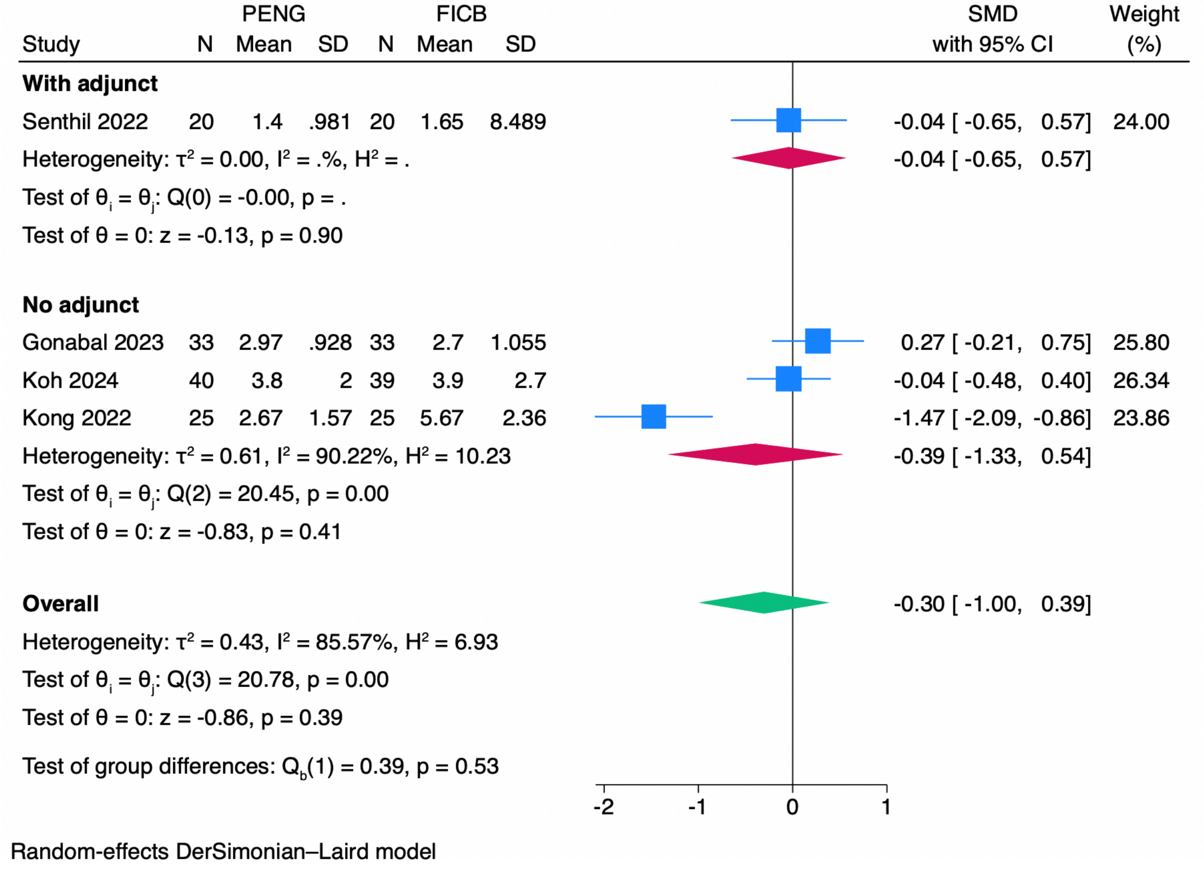

Dynamic pain at 6 hours postoperatively - leave-one-out analysis

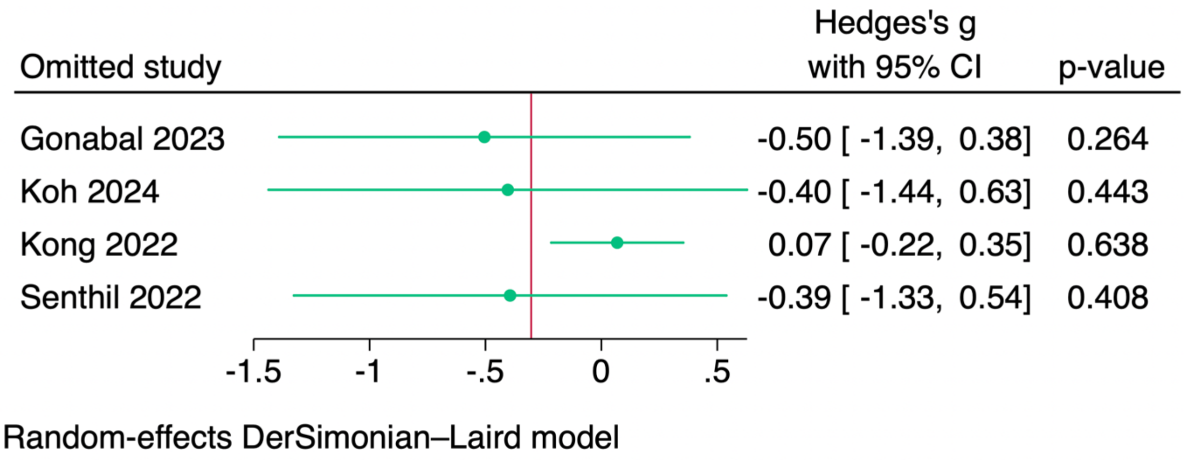

Dynamic pain scores at 24 hours postoperatively

Dynamic pain scores at 24 hours postoperatively – forest plot

Study	PENG Mean	PENG SD	PENG Total	FICB Mean	FICB SD	FICB Total	SMD with 95% CI	Weight (%)
Gonabal 2023	2.55	.682	33	2.97	.974	33	-0.49 [-0.98, -0.01]	25.99
Koh 2024	4.7	2.4	40	5.1	2.7	39	-0.16 [-0.59, 0.28]	26.99
Kong 2022	3.17	1.97	25	5.33	2.36	25	-0.98 [-1.56, -0.40]	23.95
Senthil 2022	1.65	.812	20	1.25	.639	20	0.54 [-0.08, 1.16]	23.07
**Overall**							-0.28 [-0.83, 0.27]	

Heterogeneity: $\tau^2 = 0.24$, $I^2 = 77.50\%$, $H^2 = 4.45$

Test of $\theta_i = \theta_j$: $Q(3) = 13.34$, $p = 0.00$

Test of $\theta = 0$: $z = -1.00$, $p = 0.32$

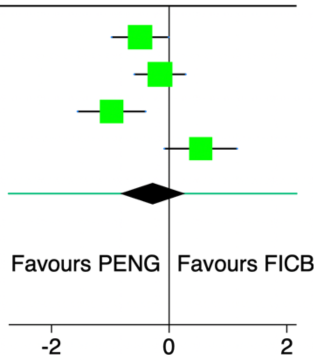

Random-effects DerSimonian–Laird model
95% prediction interval

Dynamic pain scores at 24 hours postoperatively – subgroup analyses

Dynamic pain at 24 hours postoperatively - timing of administration of the block (pre- or post-anaesthesia)

Study	PENG N	PENG Mean	PENG SD	FICB N	FICB Mean	FICB SD	SMD with 95% CI	Weight (%)
**pre-anaesthesia**								
Koh 2024	40	4.7	2.4	39	5.1	2.7	-0.16 [-0.59, 0.28]	26.99
Kong 2022	25	3.17	1.97	25	5.33	2.36	-0.98 [-1.56, -0.40]	23.95
Heterogeneity: $\tau^2 = 0.27$, $I^2 = 79.79\%$, $H^2 = 4.95$							-0.54 [-1.35, 0.26]	
Test of $\theta_i = \theta_j$: $Q(1) = 4.95$, $p = 0.03$								
Test of $\theta = 0$: $z = -1.32$, $p = 0.19$								
**post-anaesthesia**								
Gonabal 2023	33	2.55	.682	33	2.97	.974	-0.49 [-0.98, -0.01]	25.99
Senthil 2022	20	1.65	.812	20	1.25	.639	0.54 [-0.08, 1.16]	23.07
Heterogeneity: $\tau^2 = 0.45$, $I^2 = 84.86\%$, $H^2 = 6.60$							0.00 [-1.01, 1.01]	
Test of $\theta_i = \theta_j$: $Q(1) = 6.60$, $p = 0.01$								
Test of $\theta = 0$: $z = 0.01$, $p = 1.00$								
**Overall**							-0.28 [-0.83, 0.27]	
Heterogeneity: $\tau^2 = 0.24$, $I^2 = 77.50\%$, $H^2 = 4.45$								
Test of $\theta_i = \theta_j$: $Q(3) = 13.34$, $p = 0.00$								
Test of $\theta = 0$: $z = -1.00$, $p = 0.32$								
Test of group differences: $Q_b(1) = 0.69$, $p = 0.41$								

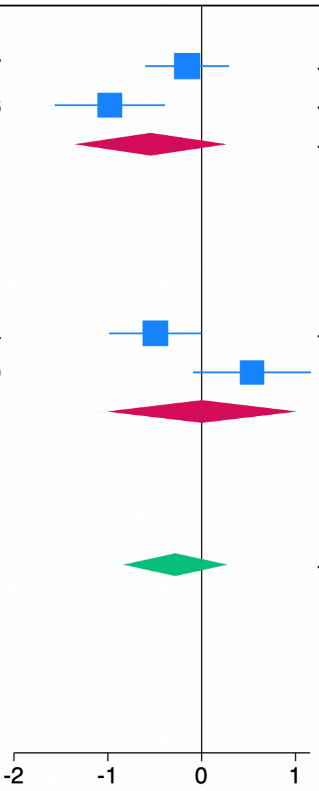

Random-effects DerSimonian–Laird model

Dynamic pain at 24 hours postoperatively - low risk of bias

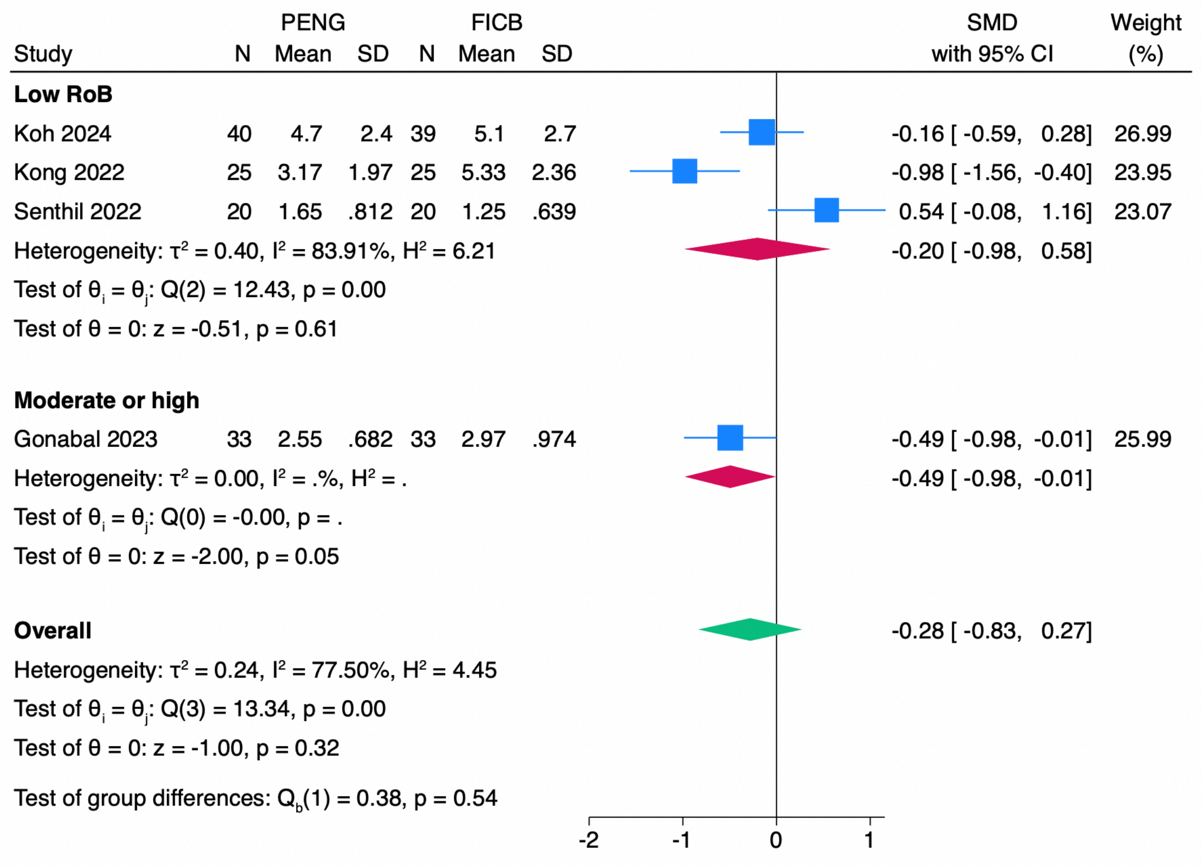

Random-effects DerSimonian–Laird model

Dynamic pain at 24 hours postoperatively - supra-inguinal approach of FICB vs infra-inguinal or unknown

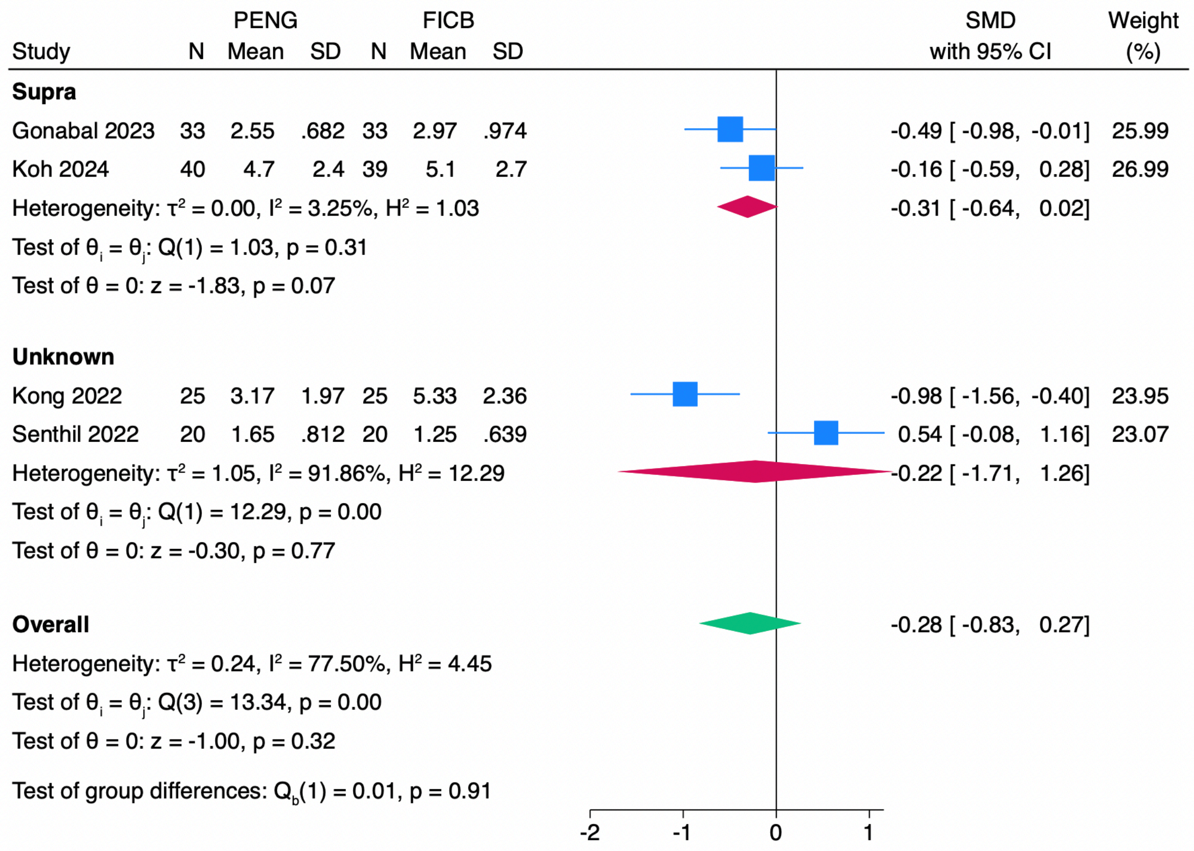

Random-effects DerSimonian–Laird model

Dynamic pain at 24 hours postoperatively - different volumes/doses of anaesthetic used in each group

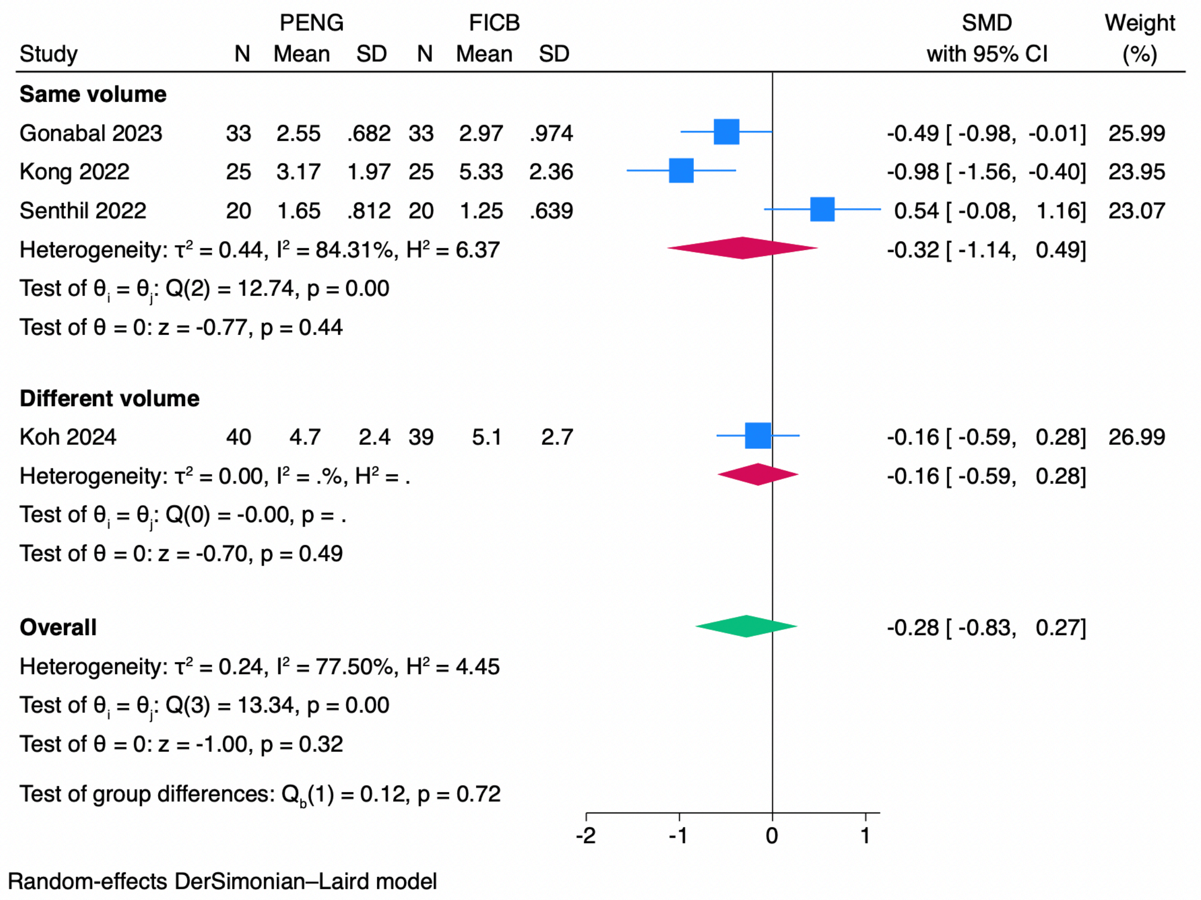

Dynamic pain at 24 hours postoperatively - spinal or general anaesthetic

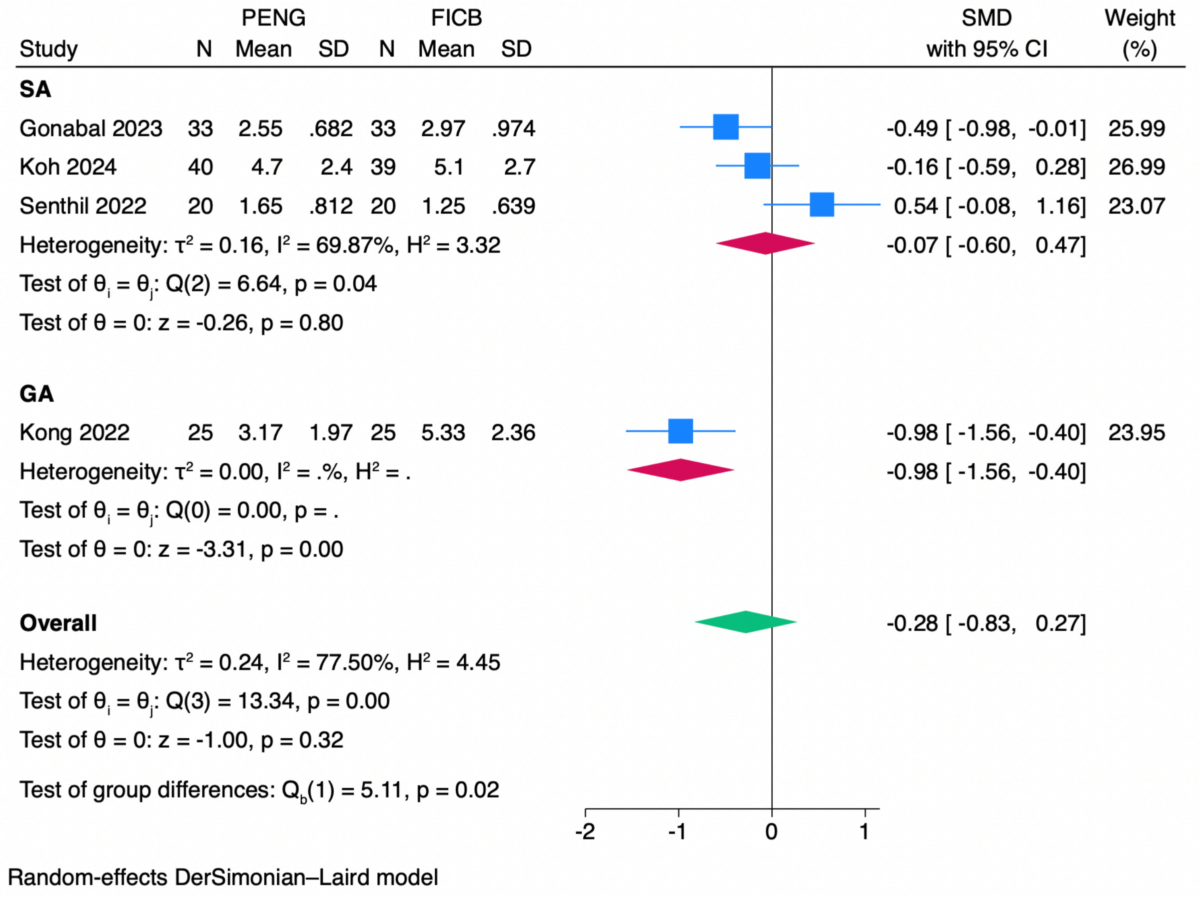

Dynamic pain at 24 hours postoperatively - block adjunct use

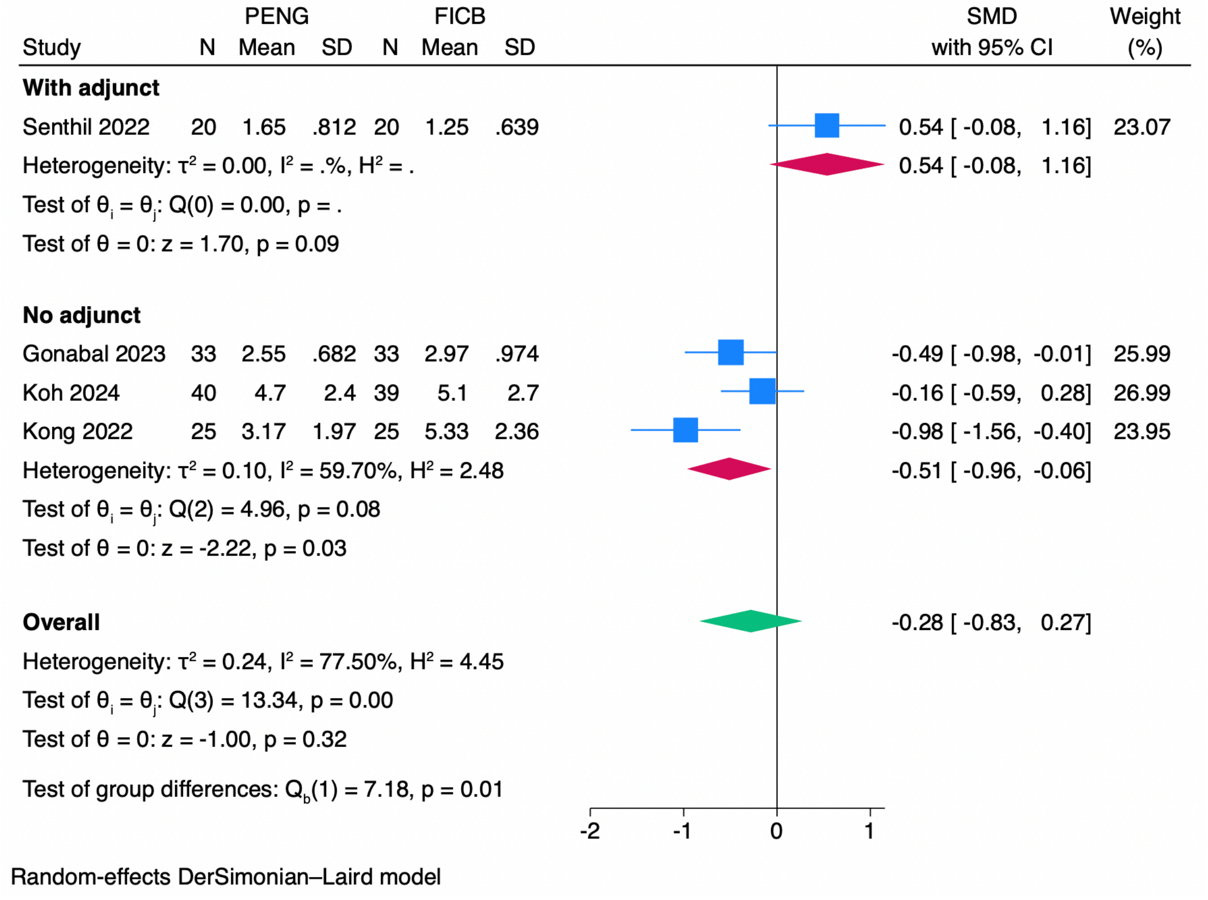

Dynamic pain at 24 hours postoperatively - leave-one-out analysis

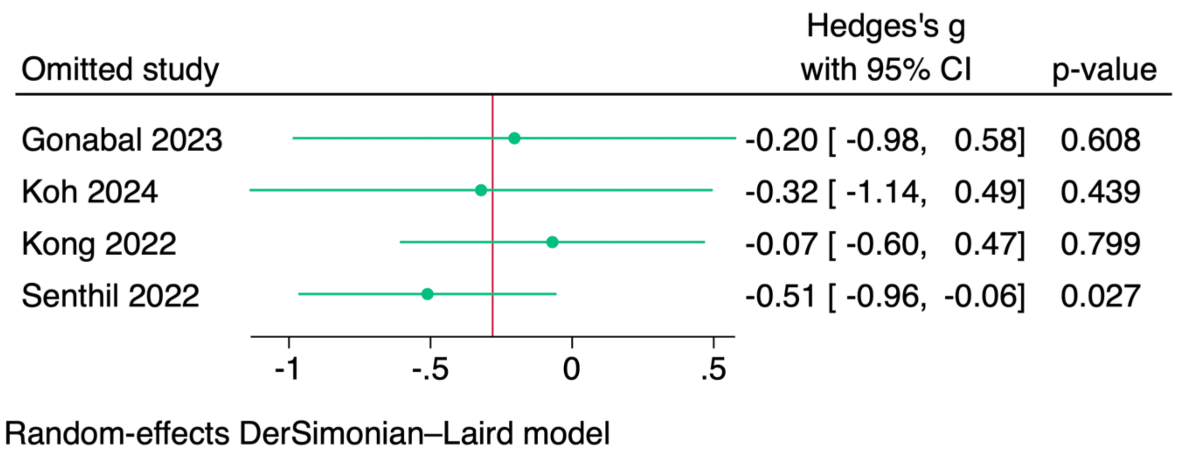

Consumption of oral morphine equivalents (OMEs) in 24 hours postoperatively - subgroup analyses

Consumption of OMEs in 24 hours postoperatively - timing of administration of the block (pre- or post-anaesthesia)

Study	PENG N	PENG Mean	PENG SD	FICB N	FICB Mean	FICB SD	MD with 95% CI	Weight (%)
**pre-anaesthesia**								
Bauiomy 2024	30	29	0.34	30	34.5	0.51	-5.50 [-5.72, -5.28]	13.39
Jadon 2021	33	20	0.21	33	16	0.20	4.00 [3.90, 4.10]	13.39
Koh 2024	40	83	46.50	39	82.75	60.00	0.25 [-23.39, 23.89]	7.80
Mosaffaa 2022	30	135	64.17	22	186	47.17	-51.00 [-82.72, -19.28]	5.85
Yadav 2023	25	141	60.83	25	191	36.35	-50.00 [-77.78, -22.22]	6.73
Heterogeneity: $\tau^2 = 45.27$, $I^2 = 99.93\%$, $H^2 = 1519.32$							-7.07 [-15.30, 1.16]	
Test of $\theta_i = \theta_j$: Q(4) = 6077.28, p = 0.00								
Test of $\theta = 0$: z = -1.68, p = 0.09								
**post-anaesthesia**								
Derakhshan 2024	25	23	10.75	25	21	10.00	2.00 [-3.76, 7.76]	12.84
Gonabal 2023	33	13	0.02	33	31	0.17	-18.00 [-18.06, -17.94]	13.39
Reddy 2022	20	33	1.65	20	50	3.30	-17.00 [-18.62, -15.38]	13.34
Senthil 2021	22	36	3.33	21	43	5.33	-7.00 [-9.64, -4.36]	13.27
Heterogeneity: $\tau^2 = 26.10$, $I^2 = 97.38\%$, $H^2 = 38.10$							-10.82 [-16.05, -5.59]	
Test of $\theta_i = \theta_j$: Q(3) = 114.29, p = 0.00								
Test of $\theta = 0$: z = -4.05, p = 0.00								
**Overall**							-11.88 [-22.09, -1.66]	
Heterogeneity: $\tau^2 = 202.94$, $I^2 = 99.99\%$, $H^2 = 17742.02$								
Test of $\theta_i = \theta_j$: Q(8) = 141936.17, p = 0.00								
Test of $\theta = 0$: z = -2.28, p = 0.02								
Test of group differences: $Q_b(1) = 0.57$, p = 0.45								

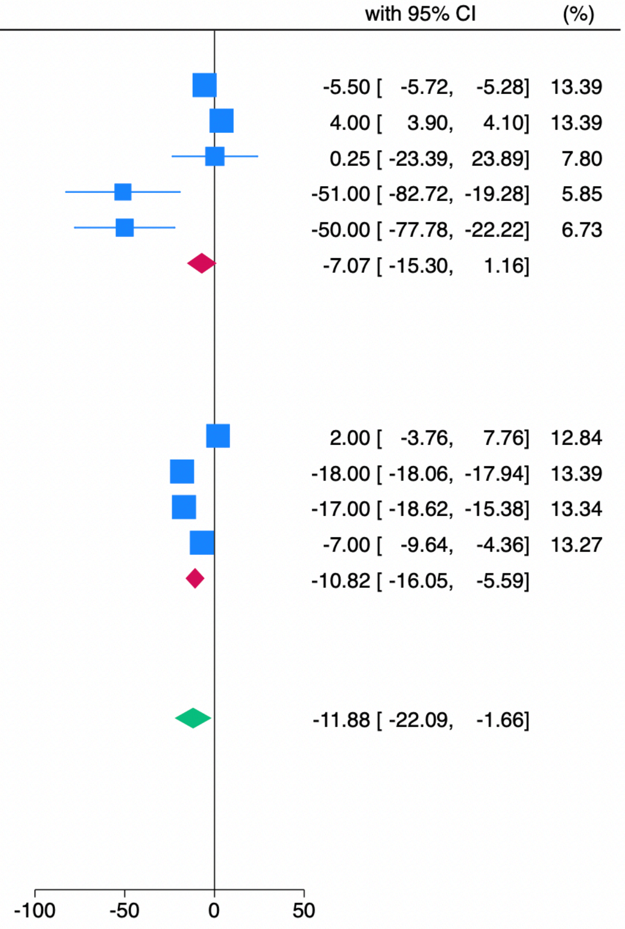

Random-effects DerSimonian–Laird model

Consumption of OMEs in 24 hours postoperatively - low risk of bias

Study	PENG N	PENG Mean	PENG SD	FICB N	FICB Mean	FICB SD	MD with 95% CI	Weight (%)
**Low RoB**								
Bauiomy 2024	30	29	0.34	30	34.5	0.51	-5.50 [-5.72, -5.28]	13.39
Koh 2024	40	83	46.50	39	82.75	60.00	0.25 [-23.39, 23.89]	7.80
Senthil 2021	22	36	3.33	21	43	5.33	-7.00 [-9.64, -4.36]	13.27
Heterogeneity: $\tau^2 = 0.00$, $I^2 = 0.00\%$, $H^2 = 1.00$							-5.51 [-5.73, -5.29]	
Test of $\theta_i = \theta_j$: Q(2) = 1.46, p = 0.48								
Test of $\theta = 0$: z = -49.85, p = 0.00								
**Moderate or high**								
Derakhshan 2024	25	23	10.75	25	21	10.00	2.00 [-3.76, 7.76]	12.84
Gonabal 2023	33	13	0.02	33	31	0.17	-18.00 [-18.06, -17.94]	13.39
Jadon 2021	33	20	0.21	33	16	0.20	4.00 [3.90, 4.10]	13.39
Mosaffaa 2022	30	135	64.17	22	186	47.17	-51.00 [-82.72, -19.28]	5.85
Reddy 2022	20	33	1.65	20	50	3.30	-17.00 [-18.62, -15.38]	13.34
Yadav 2023	25	141	60.83	25	191	36.35	-50.00 [-77.78, -22.22]	6.73
Heterogeneity: $\tau^2 = 241.02$, $I^2 = 100.00\%$, $H^2 = 27646.74$							-16.19 [-29.81, -2.56]	
Test of $\theta_i = \theta_j$: Q(5) = 138233.68, p = 0.00								
Test of $\theta = 0$: z = -2.33, p = 0.02								
**Overall**							-11.88 [-22.09, -1.66]	
Heterogeneity: $\tau^2 = 202.94$, $I^2 = 99.99\%$, $H^2 = 17742.02$								
Test of $\theta_i = \theta_j$: Q(8) = 141936.17, p = 0.00								
Test of $\theta = 0$: z = -2.28, p = 0.02								
Test of group differences: $Q_b(1) = 2.36$, p = 0.12								

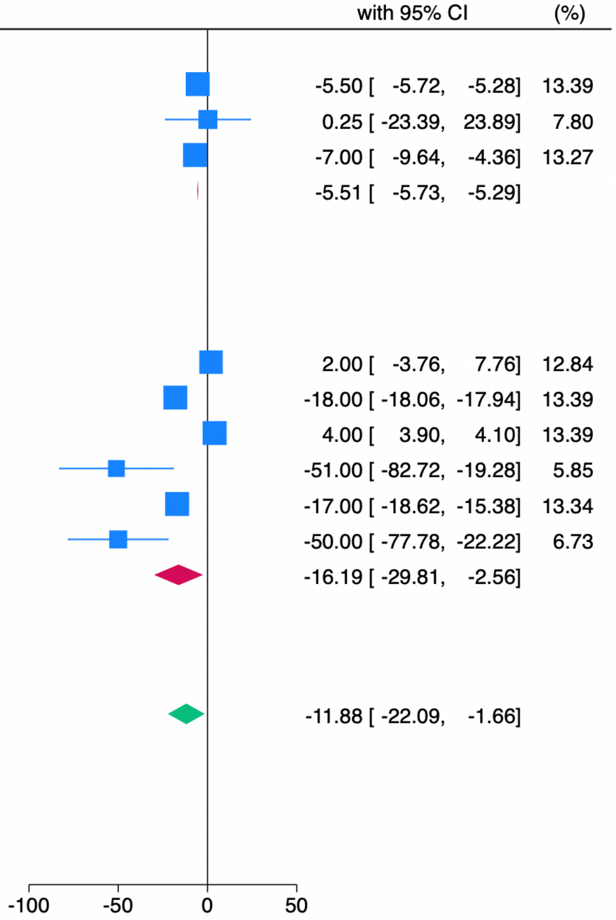

Random-effects DerSimonian–Laird model

Consumption of OMEs in 24 hours postoperatively - supra-inguinal approach of FICB vs infra-inguinal or unknown

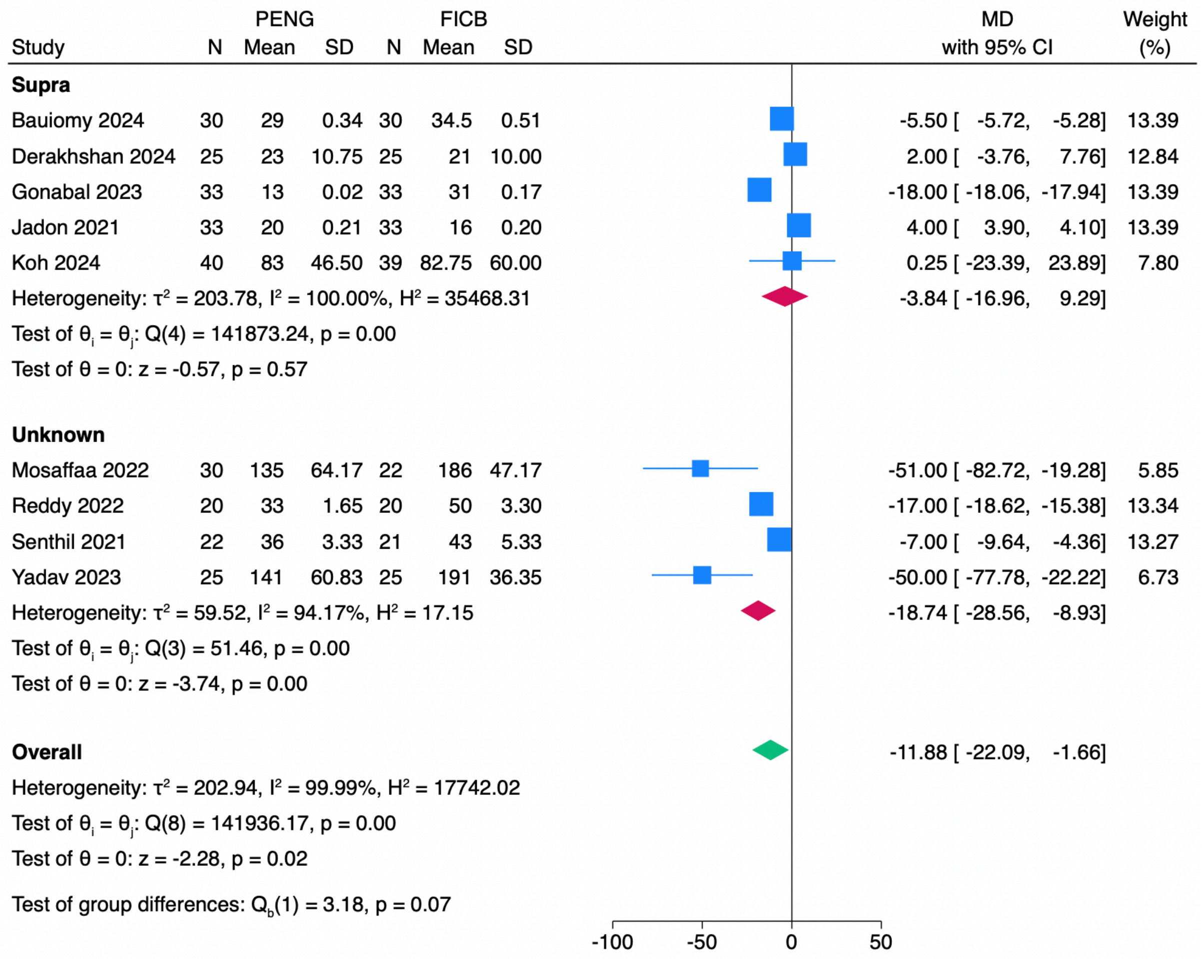

Random-effects DerSimonian–Laird model

Consumption of OMEs in 24 hours postoperatively - different volumes/doses of anaesthetic used in each group

Study	PENG N	PENG Mean	PENG SD	FICB N	FICB Mean	FICB SD	MD with 95% CI	Weight (%)
**Same volume**								
Bauiomy 2024	30	29	0.34	30	34.5	0.51	-5.50 [-5.72, -5.28]	13.39
Derakhshan 2024	25	23	10.75	25	21	10.00	2.00 [-3.76, 7.76]	12.84
Gonabal 2023	33	13	0.02	33	31	0.17	-18.00 [-18.06, -17.94]	13.39
Jadon 2021	33	20	0.21	33	16	0.20	4.00 [3.90, 4.10]	13.39
Mosaffaa 2022	30	135	64.17	22	186	47.17	-51.00 [-82.72, -19.28]	5.85
Reddy 2022	20	33	1.65	20	50	3.30	-17.00 [-18.62, -15.38]	13.34
Senthil 2021	22	36	3.33	21	43	5.33	-7.00 [-9.64, -4.36]	13.27
Yadav 2023	25	141	60.83	25	191	36.35	-50.00 [-77.78, -22.22]	6.73
Heterogeneity: $\tau^2 = 202.95$, $I^2 = 100.00\%$, $H^2 = 20276.45$							-12.90 [-23.54, -2.26]	
Test of $\theta_i = \theta_j$: Q(7) = 141935.12, p = 0.00								
Test of $\theta = 0$: z = -2.38, p = 0.02								
**Different volume**								
Koh 2024	40	83	46.50	39	82.75	60.00	0.25 [-23.39, 23.89]	7.80
Heterogeneity: $\tau^2 = 0.00$, $I^2 = .\%$, $H^2 = .$							0.25 [-23.39, 23.89]	
Test of $\theta_i = \theta_j$: Q(0) = 0.00, p = .								
Test of $\theta = 0$: z = 0.02, p = 0.98								
**Overall**							-11.88 [-22.09, -1.66]	
Heterogeneity: $\tau^2 = 202.94$, $I^2 = 99.99\%$, $H^2 = 17742.02$								
Test of $\theta_i = \theta_j$: Q(8) = 141936.17, p = 0.00								
Test of $\theta = 0$: z = -2.28, p = 0.02								
Test of group differences: $Q_b(1) = 0.99$, p = 0.32								

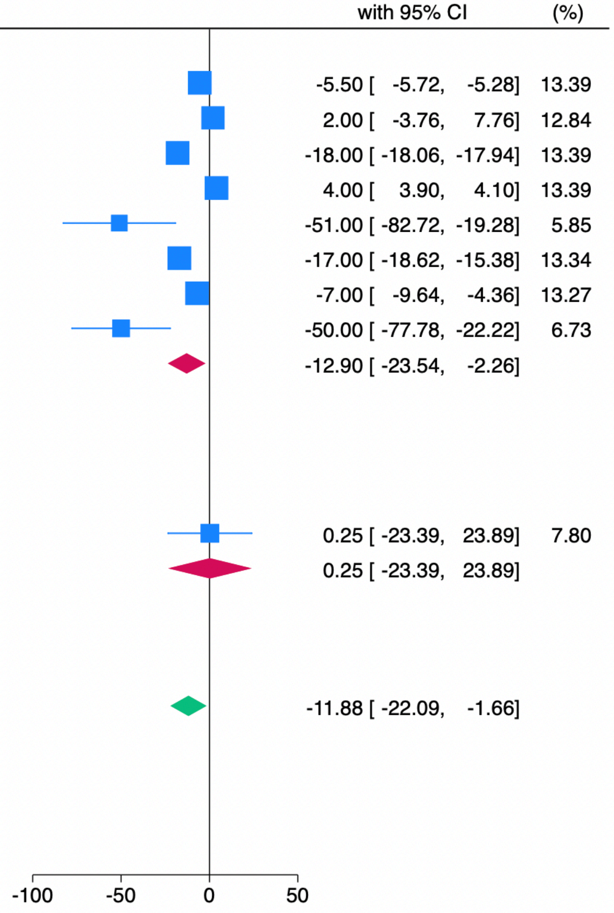

Random-effects DerSimonian–Laird model

Consumption of OMEs in 24 hours postoperatively - spinal or general anaesthetic

Study	PENG N	PENG Mean	PENG SD	FICB N	FICB Mean	FICB SD	MD with 95% CI	Weight (%)
**SA**								
Bauiomy 2024	30	29	0.34	30	34.5	0.51	-5.50 [-5.72, -5.28]	13.39
Derakhshan 2024	25	23	10.75	25	21	10.00	2.00 [-3.76, 7.76]	12.84
Gonabal 2023	33	13	0.02	33	31	0.17	-18.00 [-18.06, -17.94]	13.39
Jadon 2021	33	20	0.21	33	16	0.20	4.00 [3.90, 4.10]	13.39
Koh 2024	40	83	46.50	39	82.75	60.00	0.25 [-23.39, 23.89]	7.80
Mosaffaa 2022	30	135	64.17	22	186	47.17	-51.00 [-82.72, -19.28]	5.85
Reddy 2022	20	33	1.65	20	50	3.30	-17.00 [-18.62, -15.38]	13.34
Senthil 2021	22	36	3.33	21	43	5.33	-7.00 [-9.64, -4.36]	13.27
Yadav 2023	25	141	60.83	25	191	36.35	-50.00 [-77.78, -22.22]	6.73
Heterogeneity: $\tau^2 = 202.94$, $I^2 = 99.99\%$, $H^2 = 17742.02$							-11.88 [-22.09, -1.66]	
Test of $\theta_i = \theta_j$: Q(8) = 141936.17, p = 0.00								
Test of $\theta = 0$: z = -2.28, p = 0.02								
**Overall**							-11.88 [-22.09, -1.66]	
Heterogeneity: $\tau^2 = 202.94$, $I^2 = 99.99\%$, $H^2 = 17742.02$								
Test of $\theta_i = \theta_j$: Q(8) = 141936.17, p = 0.00								
Test of $\theta = 0$: z = -2.28, p = 0.02								
Test of group differences: $Q_b(0) = 0.00$, p = .								

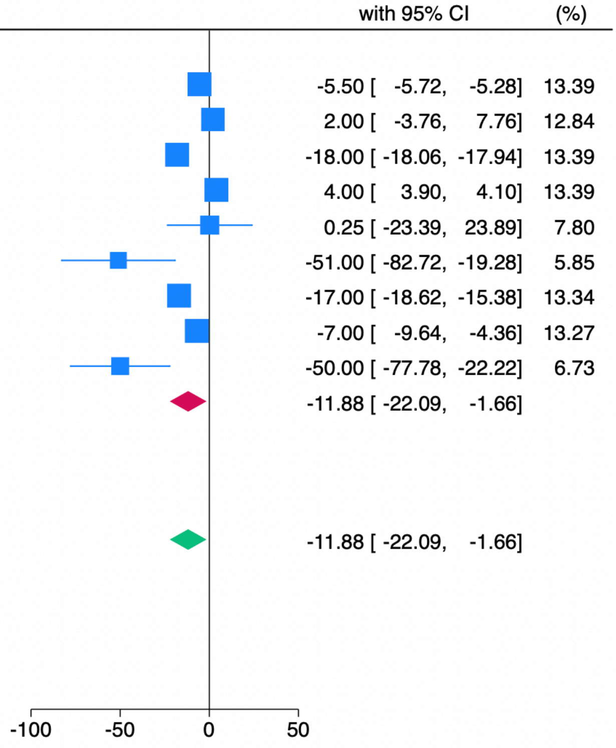

Random-effects DerSimonian–Laird model

Consumption of OMEs in 24 hours postoperatively - block adjunct use

Study	PENG N	PENG Mean	PENG SD	FICB N	FICB Mean	FICB SD	MD with 95% CI	Weight (%)
**With adjunct**								
Bauiomy 2024	30	29	0.34	30	34.5	0.51	-5.50 [-5.72, -5.28]	13.39
Jadon 2021	33	20	0.21	33	16	0.20	4.00 [3.90, 4.10]	13.39
Reddy 2022	20	33	1.65	20	50	3.30	-17.00 [-18.62, -15.38]	13.34
Senthil 2021	22	36	3.33	21	43	5.33	-7.00 [-9.64, -4.36]	13.27
Heterogeneity: $\tau^2 = 48.30$, $I^2 = 99.95\%$, $H^2 = 2215.84$							-6.33 [-13.19, 0.52]	
Test of $\theta_i = \theta_j$: Q(3) = 6647.52, p = 0.00								
Test of $\theta = 0$: z = -1.81, p = 0.07								
**No adjunct**								
Derakhshan 2024	25	23	10.75	25	21	10.00	2.00 [-3.76, 7.76]	12.84
Gonabal 2023	33	13	0.02	33	31	0.17	-18.00 [-18.06, -17.94]	13.39
Koh 2024	40	83	46.50	39	82.75	60.00	0.25 [-23.39, 23.89]	7.80
Mosaffaa 2022	30	135	64.17	22	186	47.17	-51.00 [-82.72, -19.28]	5.85
Yadav 2023	25	141	60.83	25	191	36.35	-50.00 [-77.78, -22.22]	6.73
Heterogeneity: $\tau^2 = 204.86$, $I^2 = 93.10\%$, $H^2 = 14.48$							-18.22 [-33.24, -3.20]	
Test of $\theta_i = \theta_j$: Q(4) = 57.93, p = 0.00								
Test of $\theta = 0$: z = -2.38, p = 0.02								
**Overall**							-11.88 [-22.09, -1.66]	
Heterogeneity: $\tau^2 = 202.94$, $I^2 = 99.99\%$, $H^2 = 17742.02$								
Test of $\theta_i = \theta_j$: Q(8) = 141936.17, p = 0.00								
Test of $\theta = 0$: z = -2.28, p = 0.02								
Test of group differences: $Q_b(1) = 1.99$, p = 0.16								

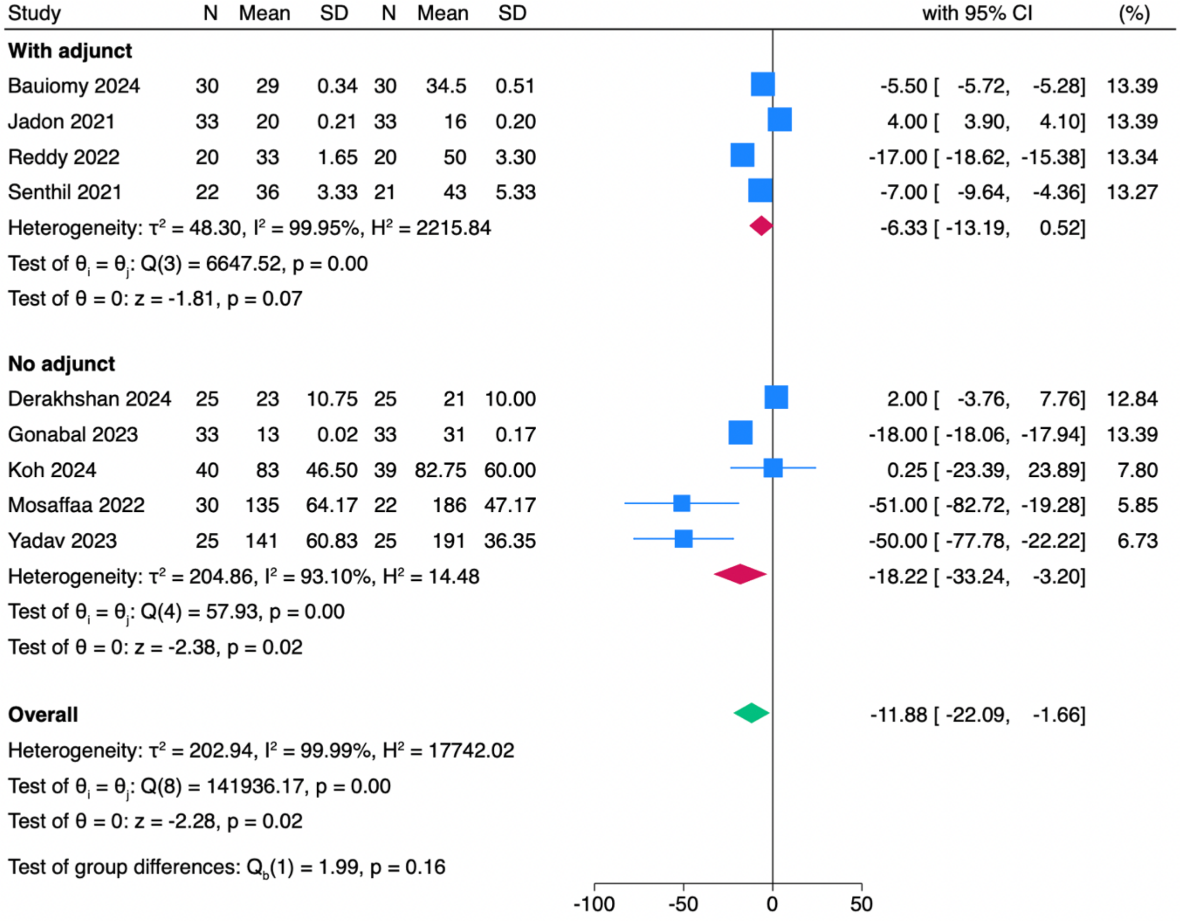

Random-effects DerSimonian–Laird model

Consumption of OMEs in 24 hours postoperatively - patient-controlled analgesia (PCA) pump use

Study	PENG N	PENG Mean	PENG SD	FICB N	FICB Mean	FICB SD	SMD with 95% CI	Weight (%)
**PCA**								
Koh 2024	40	83	46.50	39	82.75	60.00	0.25 [-23.39, 23.89]	7.80
Mosaffaa 2022	30	135	64.17	22	186	47.17	-51.00 [-82.72, -19.28]	5.85
Senthil 2021	22	36	3.33	21	43	5.33	-7.00 [-9.64, -4.36]	13.27
Yadav 2023	25	141	60.83	25	191	36.35	-50.00 [-77.78, -22.22]	6.73
Heterogeneity: τ^2 = 447.51, I^2 = 82.11%, H^2 = 5.59							-23.92 [-47.57, -0.26]	
Test of $\theta_i = \theta_j$: Q(3) = 16.77, p = 0.00								
Test of θ = 0: z = -1.98, p = 0.05								
**Request**								
Derakhshan 2024	25	23	10.75	25	21	10.00	2.00 [-3.76, 7.76]	12.84
Jadon 2021	33	20	0.21	33	16	0.20	4.00 [3.90, 4.10]	13.39
Heterogeneity: τ^2 = 0.00, I^2 = 0.00%, H^2 = 1.00							4.00 [3.90, 4.10]	
Test of $\theta_i = \theta_j$: Q(1) = 0.46, p = 0.50								
Test of θ = 0: z = 78.25, p = 0.00								
**Unknown**								
Bauiomy 2024	30	29	0.34	30	34.5	0.51	-5.50 [-5.72, -5.28]	13.39
Gonabal 2023	33	13	0.02	33	31	0.17	-18.00 [-18.06, -17.94]	13.39
Reddy 2022	20	33	1.65	20	50	3.30	-17.00 [-18.62, -15.38]	13.34
Heterogeneity: τ^2 = 76.72, I^2 = 99.98%, H^2 = 5924.03							-13.49 [-23.42, -3.56]	
Test of $\theta_i = \theta_j$: Q(2) = 11848.05, p = 0.00								
Test of θ = 0: z = -2.66, p = 0.01								
**Overall**							-11.88 [-22.09, -1.66]	
Heterogeneity: τ^2 = 202.94, I^2 = 99.99%, H^2 = 17742.02								
Test of $\theta_i = \theta_j$: Q(8) = 141936.17, p = 0.00								
Test of θ = 0: z = -2.28, p = 0.02								
Test of group differences: $Q_b(2)$ = 17.27, p = 0.00								

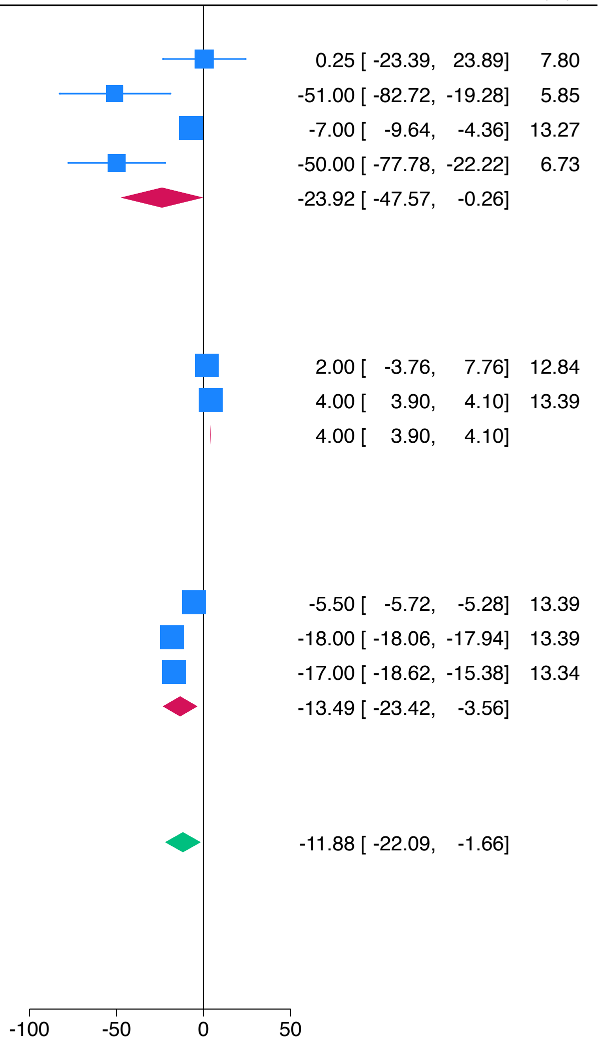

Random-effects DerSimonian–Laird model

Consumption of OMEs in 24 hours postoperatively - leave-one-out analysis

Omitted study	Mean diff. with 95% CI	p-value
Bauiomy 2024	-13.25 [-25.08, -1.42]	0.028
Derakhshan 2024	-13.92 [-24.87, -2.98]	0.013
Gonabal 2023	-7.58 [-13.44, -1.71]	0.011
Jadon 2021	-12.37 [-19.55, -5.18]	0.001
Koh 2024	-12.90 [-23.54, -2.26]	0.017
Mosaffaa 2022	-9.45 [-19.98, 1.08]	0.079
Reddy 2022	-11.10 [-22.09, -0.11]	0.048
Senthil 2021	-12.63 [-23.60, -1.65]	0.024
Yadav 2023	-9.13 [-19.71, 1.45]	0.091

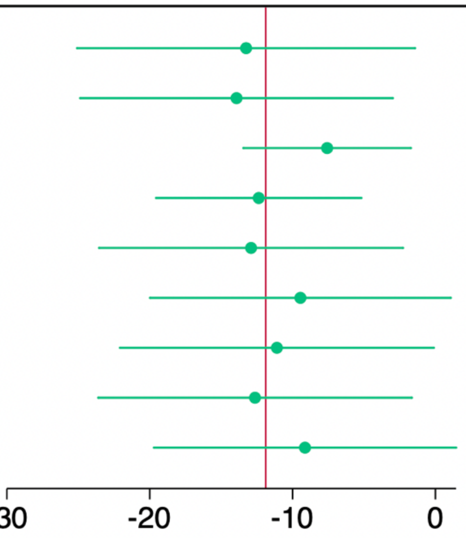

Random-effects DerSimonian–Laird model

Consumption of OMEs in 24 hours postoperatively - outlier analysis

Study	PENG Mean	PENG SD	PENG Total	FICB Mean	FICB SD	FICB Total	MD with 95% CI	Weight (%)
Bauiomy 2024	29	0.34	30	34.5	0.51	30	-5.50 [-5.72, -5.28]	18.38
Derakhshan 2024	23	10.75	25	21	10.00	25	2.00 [-3.76, 7.76]	16.53
Gonabal 2023	13	0.02	33	31	0.17	33	-18.00 [-18.06, -17.94]	18.39
Koh 2024	83	46.50	40	82.75	60.00	39	0.25 [-23.39, 23.89]	6.35
Mosaffaa 2022	135	64.17	30	186	47.17	22	-51.00 [-82.72, -19.28]	4.17
Reddy 2022	33	1.65	20	50	3.30	20	-17.00 [-18.62, -15.38]	18.22
Senthil 2021	36	3.33	22	43	5.33	21	-7.00 [-9.64, -4.36]	17.96
**Overall**							-10.45 [-17.82, -3.09]	

Heterogeneity: $\tau^2 = 76.75$, $I^2 = 99.95\%$, $H^2 = 1992.29$

Test of $\theta_i = \theta_j$: Q(6) = 11953.72, p = 0.00

Test of $\theta = 0$: z = -2.78, p = 0.01

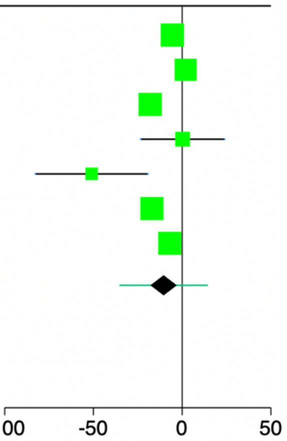

Random-effects DerSimonian–Laird model
95% prediction interval

Table of Drug Conversions to Oral Morphine Equivalents (OMEs)

Conversion factors to OME:

- Tramadol IV: 0.25
- Morphine IV: 2.5
- Fentanyl: 0.165

Study ID (Author, Year)	Reported opioid metric	Drug(s) reported	Route(s) reported	Intervention 1 formula to convert	Intervention 2 formula to convert	Intervention 1 mean OME in 24h	Intervention 2 mean OME in 24h	Assumptions made? (Y/N, details)	Imputed? (Y/N, details)	Included in main analysis? (Y/N)
**Bauiomy et al, 2024**	Number of tramadol doses (24h)	1 dose = 50mg IV tramadol	IV	(2.3 x 50) x 0.25	(2.7 x 50) x 0.25	29mg	34mg	N	N	Y
**Chaudhary et al, 2023**	Duration of analgesia consumption	Not reported	IV	N/A	N/A	N/A		N/A	N	N, paper did not report the post operative analgesia received. Only reported the mean duration that post operative analgesia was required
**Cui et al, 2024**	Number of parecoxib sodium and sufentanyl doses (24h)	1 dose = 40mg parecoxib 1.5 μg/L sufentanil	IV & PCA	N/A	N/A	N/A		N/A	N	N, paper reported "opioid time" , given the sufentanyl was provided via PCA, there was no reasonable way to impute or assume how many doses were recieved over the reported time
**Derakshan et al, 2024**	Total morphine consumption (24h)	IV morphine	IV	9.2 x 2.5	8.3 x 2.5	23mg	21mg	N	N	Y
**Gonobal et al, 2024**	Total opioid consumption (24h)	1 dose = 1 mg/kg tramadol	IV	(5 ÷ 0.1) x 0.25	(12.5 ÷ 0.1) x 0.25	13mg	31mg	N	Y, paper reported outcome as morphine equivalents (ME) and drug used was IV tramadol converted by a factor of 0.1. We reversed the calculation to gather the mean tramadol dose and applied the cited OME calculation we have used in this case to a factor of 0.165.	Y
**Jadon et al, 2021**	Number of tramadol doses (24h)	1 dose = 50mg tramadol	IV	(1.6 x 50) x 0.25	(1.3 x 50) x 0.25	20mg	16mg	N	N	Y
**Kadlimatti et al, 2024**	Number of tramadol doses (24h)	1 dose = 1 mg/kg tramadol	IV	N/A	N/A	N/A		N/A	N	N, In order to analyse the reported outcome, average weight and average dose would have to be used to calculate the received dose.
**Keskes et al, 2023**	Mean morphine consumption in post operative monitoring room (2h)	mg of morphine in 2 hours	IV	N/A	N/A	N/A		N/A	N	N, outcome of this paper relating to analgesic consumption is only during the 2h postoperative period thus not comparable. It would not be appropriate to extrapolate the data to a 24h period
**Koh et al, 2024**	IV morphine equivalents (24h)	not reported	IV & PCA	N/A	N/A	N/A	N/A	N/A	N	N, outcome reported using morphine equivalents (ME), the factor and drug derriving this are not given
**Kong et al, 2022**	Total dose of fentanyl (24h)	fentanyl in mcg / 24 hours	PCA	N/A	N/A	N/A		N/A	N	N, data relating to this outcome was given using median / interquartile range. Unable to determine mean without estimation
**Lin et al, 2024**	Total dose in "morphine equivalents" (24h)	Not reported	Not reported	N/A	N/A	N/A	N/A	N/A	N	N, outcome was reported as morphine equivalents in 24h but no information given as to what constituted that calculation (e.g. drug, dose and formula). Unable to assume equivalence with included outcomes
**Manohara et al, 2021**	Total dose of oxynorm (24h)	oxycodone (oxynorm)	Not reported	N/A	N/A	N/A	N/A	N/A	N	N, route of oxycodone not reported thus unable to calculate OME
**Mosaffa et al, 2022**	Number of morphine doses (24h)	1 dose via PCA = 1mg morphine, 1 dose via IV = 2mg morphine	IV & PCA	54 x 2.5	74.37 x 2.5	135mg	186mg	Y, Assumed unit of morphine given was in milligrams (mg) aligning to standard practice	N	Y
**Natjaran et al, 2021**	Number of paracetamol doses (24h)	1 dose = 1g paracetamol	IV	N/A	N/A	N/A	N/A	N/A	N	N, accepted formulae does not apply to calculation of paracetamol to OME
**Reddy et al, 2022**	Total fentanyl consumption (24h)	fentanyl in mcg / 24 hours	IV	200 x 0.165	300 x 0.165	33mg	50mg	Y, Fentanyl was given IV, this is not explicitly stated but in-keeping with usual practice	N	Y
**Saini et al, 2024**	Total tramadol and paracetamol consumption	paracetamol 1g, tramadol 50-100mg	IV & PCA	N/A		N/A		N	N	N, accepted formulae is not approved for calculation of paracetamol to OME. Paracetamol use is reported as given unless contraindicated, no report is made as to whther there were any contraindications. Somepatients therefore maay have received no paracetamol and only PCA tramadol. There is no reliable way to impute.
**Senthil et al, 2021**	Total fentanyl consumption (24h)	fentanyl in mcg / 24 hours	PCA	213 x 0.165	255 x 0.165	36mg	43mg	Y, Fentanyl was given IV, this is not explicitly stated but in-keeping with usual practice	N	Y
**Shankar et al, 2020**	Total tramadol consumption (24h)	1 dose = 1mg/kg	IV	N/A		N/A		N	N	N, In order to analyse the reported outcome, average weight and average dose would have to be used to calculate the received dose.
**Yadav et al, 2023**	Total morphine consumption (24h)	1 dose via PCA = 1mg morphine, 1 dose via IV = 2mg morphine	IV & PCA	56.36 x 2.5	76.23 x 2.5	141mg	191mg	N	N	Y
